# Epidemiological Trends in Cardiovascular Disease Mortality Attributable to Modifiable Risk Factors and Its Association with Sociodemographic Transitions across BRICS-Plus Countries

**DOI:** 10.3390/nu15173757

**Published:** 2023-08-28

**Authors:** Nawsherwan, Sumaira Mubarik, Wang Bin, Zhang Le, Mangmang Sang, Yijun Lin, Jinrong Zheng, Yan Wang

**Affiliations:** 1 Xiamen Cardiovascular Hospital of Xiamen University, School of Medicine, Xiamen University, Xiamen 361102, China; nawshermkd177@gmail.com (N.);; 2 PharmacoTherapy, Epidemiology and Economics, Groningen Research Institute of Pharmacy, University of Groningen, 9713 AV Groningen, The Netherlands; 3 Department of Epidemiology and Biostatistics, School of Public Health, Wuhan University, Wuhan 430071, China

**Keywords:** cardiovascular diseases, mortality, modifiable risk factors, sociodemographic index, BRICS-Plus

## Abstract

BRICS-Plus countries (Brazil, Russia, India, China, South Africa, and 30 other countries) is a group of 35 countries with emerging economies making up more than half of the world’s population. We explored epidemiological trends of cardiovascular disease (CVD) mortality attributable to modifiable risk factors and its association with period and birth cohort effects and sociodemographic index (SDI) across BRICS-Plus countries by using joinpoint regression and age-period-cohort modeling from 1990 to 2019. Between 1990 and 2019, the all-ages CVD deaths increased by 85.2% (6.1 million to 11.3 million) across BRICS-Plus countries. The CVD age-standardized mortality rate attributable to dietary risks and smoking significantly decreased across BRICS-Plus countries, with some exceptions. However, four-fifths of BRICS-Plus countries observed a remarkable increasing trend of high body mass-index (BMI)-related CVD deaths, in particular, among younger adults (25–49 years). Early birth cohorts and individuals aged greater than 50 years showed a higher risk of CVD mortality. Both the China-ASEAN FTA and Mercosur regions stand out for their successful sociodemographic transition, with a significant reduction in CVD mortality over the study period. Singapore and Brazil achieved great progress in CVD mortality reduction and the other BRICS-Plus countries should follow their lead in adopting public health policies and initiatives into practice.

## 1. Introduction

Cardiovascular disease (CVD) is the leading cause of premature mortality, responsible for 18.6 million deaths in 2019 worldwide. Cardiovascular diseases (CVDs), primarily ischemic heart disease (IHD) and stroke are the top-ranking cause of global mortality accounting for 9.1 million and 6.5 million deaths in the year 2019, respectively [1]. In 2030, the projected CVD-related deaths would be more than 23 million worldwide [2]. According to a World Health Organization estimate, low- and middle-income countries account for more than three-quarters of CVD deaths, which, in recent years, has been seen as a growing epidemic problem [3]. 

BRICS countries (Brazil, Russia, India, China, and South Africa) are a group of countries with rapid politico-economic development and comprise nearly half of the world population [4]. BRICS-Plus countries is the extension of BRICS countries consisting of 35 developing countries which aims to bring together the regional integration blocks, in which the BRICS countries’ economies play a major role, and attempts to provide a new platform for forming regional and bilateral alliances across continents [5]. From 1992 to 2017, the high age-standardized mortality rate (ASMR) for CVD has been reported across the BRICS countries attributable to cardiometabolic and behavioral risk factors [4,6,7,8]. In 2013, the United Nations set a target to reduce 25% premature CVD mortality by 2025, attributable to behavioral and biological risk factors [9]. In 2014, the BRICS countries reaffirmed their commitment to the prevention and control of non-communicable diseases, particularly CVD [4].

Modifiable risk factors including dietary risks, high body mass index (BMI), and smoking are major drivers of CVD. In the Global Burden of Disease (GBD) study 2019, dietary risks are either an over-consumed diet (sodium, trans-fatty acids, sugar-sweetened beverages, red meat, and processed meat) or an under-consumed diet (whole grains, legumes, vegetables, fruits, nuts and seeds, milk, fiber, calcium, omega-3 fatty acids from seafood, and polyunsaturated fatty acids) [1,10]. In 2019, CVDs were the primary consequence of these dietary risks, which caused 7.94 million annual deaths. High BMI (≥25 kg/m^2^) is considered an epidemic worldwide. High BMI exacerbates CVD risk factors including hypertension, high plasma lipids, high plasma glucose, and inflammation, and has a linear association with coronary heart diseases. Globally, 5.02 million and 2.7 million CVD deaths are attributed to high BMI and primary smoking in 2019, respectively [1]. 

A previous study has focused on time trends and age-period-cohort effect on cause-specific CVD mortality across BRICS countries from 1992 to 2016. However, this study fails to report CVD mortality attributable to modifiable risk factors and its association with socio-demographic transition across BRICS countries [4]. To the best of our knowledge, no previous study has reported temporal trends of CVD mortality and its association with the sociodemographic index (SDI) across BRICS-Plus countries. Therefore, this study explores epidemiological trends of CVD mortality attributable to modifiable risk factors and its association with SDI across BRICS-Plus countries. These consistent and comparable analyses of long-term trends and patterns of CVD mortality may provide references to health policymakers and decision-makers to allocate their healthcare resources and specify prevention and control strategies at regional and country levels across BRICS-Plus countries.

## 2. Materials and Methods

### 2.1. Data Source

In this study, the data were extracted by sex (male, female, and both sex combined) from the Global Burden of Disease (GBD) free online database (GBD 2019, http://ghdx.healthdata.org/gbd-results-tool) [11] (accessed on 6 June 2022) from 1990 to 2019. In addition, age-specific data for modifiable risk factors (i.e., 25–49 years, 50–69 years, and 70–89 years) and cause-specific data were extracted for different age groups (i.e., from 25–49 years to 85^+^ years). GBD is an international cooperative project that estimates the disease burden at regional, national, and global levels. GDB estimates the burden of disease indices including, prevalence, incidence, mortality rate, years of life lost, years lived with disability, and disability-adjusted life years for several diseases and injuries. Moreover, the GBD data are provided by different organizations like World Bank Open Data, WHO, and Global Health Observatory for different political and social research. The GBD data are managed by the Institute for Health Metrics and Evaluation (IHME), University of Washington. Therefore, a waiver of informed consent was reviewed and approved by the University of Washington Institutional Review Board [12,13].

### 2.2. Variables Understudy

In the present study, the considered modifiable risk factors were dietary risks, high BMI, and smoking. The dietary risk factor is defined as the suboptimal consumption of whole grains, legumes, vegetables, fruits, nuts and seeds, milk, fiber, calcium, omega-3 fatty acids from seafood, and polyunsaturated fatty acids and the overconsumption of sodium, trans-fatty acids, sugar-sweetened beverages, red meat, and processed meat [1]. The outcome variables were ASMR and death numbers of CVD, IHD, and IS for the 35 BRICS-Plus countries from 1990 to 2019. The BRICS-Plus countries were divided into five regions including the South African Customs Union (SACU) region (i.e., Botswana, Eswatini, Lesotho, Namibia, and South Africa), South Asian Association for Regional Cooperation (SAARC) region (i.e., Afghanistan, Bangladesh, Bhutan, India, Maldives, Nepal, Pakistan, and Sri Lanka), China-ASEAN Free Trade Area (China-ASEAN FTA) region (i.e., Brunei Darussalam, Cambodia, China, Indonesia, Laos, Malaysia, Myanmar, Philippines, Singapore, Thailand, and Viet Nam), Eurasian Economic Union (EEU) region (i.e., Armenia, Belarus, Kazakhstan, Kyrgyzstan, and Russia), and Mercosur region (i.e., Argentina, Bolivia, Brazil, Paraguay, Uruguay, and Venezuela) [5]. 

### 2.3. Statistical Analysis

#### 2.3.1. Joinpoint Regression for Trend Analysis (1990–2019)

To assess the temporal trends of CVD, IHD, and IS burden, we estimated the average annual percentage change (AAPC) for CVD, IHD, and IS mortality with joinpoint regression analysis. AAPC represents the trend of CVD, IHD, and IS burden in the whole period from 1990 to 2019. Additionally, AAPC is a weighted average of the yearly percentage change determined by the joinpoint model, with weights corresponding to the duration of the annual percentage change (APC) interval. The APC shows the CVD, IHD, and IS burden trend in each segment determined by using joinpoint regression software. From 1990 to 2019, we produced AAPCs and their 95% confidence intervals (CIs) for each trend segment identified by the model. Furthermore, we estimated AAPCs of CVD, IHD, and IS deaths for both males and females. Based on age groups (i.e., 25–49, 50–69, and 70–89 years), AAPC for CVD, IHD, and IS burden was obtained for both sexes combined from 1990 to 2019. AAPC is considered significant when it is different from 0 at the alpha of 0.05. This analysis was conducted using the joinpoint regression program version 4.9.1.0 (April 2022) from the Surveillance Research Program of the U.S. National Cancer Institute (NCI). 

#### 2.3.2. Estimation of Age Period Cohort Effects on CVD Mortality

The relationship between mortality of CVD and each of the three main sources of spatial and temporal variability—age, period (year), and cohort (year of birth)—was examined for each BRICS-Plus region/country within the framework of the age-period-cohort model. Period effects show population-wide exposure at a specific moment, and the period was defined as the survey year. The term “cohort effects” refers to variations in hazards among birth cohorts. Age-specific mortality rates from 25 to 89 years with subsequent 5-year age intervals, calendar time including the consecutive period from 1990 to 2019, and subsequent cohort (period-age) from 1901 to 1994 were taken into consideration for statistical analysis in the age-period-cohort study. The R package (Epi, version 2.44) created by Carstensen et al. was used for the age-period-cohort analysis [14]. The Maximum Likelihood (ML) of the age-period-cohort model Poisson with log (Y) based on the natural-spline function was used to estimate the rate ratio. The reference cohort and reference period were respectively chosen based on the median date of birth and the median date of diagnosis among cases. The estimated models’ deviation table was used to assess the goodness of fit. Additionally, the Spearman correlation coefficient (r) was used to examine the relationship between Socio-Demographic Index (SDI) and CVD mortality. SDI is a geometric mean value range from 0.0 to 1.0 and is calculated from the total fertility rate under 25 years (TFRU25), lag-distributed income per capita (LDI), and average educational attainment in the population older than 15 years [15]. Two-sided statistical tests were taken into consideration. The threshold for statistical significance of the findings was set at a 1% level of significance.

## 3. Results

### 3.1. Trends of Cause-Specific CVD Mortality

**Figure 1:**
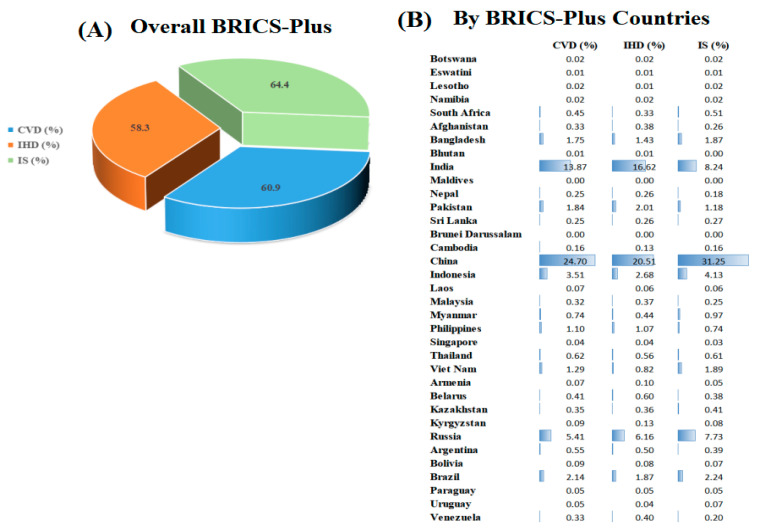
Relative proportion of BRICS-Plus countries to the global burden of cause-specific cardiovascular disease (CVD), ischemic heart disease (IHD), and ischemic stroke (IS) deaths in 2019. Relative proportion (%) = Deaths caused by CVD, IHD, and IS across the BRICS-Plus countries in 2019/Deaths caused by CVD, IHD, and IS across the world in 2019.

Table 1 shows trends of cause-specific CVD mortality across BRICS-Plus countries. In 2019, across the BRICS-Plus countries, there were 11.3 million CVD deaths, accounting for 60.9% of global CVD deaths and the ASMR of CVD was 296 per 100,000. The ASMR of CVD (367 to 296 per 100,000), IHD (169 to 143 per 100,000), and IS (66 to 55 per 100,000) across the BRICS-Plus countries decreased by 19.3%, 15.3%, and 16.6%, respectively. However, the number of CVD deaths increased by 85.2% (6.1 to 11.3 million) in BRICS-Plus countries. The number of IHD deaths (2.4 to 5.3 million) and the number of IS deaths (1.0 to 2.1 million) increased by 120.8% and 110% from 1990 to 2019 and accounting for 58.3% and 64.4% of global IHD and IS deaths in 2019, respectively (Figure 1).

From 1990 to 2019, despite a 38% reduction in CVD ASMR in China (381 to 276 per 100,000) and 22.8% in India (332 to 256 per 100,000), both countries had a marked increase in the total number of CVD mortality: an increase of 89% in China (2.4 to 4.5 million) and 114.3% in India (1.2 to 2.5 million). Lesotho, Pakistan, Indonesia, and the Philippines were the only BRICS-Plus countries with an increase in ASMR for CVD, IHD, and IS. Singapore had the largest decline in ASMR for CVD (65.6%) and IHD (65.3%) and Brazil (CVD; 50.7% and IHD; 52.8%) from 1990 to 2019 (Table 1, Tables S1 and S2).

### 3.2. Trends of CVD Mortality Attributable to Modifiable Risk Factors

**Figure 2:**
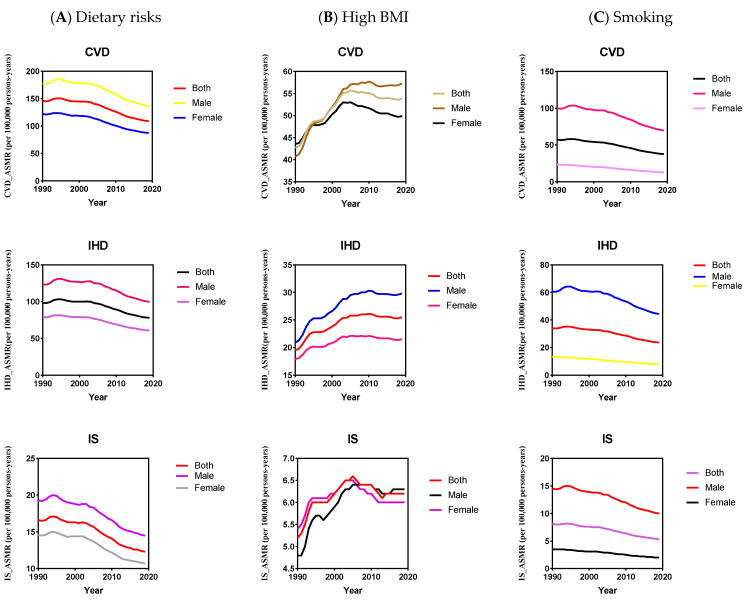
The temporal trend of cardiovascular disease (CVD), ischemic heart disease (IHD), and ischemic stroke (IS) burden (age-standardized mortality rate) for different gender attributable to dietary risks (**A**), high body mass index (BMI) (**B**), and smoking (**C**) across BRICS-Plus countries from 1990 to 2019.

For both sexes combined, between 1990 and 2019, despite a 25% reduction in diet-related ASMR for CVD (146 to 109 per 100,000), the dietary risk is the leading attributable risk to high CVD ASMR (109 [95% UI 81–142] than high BMI (53 [95% UI 31–80] and smoking (37 [95% UI 30–45] across BIRCS-plus countries in 2019. The ASMR for CVD attributable to high BMI increased by 26.2% (42.7 to 53.9 per 100,000). However, the trend of CVD ASMR due to smoking is remarkably decreased by 34.4% (57.3 to 37.6 per 100,000), indicating improvement in smoking-related CVD mortality (Figure 2).

**Figure 3:**
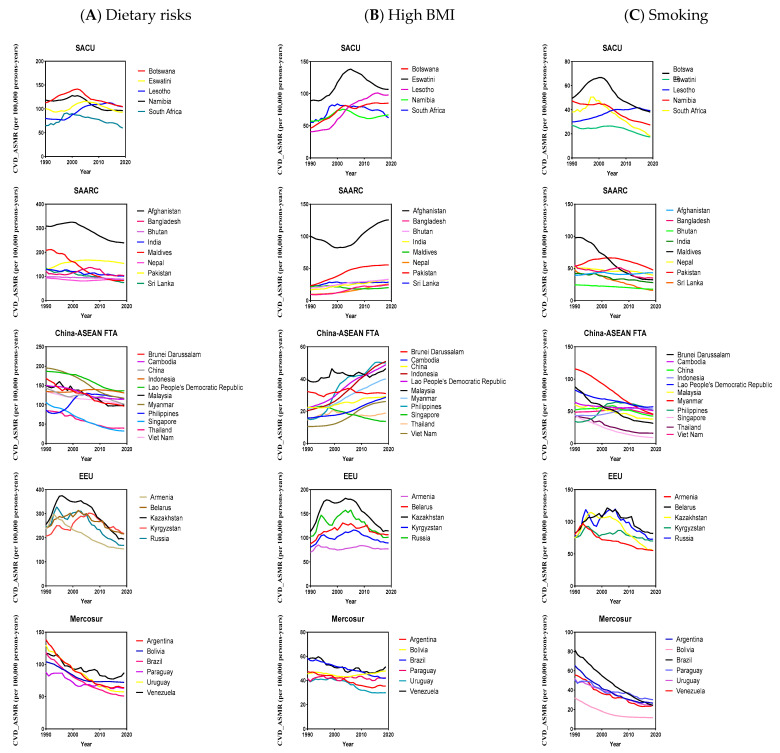
The temporal trend of cardiovascular diseases (CVD)_ASMR (age-standardized mortality rate) for both sexes attributable to dietary risks (**A**), high body mass index (BMI) (**B**), and smoking (**C**) across BRICS-Plus countries from 1990 to 2019, South African Customs Union (SACU), South Asian Association for Regional Cooperation (SAARC), China-ASEAN Free Trade Area (China-ASEAN FTA), and Eurasian Economic Union (EEU).

In 2019, among the 35 BRICS-Plus countries, Afghanistan had the highest diet-related CVD ASMR (238 [95% UI 185–294] and BMI-related CVD ASMR (125 [95% UI 75–185], and Belarus had the highest smoking-related CVD ASMR (81 [95% UI 65–102]. However, Singapore had the lowest diet-related ASMR for CVD (31 [95% UI 23–42], the lowest high BMI-related ASMR for CVD (13 [95% UI 7–20], and smoking-related ASMR for CVD (9 [95% UI 8–9] (Figure S1A). Moreover, from 1990 to 2019, Singapore had a substantial reduction in CVD ASMR attributable to dietary risks, with an average annual percent change (AAPC) −4.1% (95% CI, −4.3, −3.8), high BMI (AAPC −1.6% [95% CI, −1.9, −1.4], and smoking (AAPC −5.3% [95% CI, −5.6, −5.0]. Philippines observed a significant increase in CVD ASMR attributable to dietary risks (AAPC 1.1% [95% CI, 0.7, 1.6], high BMI (AAPC 4.3% [95% CI, 3.5, 5.1], and smoking (AAPC 1.5% [95% CI, 1.2, 1.8] (Table 2, Tables S3 and S4, Figure 2, Figure 3, Figures S1 and S2).

Across BRICS-Plus countries, males had 1.5-fold and 5.3-fold higher CVD ASMR than females attributable to dietary risks and smoking, respectively, in 2019. The increase in CVD ASMR attributable to high BMI was more striking in males (40.8%) than in females (14.4%) during the study period. Overall, males showed less improvement in CVD ASMR reduction than in females attributable to dietary risks (AAPC −0.9% [95% CI, −1.0, −0.8] verses (AAPC −1.1% [95% CI, −1.2, −1.0] and smoking (AAPC −1.2% [95% CI, −1.4, −1.1] verses (AAPC −2.1% [95% CI, −2.2, −2.0], respectively, (Table 3, Tables S5 and S6, Figure 2 and Figures S3–S8).

### 3.3. Age-Specific CVD Burden Attributable to Modifiable Risk Factors

Tables S7–S15 and Figures S9–S18 show the trend of CVD, IHD, and IS burden with age groups (i.e., 25–49 years, 50–69 years, and 70–89 years) attributable to dietary risks, high BMI, and smoking across the BRICS-Plus countries. The trend of diet and smoking-related CVD, IHD, and IS deaths significantly decreased and high BMI-related deaths markedly increased in the BRICS-Plus countries across all age groups. 

Among all BRICS-plus countries, in the younger population (25–49 years), the highest increasing trends of CVD burden attributable to dietary risks (AAPC 3.8% [95% CI, 3.4, 4.3], high BMI (AAPC 6.2% [95% CI, 5.2, 7.1], and smoking (AAPC 3.5% [95% CI, 3.0, 3.9] were observed in the Philippines. Lesotho and Pakistan had a significantly increasing trend of CVD deaths among the younger population attributable to all aforementioned modifiable risk factors during the study period.

### 3.4. Age-Period-Cohort Effects on CVD Mortality across the BRICS-Plus Countries

**Figure 4:**
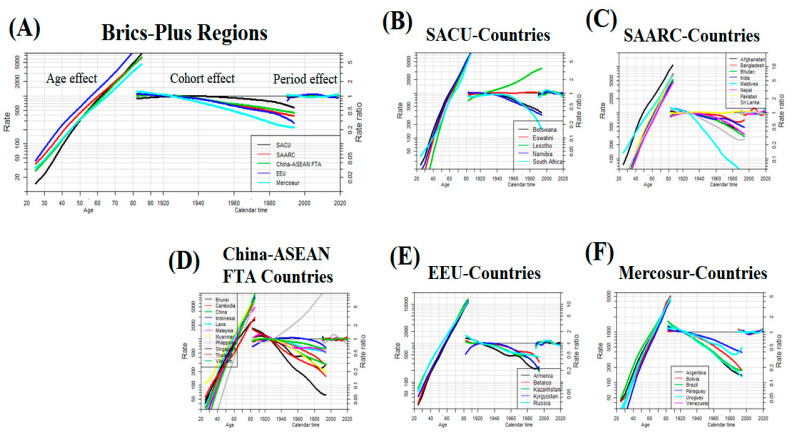
Age-period-cohort effect on cardiovascular disease (CVD) mortality rate per 100,000 populations across BRICS-Plus regions (**A**) (i.e., South African Customs Union (SACU), South Asian Association for Regional Cooperation (SAARC), China-ASEAN Free Trade Area (China-ASEAN FTA), Eurasian Economic Union (EEU), and Mercosur), age-period-cohort effect on CVD mortality rate per 100,000 populations across SACU-Countries (**B**), age-period-cohort effect on CVD mortality rate per 100,000 populations across SAARC-Countries (**C**), age-period-cohort effect on CVD mortality rate per 100,000 populations across China-ASEAN FTA -Countries (**D**), age-period-cohort effect on CVD mortality rate per 100,000 populations across EEU-Countries (**E**), age-period-cohort effect on CVD mortality rate per 100,000 populations across Mercosur-Countries (**F**).

The risk of CVD deaths markedly increased with age across all BRICS-Plus regions and the risk of CVD mortality tends to increase most remarkably in the EEU region with the highest in Russia. The period effect remained consistent during the study period in all BRICS-Plus regions except for EEU and Mercosur regions where moderate fluctuations were observed in CVD deaths from 1990 to 2019. In each of the BRICS-Plus regions, the analysis revealed an increased mortality risk trend in early birth cohorts compared with the median birth cohorts. However, the risk of CVD deaths in the recent birth cohort was the highest in Lesotho in the SACU region and the Philippines in the China-ASEAN FTA region compared with the reference cohort (Figure 4 and Figure S19).

### 3.5. Impact of Sociodemographic Transitions on CVD Mortality across the BRICS-Plus Countries

**Figure 5:**
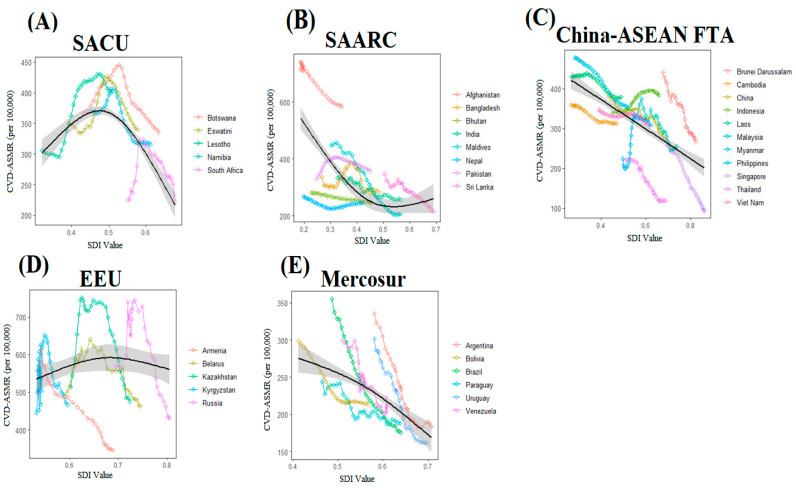
Relationship between countries’ socio-demographic index (SDI) and cardiovascular disease (CVD)_ASMR (age-standardized mortality rate) from 1990–2019 across BRICS-Plus regions (**A**–**E**), (i.e., South African Customs Union (SACU), South Asian Association for Regional Cooperation (SAARC), China-ASEAN Free Trade Area (China-ASEAN FTA), Eurasian Economic Union (EEU), and Mercosur). Association between SACU-region’ SDI and CVD_ASMR from 1990–2019 (**A**), association between SAARC-region’ SDI and CVD_ASMR from 1990–2019 (**B**), association between China-ASEAN FTA-region’ SDI and CVD_ASMR from 1990–2019 (**C**), association between EEU-region’ SDI and CVD_ASMR from 1990–2019 (**D**), association between Mercosur-region’ SDI and CVD_ASMR from 1990–2019 (**E**). Each colored line represents a time trend of the relationship for the specified BRICS-Plus countries. Each point represents a specific year for that country. The black line with 95% confidence band represents the average expected relationship between SDI and ASMR for CVD based on values from all countries from 1990 to 2019. SDI ranges from 0 (less developed) to 1 (most developed).

We found that CVD ASMR declined with the increase of each BRICS-plus country’s SDI but these mortality rates significantly fluctuated during the entire study period. A finite cubic spline with three nodes was used to flexibly simulate the relationship between CVD ASMR and SDI. The estimated relationship between SDI and CVD ASMR is shown as the black line in Figure 5. Countries in the SACU region have shown a quadratic curve for CVD ASMR with the increase in SDI, with a more rapid decline at the highest levels of SDI. Countries in the China-ASEAN FTA, EEU, and Mercosur regions have shown a gradual decline trend of CVD ASMR with the increase in SDI values, specifically countries with a lower level of SDI (i.e., Afghanistan) shown high CVD ASMR than countries with a high level of SDI (i.e., Singapore). The reverse trend in CVD ASMR was found across countries in the SAARC region, decreasing first and then increasing, but this trend was not obvious (Figure 5, Figures S20 and S21). 

## 4. Discussion

Our systematic analysis of Global Burden of Disease 2019 data provides a comprehensive picture of epidemiological trends of CVD burden attributable to modifiable risk factors and its association with sociodemographic transition across BRICS-Plus at regional and country levels. Between 1990 and 2019, all-ages CVD mortality nearly doubled across BRICS-Plus countries. The CVD age-standardized mortality rate attributable to dietary risks and smoking significantly decreased across several BRICS-Plus countries. However, four-fifths of BRICS-Plus countries observed a remarkable increasing trend of BMI-related CVD deaths in particular among younger adults (25–49 years) and ≈3-fold higher CVD mortality in males than in females. In age-period-cohort analysis, the risk of CVD deaths increases with age and the age effect on CVD mortality was more pronounced among individuals aged greater than 50 years. Higher CVD mortality risks were observed in the early birth cohorts compared with the median birth cohorts. Moreover, the age-standardized mortality rate of CVD decreased with increasing socio-demographic index across BRICS-Plus countries.

### 4.1. Overall CVD Mortality

CVDs are common and have been considered a growing epidemic problem worldwide [3]. Across the BRICS-plus countries, the all-ages CVD mortality nearly doubled from 6.1 million in 1990 to 11.3 million in 2019, accounting for 60.9% of global CVD deaths. Although, the age-standardized mortality rate (ASMR) of CVD significantly declined from 367 to 296 per 100,000 in the study period but still higher than the global CVD ASMR of 239 per 100,000 in 2019 [1]. The increase in all-ages CVD mortality and the reduction in ASMR of CVD across BRICS-Plus countries suggest that population growth and aging play a key role in the overall increase in CVD mortality [1]. 

At the regional level, the highest ASMR and the lowest ASMR for CVD were observed in the Eurasian Economic Union (EEU) and Mercosur region, respectively. At the country level, the ASMR for CVD were highest in Afghanistan and were lowest (6-fold) in Singapore in 2019. Lesotho, Pakistan, Indonesia, and the Philippines were the only BRICS-Plus countries with an increase in ASMR for CVD. Singapore, Maldives, and Brazil had a pronounced decline in ASMR for CVD from 1990 to 2019. These striking differences at the regional and country level in total CVD rates and temporal trends could be attributed to differences in access to effective primary and secondary prevention strategies, health care services, and prevalence of CVD risk factors [16,17]. 

### 4.2. CVD Mortality Attributable to Modifiable Risk Factors

Modifiable risk factors are major drivers of CVD burden [1]. The highest ASMR of CVD is attributable to dietary risk followed by high BMI and smoking across BIRCS-plus countries in 2019. The ASMR for CVD attributable to high BMI increased and the CVD ASMR due to smoking remarkably decreased during the study period, indicating an improvement in smoking-related CVD mortality. Dietary risks play a vital role in CVDs and the absolute CVD burden due to dietary risk increased over the last 30 years worldwide. Many countries of the world have limited access to whole grains, healthy fresh fruits, and vegetables and adopted policies to reduce consumption of harmful fats, added sugar, and sodium, but these policies have generally been poorly implemented in low- and middle-income countries and the overall impact on global health is limited [1]. Both high BMI and smoking have a linear association with CVDs and adversely affect cardiovascular metabolic risk factors [1,18,19]. 

Among the 35 BRICS-Plus countries, Afghanistan had the highest diet and high BMI-related CVD ASMR, and Belarus had the highest smoking-related CVD ASMR in 2019. The Philippines observed a significant increase in CVD ASMR attributable to the aforementioned modifiable risk factors over the study period. However, Singapore had the lowest age-standardized CVD mortality rate and substantial reduction in CVD ASMR attributable to dietary risks, high BMI, and smoking from 1990 to 2019. The differences in CVD ASMR attributable to modifiable risk factors among BRICS-Plus countries reflect variations in diet quality and levels of exposure to different dietary risks and differences in implementing obesity and smoking-related prevention strategies [1]. 

Across BRICS-Plus countries, males had 1.5-fold and 5.3-fold higher CVD ASMR than females in 2019 and showed less improvement in CVD ASMR reduction attributable to dietary risks and smoking, respectively. The increase in CVD ASMR attributable to high BMI was ≈3-fold higher in males than in females during the study period. Globally, men are less adhere to healthy dietary patterns and had lower healthy eating index scores than women [20]. In 2019, the prevalence of smoking was higher in males (33.5%) than in females (6.8%) worldwide [1]. As a result of these differences, higher CVD mortality attributable to modifiable risk factors occurred among men. 

Our findings showed that adults aged 25–49 years showed a pronounced increasing trend of BMI-related and slower trend of diet and smoking-related CVD, IHD, and IS deaths compared with the old age group (50–69 years). The marked upward trend of BMI-related CVD deaths among the younger population is alarming and should need proper attention and primary prevention strategies for weight gain across the BRICS-plus countries. A sedentary lifestyle could be a significant contributor to CVD premature mortality among younger adults as sedentary lifestyles steadily increased for both sexes at the age of 25 years worldwide [1]. Moreover, the low intake of whole grains among young adults (25–50 years) is the leading risk factor for deaths and disability-adjusted life years [21]. 

We observed that four-fifths of BRICS-Plus countries observed a remarkable increasing trend of BMI-related ASMR of CVD in particular among younger adults. It suggests that a multifactorial approach and interventions are urgently required to improve diet quality, promote exercise and physical activity, and reduce sedentary behavior, particularly among young adults. Governmental-level interventions are needed to regulate food ingredients and control the advertisement of unhealthy diets as observed in the North Karelia Project in Finland [22]. The World Health Organization’s Framework Convention on Tobacco Control measures should be seriously adopted to reduce the smoking-related CVD burden in the BRICS-Plus countries. Controlling modifiable risk factors reduced more than 50% CVD mortality and could prevent or delay cardiometabolic risk factors [23,24]. Moreover, young adults should undertake moderate-intensity (2.5–5.0 h) or vigorous-intensity physical activity (1.25–2.5 h) per week to prevent and reduce cardiovascular disease morbidity and mortality [25,26].

### 4.3. Age-Period-Cohort Effects on CVD Mortality across the BRICS-Plus Countries

Age effect indicates differences in CVD rates across different age groups, cohort effect shows differences in risks and lifestyles across birth cohorts, and period effect reflects variation in the CVD rates over time that influence all ages and cohorts simultaneously. In age-period-cohort analysis, period and cohort effects assist in determining the success of earlier health-policy interventions and identifying future targets [27,28]. We observed that the risk of CVD deaths increases with age and the age effect on CVD mortality was more pronounced among individuals aged greater than 50 years across all BRICS-Plus countries. Moreover, the risk of CVD mortality tends to increase with age most remarkably in the EEU region with the highest in Russia. Age is an independent risk factor of CVD in old people and the proportion of CVD incidence linearly increased with aging among both men and women [29,30]. The highest risk of CVD mortality in the EEU region and Russia could be attributed to poor diet quality, higher prevalence of smoking and alcohol use, and high-level use of illicit drugs [20,31,32]. 

The period effect remained consistent during the study period in all BRICS-Plus regions except for EEU and Mercosur regions where moderate fluctuations were observed in CVD deaths from 1990 to 2019. Mercosur region showed a gradual slope reduction in CVD deaths over the study period. However, in the EEU region, an increasing trend of CVD deaths was observed in the early two decades (1990–2010) and a gradual reduction in the last decade (2010–2019). The Soviet Union’s collapse since the 1990s has significantly influenced population health in the region through conflicts, economic crises, and more porous borders that contributed to increasing non-communicable diseases. Moreover, the obsolete inherited healthcare system was unable to cope with existing public health problems and remained underfunded through a prolonged healthcare reform [33,34]. A gradual reduction in CVD deaths across the EEU region in the last decade could be attributed to the implementation of several nationwide prevention programs for cardiovascular diseases in Russia, Belarus, and Kazakhstan [35,36].

We observed an increase in CVD mortality risk in the early birth cohorts compared with the median birth cohorts across all BRICS-Plus regions. However, the risk of CVD deaths in the recent birth cohort was the highest in Lesotho in the SACU region and the Philippines in the China-ASEAN FTA region. The highest risk of CVD deaths in recent birth cohorts in Lesotho could be due to an extremely poor healthcare system, inadequate and unequal health resources, and service delivery. The United Nations declared Lesotho as one of the most underdeveloped countries with a higher poverty rate and increasing NCD burden [37]. Moreover, young adults in Lesotho had a higher prevalence of smoking, high-level use of alcohol, poor diet quality, and low physical activity [38]. Similarly, factors contributing to the highest risk of CVD mortality in young cohorts in the Philippines included poor dietary habits [20,39], higher prevalence of smoking [40], and high BMI [1] as shown in our findings.

### 4.4. Sociodemographic Transitions and CVD Mortality across the BRICS-Plus Countries

The socio-demographic index (SDI), a composite indicator of development status, has been used as an effective measure for comparing regional and national disease burdens and evaluating the effectiveness of health policies in countries with socioeconomic transitions [41]. We showed that CVD ASMR decreased with increasing SDI across BRICS-Plus countries. In general, countries with a lower level of SDI had higher CVD ASMR compared with a high level of SDI countries. Afghanistan with the lowest SDI score had a six-fold higher CVD ASMR than Singapore with the highest SDI score among BRICS-Plus countries in 2019. Across the BRICS-Plus regions, China-ASEAN FTA and Mercosur regions showed a gradual decline trend of CVD ASMR with the increase in SDI values. The socio-demographic transition was associated with a remarkable CVD decline in high-and-middle income countries but only reflects a gradual decline or no change in several low- and middle-income countries in the last three decades [42,43]. Globally, low- and middle-income countries experienced ~3 times higher CVD deaths than high-income countries [43]. Moreover, regions with high SDI showed ~4-fold CVD death reduction than regions with low SDI [44]. Notably, these findings suggest that changes in CVD deaths are strongly related to a country or region’s SDI and should increase investment in the primary and secondary prevention of CVD in countries and regions with low SDI. 

### 4.5. Limitations

Our study has certain limitations. First, our analysis is based on GBD secondary data and all GBD limitations are applicable to our findings as mentioned previously [21]. Second, we showed the trend of CVD deaths attributable to overall dietary risks and could not find the trend for the individual dietary risk factor. Third, CVD deaths attributable to modifiable risk factors were investigated among only three age groups and data for those <25 years of age were not included in the age-period-cohort analysis. Fourth, age-period-cohort analysis and SDI association were only conducted for cause-specific CVD deaths and not for modifiable risk factors-related CVD mortality. 

## 5. Conclusions and Implications

The age-standardized mortality rate of CVD significantly declined; however, the all-ages CVD deaths nearly doubled across the BRICS-Plus countries during the study period. The highest age-standardized mortality rate of CVD is attributable to dietary risk across BIRCS-plus countries in 2019. The CVD age-standardized mortality rate attributable to dietary risks and smoking significantly decreased across many BRICS-Plus countries. However, four-fifths of BRICS-Plus countries observed a remarkable increasing trend of BMI-related CVD deaths in particular among younger adults (25–49 years) and ≈3-fold higher CVD mortality in males than in females. The risk of CVD mortality was more pronounced among individuals aged greater than 50 years and in the early birth cohorts. The age-standardized mortality rate of CVD decreased with increasing socio-demographic index across BRICS-Plus countries. These epidemiological trends and patterns of CVD mortality may provide references to health policymakers and decision-makers to allocate their healthcare resources and specify prevention and control strategies for modifiable risk factors at regional and country levels across BRICS-Plus.

## Figures and Tables

**Table 1:** The average annual percent change in the burden of cause-specific CVD mortality for both sexes across BRICS-Plus countries from 1990 to 2019.

CVD	ASMR/100,000			Deaths, n × 1000		
	1990 (95%UI)	2019 (95%UI)	AAPC (95%CI)	1990 (95%UI)	2019 (95%UI)	AAPC (95%CI)
**BRICS-Plus countries**	367 (407, 326)	296 (346, 248)	−0.7 (−0.8, −0.6)	6197 (6798, 5610)	11,310 (12896, 9704)	2.1 (1.9, 2.3)
**SACU**						
Botswana	366 (448, 302)	334 (425, 258)	−0.3 (−0.4, −0.2)	1.6 (2.1, 1.3)	3.5 (4.5, 2.6)	2.6 (2.4, 2.7)
Eswatini	350 (405, 293)	339 (441, 258)	−0.1 (−0.4, 0.2)	0.8 (0.9, 0.6)	1.5 (2.1, 1.1)	2.0 (1.8, 2.3)
Lesotho	305 (354, 261)	399 (497, 311)	0.9 (0.8, 1.0)	2.5 (2.9, 2.1)	4.1 (5.1, 3.1)	1.6 (1.4, 1.7)
Namibia	370 (425, 313)	316 (381, 263)	−0.5 (−0.7, −0.4)	2.2 (2.5, 1.8)	3.7 (4.5, 3.1)	1.8 (1.6, 2.0)
South Africa	225 (244, 202)	222 (236, 203)	−0.1 (−0.6, 0.6)	42 (46, 38)	82 (87, 76)	2.3 (1.7, 2.9)
**SAARC**						
Afghanistan	716 (829, 608)	583 (681, 473)	−0.7 (−0.8, −0.6)	44 (52, 37)	61 (75, 49)	1.1 (1.0, 1.2)
Bangladesh	335 (374, 290)	283 (341, 220)	−0.4 (−0.9, 0.1)	138 (154, 119)	324 (394, 250)	3.2 (2.8, 3.7)
Bhutan	280 (343, 220)	246 (286, 198)	−0.5 (−0.5, −0.4)	0.5 (0.7, 0.4)	1.2 (1.4, 0.9)	2.6 (2.5, 2.7)
India	332 (371, 297)	256 (292, 220)	−1.0 (−1.6, −0.3)	1201 (1328, 1080)	2574 (2940, 2215)	2.7 (1.9, 3.4)
Maldives	446 (481, 414)	205 (239, 174)	−2.7 (−3.0, −2.4)	0.3 (0.3, 0.2)	0.5 (0.6, 0.4)	1.6 (1.4, 1.9)
Nepal	267 (332, 218)	245 (286, 199)	−0.3 (−0.4, −0.2)	21 (26, 17)	46 (54, 37)	2.7 (2.5, 2.8)
Pakistan	329 (381, 279)	357 (423, 307)	0.3 (0.3, 0.4)	172 (197, 147)	341 (405, 291)	2.4 (2.3, 2.4)
Sri Lanka	346 (372, 318)	215 (270, 163)	−1.6 (−2.0, −1.3)	30 (32, 27)	46 (59, 35)	1.5 (1.2, 1.9)
**China-ASEAN FTA**						
Brunei Darussalam	441 (475, 404)	266 (296, 240)	−1.7 (−2.2, −1.2)	0.3 (0.3, 0.2)	0.5 (0.5, 0.4)	1.6 (1.4, 1.8)
Cambodia	359 (412, 313)	312 (349, 265)	−0.5 (−0.5, −0.4)	13 (15, 12)	30 (34, 25)	2.7 (2.6, 2.8)
China	381 (426, 341)	276 (312, 239)	−1.1 (−1.4, −0.8)	2423 (2712, 2165)	4584 (5209, 3955)	2.2 (1.9, 2.6)
Indonesia	340 (375, 301)	383 (418, 328)	0.4 (0.4, 0.5)	278 (304, 250)	651 (721, 554)	3.0 (2.9, 3.0)
Laos	428 (517, 348)	379 (440, 318)	−0.4 (−0.5, −0.4)	7.5 (9.1, 6.1)	13 (16, 11)	2.0 (2.0, 2.1)
Malaysia	347 (363, 326)	255 (309, 207)	−1.0 (−1.7, −0.3)	28 (29, 26)	58 (71, 47)	2.6 (1.7, 3.4)
Myanmar	477 (570, 400)	352 (401, 313)	−1.1 (−1.1, −1.0)	96 (116, 79)	138 (159, 120)	1.2 (1.1, 1.3)
Philippines	223 (246, 200)	307 (356, 254)	1.3 (0.8, 1.7)	45 (53, 41)	204 (241, 165)	5.5 (5.1, 5.8)
Singapore	271 (280, 254)	93 (100, 81)	−3.6 (−3.9, −3.3)	5.1 (5.2, 4.8)	6.8 (7.3, 6.1)	1.1 (0.9, 1.3)
Thailand	219 (244, 196)	118 (150, 90)	−2.1 (−2.5, −1.7)	63 (71, 57)	115 (147, 88)	2.0 (1.7, 2.4)
Viet Nam	346 (405, 293)	303 (348, 258)	−0.4 (−0.5, −0.4)	124 (145, 104)	240 (278, 202)	2.3 (2.3, 2.4)
**EEU**						
Armenia	493 (510, 462)	345 (396, 295)	−1.3 (−1.7, −0.8)	10.6 (10.9, 10.1)	13 (15, 11)	0.7 (0.3, 1.1)
Belarus	489 (506, 459)	463 (571, 381)	−0.2 (−0.7, 0.3)	59 (61, 56)	75 (92, 62)	0.8 (0.3, 1.3)
Kazakhstan	513 (534, 485)	476 (531, 421)	−0.3 (−0.6, 0.1)	56 (58, 53)	65 (73, 57)	0.4 (−0.1, 0.8)
Kyrgyzstan	445 (470, 415)	466 (517, 416)	0.2 (−0.4, 0.8)	12 (13, 12)	17 (19, 15)	1.1 (0.3, 1.8)
Russia	569 (584, 540)	432 (485, 378)	−1.0 (−2.0, 0.1)	890 (908, 856)	1004 (1126, 880)	0.4 (−0.6, 1.4)
**Mercosur**						
Argentina	335 (347, 315)	183 (194, 168)	−2.0 (−2.5, −1.5)	98 (101, 93)	101 (107, 93)	0.1 (−0.4, 0.6)
Bolivia	298 (354, 244)	214 (265, 161)	−1.1 (−1.2, −1.0)	8.2 (9.8, 6.6)	15 (19, 11)	2.3 (2.2, 2.3)
Brazil	355 (367, 332)	175 (184, 158)	−2.4 (−2.6, −2.2)	269 (277, 257)	397 (417, 361)	1.4 (1.1, 1.6)
Paraguay	244 (269, 211)	187 (235, 147)	−0.8 (−1.4, −0.1)	5.1 (5.5, 4.3)	9.9 (12.4, 7.8)	2.4 (1.8, 3.1)
Uruguay	302 (313, 282)	161 (170, 146)	−2.1 (−2.5, −1.7)	11 (11, 10)	10 (10, 8)	−0.4 (−0.8, −0.1)
Venezuela	299 (312, 279)	222 (279, 176)	−1.0 (−1.9, −0.1)	25 (26, 24)	61 (77, 48)	3.0 (2.1, 3.9)

Note: AAPC, average annual percent change; ASMR, age-standardized mortality rate; CVD, cardiovascular diseases; BRICS, Brazil, Russia, India, China, and South Africa; SACU, South African Customs Union; SAARC, South Asian Association for Regional Cooperation; China-ASEAN FTA, China-ASEAN Free Trade Area and EEU, Eurasian Economic Union.

**Table 2:** The average annual percent change in the burden of CVD mortality for both sexes attributable to modifiable risk factors across BRICS-Plus countries from 1990 to 2019.

Population	CVD (ASMR/100,000)		
	Dietary Risks (AAPC (95%CI)	High BMI (AAPC (95%CI)	Smoking (AAPC (95%CI)
**BRICS-Plus countries**	−1.0 (−1.1, −0.9)	0.8 (0.7, 1.0)	−1.4 (−1.5, −1.3)
**SACU**			
Botswana	−0.2 (−0.3, −0.1)	2.1 (1.8, 2.5)	−0.9 (−1.0, −0.8)
Eswatini	−0.3 (−0.6, −0.1)	0.6 (0.1, 1.1)	−1.6 (−1.8, −1.3)
Lesotho	0.9 (0.8, 1.0)	3.1 (2.9, 3.3)	1.0 (0.8, 1.1)
Namibia	−0.7 (−0.8, −0.6)	0.6 (0.4, 0.8)	−1.8 (−2.0, −1.7)
South Africa	−0.3 (−0.9, 0.4)	0.4 (−0.2, 1.1)	−2.5 (−3.2, −1.8)
**SAARC**			
Afghanistan	−0.9 (−0.9, −0.8)	0.8 (0.5, 1.0)	0.4 (0.3, 0.5)
Bangladesh	−0.4 (−0.9, 0.1)	3.2 (2.8, 3.7)	−1.4 (−1.8, −1.0)
Bhutan	−0.4 (−0.5, −0.3)	1.9 (1.8, 2.0)	−1.1 (−1.3, −1.0)
India	−0.8 (−1.6, −0.1)	2.4 (2.0, 2.7)	−1.4 (−2.0, −0.8)
Maldives	−3.2 (−3.5, −2.9)	−0.3 (−0.5, −0.1)	−3.8 (−4.1, −3.5)
Nepal	−0.2 (−0.4, −0.1)	3.4 (3.3, 3.5)	−0.9 (−1.0, −0.8)
Pakistan	0.7 (0.6, 0.7)	3.1 (2.9, 3.2)	−0.4 (−0.5, −0.3)
Sri Lanka	−2.1 (−2.5, −1.6)	0.7 (0.3, 1.0)	−3.8 (−4.3, −3.3)
**China-ASEAN FTA**			
Brunei Darussalam	−1.9 (−2.4, −1.5)	−0.1 (−0.3, 0.1)	−3.6 (−3.9, −3.3)
Cambodia	−0.9 (−1.0, −0.9)	2.1 (2.0, 2.2)	−0.8 (−0.8, −0.7)
China	−1.2 (−1.5, −0.9)	0.9 (0.4, 1.4)	−0.7 (−1.2, −0.3)
Indonesia	−0.1 (−0.2, −0.1)	3.2 (3.2, 3.3)	0.7 (0.7, 0.8)
Laos	−1.1 (−1.2, −1.0)	2.8 (2.7, 2.9)	−1.3 (−1.4, −1.2)
Malaysia	−1.3 (−2.0, −0.7)	0.6 (0.2, 1.0)	−1.5 (−2.2, −0.7)
Myanmar	−1.8 (−1.8, −1.7)	2.2 (2.0, 2.3)	−3.2 (−3.3, −3.1)
Philippines	1.1 (0.7, 1.6)	4.3 (3.5, 5.1)	1.5 (1.2, 1.8)
Singapore	−4.1 (−4.3, −3.8)	−1.6 (−1.9, −1.4)	−5.3 (−5.6, −5.0)
Thailand	−2.6 (−2.9, −2.2)	0.9 (0.5, 1.3)	−3.4 (−4.2, −2.6)
Viet Nam	−1.0 (−1.0, −0.9)	3.3 (3.1, 3.5)	−0.3 (−0.3, −0.2)
**EEU**			
Armenia	−1.5 (−1.8, −1.2)	0.3 (−0.1, 0.6)	−1.4 (−1.7, −1.0)
Belarus	−0.3 (−0.9, 0.3)	0.6 (0.1, 1.2)	−0.1 (−1.2, 1.0)
Kazakhstan	−0.9 (−1.2, −0.6)	0.1 (−0.3, 0.4)	−1.0 (−1.4, −0.5)
Kyrgyzstan	0.3 (−0.4, 0.9)	0.3 (−0.3, 0.9)	−0.2 (−0.7, 0.2)
Russia	−1.3 (−2.4, −0.2)	0.1 (−1.0, 1.0)	−0.1 (−1.2, 1.1)
**Mercosur**			
Argentina	−2.6 (−2.9, −2.3)	−0.9 (−1.4, −0.5)	−3.0 (−3.3, −2.8)
Bolivia	−1.2 (−1.4, −1.1)	0.2 (0.1, 0.3)	−3.4 (−3.5, −3.3)
Brazil	−2.9 (−3.1, −2.7)	−1.1 (−1.4, −0.8)	−4.1 (−4.3, −3.8)
Paraguay	−1.1 (−1.8, −0.4)	0.1 (−0.8, 0.9)	−1.7 (−2.4, −1.1)
Uruguay	−2.8 (−3.2, −2.3)	−1.0 (−1.4, −0.7)	−2.6 (−3.1, −2.0)
Venezuela	−1.2 (−1.7, −0.6)	−0.4 (−1.0, 0.1)	−3.0 (−3.6, −2.4)

Note: AAPC, average annual percent change; ASMR, age-standardized mortality rate; CVD, cardiovascular diseases; BRICS, Brazil, Russia, India, China, and South Africa; SACU, South African Customs Union; SAARC, South Asian Association for Regional Cooperation; China-ASEAN FTA, China-ASEAN Free Trade Area and EEU, Eurasian Economic Union.

**Table 3:** The average annual percent change in the burden of CVD ASMR attributable to modifiable risk factors in males and females across BRICS-Plus countries from 1990 to 2019.

Population	Male (AAPC (95%CI)			Female (AAPC (95%CI)		
	Dietary Risks	High BMI	Smoking	Dietary Risks	High BMI	Smoking
**BRICS-Plus countries**	−0.9 (−1.0, −0.8)	1.2 (1.0,1.4)	−1.2 (−1.4, −1.1)	−1.1 (−1.2, −1.0)	0.5 (0.3, 0.6)	−2.1 (−2.2, −2.0)
**SACU**						
Botswana	−0.2 (−0.4, 0.1)	3.2 (2.9, 3.5)	−0.7 (−1.0, −0.5)	−0.2 (−0.8, 0.3)	1.6 (0.8, 2.6)	−1.1 (−1.9, −0.2)
Eswatini	−0.2 (−0.4, −0.1)	1.3 (0.9, 1.7)	−1.5 (−1.6, −1.3)	−0.4 (−0.7, −0.1)	−0.1 (−0.5, 0.4)	−1.4 (−1.6, −1.2)
Lesotho	0.7 (0.6, 0.8)	3.7 (3.4, 4.1)	1.1 (0.8, 1.3)	1.0 (0.8, 1.3)	2.6 (2.3, 2.9)	1.0 (0.7, 1.3)
Namibia	−0.4 (−0.5, −0.2)	1.4 (1.2, 1.6)	−1.6 (−1.7, −1.4)	−1.0 (−1.1, −0.8)	0.1 (−0.2, 0.2)	−2.1 (−2.3, −1.9)
South Africa	−0.3 (−0.8, 0.1)	1.1 (0.5, 1.6)	−2.2 (−2.8, −1.5)	−0.3 (−1.0, 0.4)	0.1 (−0.7, 0.8)	−3.1 (−3.9, −2.3)
**SAARC**						
Afghanistan	−1.0 (−1.1, −0.9)	1.1 (0.9, 1.3)	0.4 (0.3, 0.5)	−0.7 (−0.8, −0.6)	0.5 (0.2, 0.8)	0.8 (0.6, 1.0)
Bangladesh	−0.2 (−0.7, 0.3)	3.9 (3.5, 4.3)	−1.2 (−1.6, −0.7)	−0.7 (−1.4, −0.1)	2.8 (2.4, 3.3)	−1.9 (−2.4, −1.5)
Bhutan	−0.2 (−0.3, −0.1)	2.5 (2.4, 2.7)	−1.1 (−1.2, −1.0)	−0.7 (−0.8, −0.7)	1.4 (1.3, 1.5)	−1.8 (−2.0, −1.7)
India	−0.7 (−1.4, −0.1)	2.6 (2.1, 3.0)	−1.3 (−1.9, −0.6)	−0.8 (−1.7, 0.1)	2.1 (1.7, 2.5)	−1.0 (−1.7, −0.3)
Maldives	−2.8 (−3.1, −2.6)	0.4 (0.2, 0.6)	−3.4 (−3.6, −3.1)	−3.7 (−4.3, −3.0)	−1.3 (−2.0, −0.6)	−5.2 (−6.0, −4.3)
Nepal	0.3 (0.2, 0.4)	4.2 (4.1, 4.3)	−0.3 (−0.5, −0.2)	−1.0 (−1.1, −0.9)	2.6 (2.4, 2.7)	−1.7 (−1.8, −1.7)
Pakistan	1.0 (0.9, 1.1)	3.5 (3.4, 3.7)	−0.1 (−0.2, 0.1)	0.3 (0.3, 0.4)	2.6 (2.4, 2.7)	−0.9 (−1.0, −0.8)
Sri Lanka	−2.0 (−2.5, −1.5)	0.6 (0.1, 1.1)	−3.4 (−3.9, −2.8)	−1.8 (−2.4, −1.1)	0.7 (0.3, 1.1)	−3.9 (−4.5, −3.3)
**China-ASEAN FTA**						
Brunei Darussalam	−1.7 (−2.6, −0.8)	0.2 (−0.5, 0.8)	−3.4 (−4.1, −2.6)	−1.9 (−2.3, −1.4)	−0.3 (−0.6, −0.1)	−3.8 (−4.1, −3.6)
Cambodia	−0.7 (−0.8, −0.7)	2.8 (2.7, 3.0)	−0.5 (−0.6, −0.5)	−1.1 (−1.2, −1.1)	1.5 (1.4, 1.7)	−1.0 (−1.1, −1.0)
China	−0.8 (−1.1, −0.5)	1.5 (1.3, 1.7)	−0.7 (−1.0, −0.5)	−1.7 (−1.9, −1.4)	0.2 (−0.1, 0.5)	−0.6 (−1.0, −0.2)
Indonesia	0.2 (0.1, 0.2)	3.6 (3.5, 3.7)	0.8 (0.7, 0.9)	−0.4 (−0.5, −0.4)	2.9 (2.8, 3.1)	0.7 (0.5, 0.8)
Laos	−1.1 (−1.2, −1.1)	3.2 (3.1, 3.4)	−1.3 (−1.3, −1.2)	−1.1 (−1.2, −1.0)	2.4 (2.3, 2.5)	−1.9 (−2.0, −1.8)
Malaysia	−1.2 (−2.1, −0.3)	1.0 (0.6, 1.5)	−1.5 (−2.3, −0.7)	−1.5 (−2.2, −0.9)	0.1 (−0.5, 0.5)	−2.3 (−3.1, −1.5)
Myanmar	−1.5 (−1.6, −1.4)	2.3 (2.2, 2.5)	−2.6 (−2.7, −2.5)	−2.0 (−2.0, −1.9)	1.9 (1.8, 2.1)	−4.1 (−4.2, −4.0)
Philippines	1.5 (1.2, 1.7)	4.5 (3.4, 5.6)	1.8 (1.5, 2.1)	0.7 (0.2, 1.1)	4.0 (3.4, 4.7)	0.9 (0.5, 1.3)
Singapore	−3.9 (−4.3, −3.6)	−1.3 (−1.5, −1.1)	−5.3 (−5.5, −5.0)	−4.4 (−4.7, −4.0)	−2.2 (−2.6, −1.8)	−6.2 (−6.5, −5.9)
Thailand	−2.5 (−3.3, −1.7)	1.4 (0.9, 1.9)	−3.2 (−4.0, −2.4)	−2.8 (−3.1, −2.5)	0.3 (−0.1, 0.5)	−5.1 (−5.4, −4.7)
Viet Nam	−0.6 (−0.7, −0.6)	4.1 (3.8, 4.3)	−0.2 (−0.3, −0.2)	−1.5 (−1.5, −1.4)	2.3 (2.2, 2.4)	−1.3 (−1.4, −1.2)
**EEU**						
Armenia	−1.3 (−1.7, −1.0)	1.1 (0.8, 1.3)	−1.4 (−1.9, −0.9)	−1.7 (−2.0, −1.4)	−0.3 (−0.8, 0.1)	−2.5 (−2.9, −2.1)
Belarus	−0.1 (−0.5, 0.4)	1.4 (0.7, 2.0)	−0.1 (−0.7, 0.6)	−0.6 (−1.1, −0.2)	0.1 (−0.3, 0.6)	−0.7 (−1.3, −0.1)
Kazakhstan	−1.0 (−1.3, −0.6)	0.4 (−0.1, 0.8)	−1.1 (−1.6, −0.7)	−0.9 (−1.2, −0.6)	−0.2 (−0.5, 0.1)	−1.2 (−1.6, −0.8)
Kyrgyzstan	0.1 (−0.7, 0.8)	0.7 (0.2, 1.1)	−0.4 (−0.9, 0.1)	0.3 (−0.3, 0.9)	0.1 (−0.6, 0.7)	−0.3 (−0.9, 0.3)
Russia	−1.4 (−2.4, −0.3)	0.4 (−0.7, 1.5)	−0.6 (−1.7, 0.6)	−1.5 (−2.6, −0.4)	−0.4 (−1.4, 0.6)	0.4 (−0.6, 1.4)
**Mercosur**						
Argentina	−2.6 (−2.9, −2.3)	−1.0 (−1.4, −0.6)	−3.1 (−3.4, −2.8)	−2.6 (−3.0, −2.3)	−0.9 (−1.3, −0.6)	−2.9 (−3.2, −2.7)
Bolivia	−1.4 (−1.5, −1.2)	0.4 (0.3, 0.5)	−3.4 (−3.6, −3.3)	−1.2 (−1.4, −1.0)	−0.1 (−0.1, 0.1)	−3.8 (−4.0, −3.7)
Brazil	−2.7 (−2.9, −2.5)	−0.9 (−1.1, −0.7)	−3.9 (−4.2, −3.6)	−3.0 (−3.2, −2.8)	−1.4 (−1.7, −1.1)	−4.2 (−4.5, −3.9)
Paraguay	−0.7 (−1.4, 0.1)	0.6 (−0.3, 1.5)	−1.4 (−2.1, −0.8)	−1.5 (−2.2, −0.8)	−0.5 (−1.3, 0.3)	−2.4 (−3.0, −1.7)
Uruguay	−2.6 (−3.2, −2.0)	−1.0 (−1.4, −0.6)	−2.6 (−2.9, −2.2)	−2.9 (−3.3, −2.4)	−1.1 (−1.5, −0.7)	−2.4 (−2.7, −2.1)
Venezuela	−0.9 (−1.7, −0.1)	−0.1 (−1.3, 1.1)	−2.7 (−3.5, −2.0)	−1.6 (−2.0, −1.2)	−0.9 (−1.4, −0.5)	−3.4 (−3.8, −3.0)

Note: AAPC, average annual percent change; ASMR, age-standardized mortality rate; CVD, cardiovascular diseases; BRICS, Brazil, Russia, India, China, and South Africa; SACU, South African Customs Union; SAARC, South Asian Association for Regional Cooperation; China-ASEAN FTA, China-ASEAN Free Trade Area and EEU, Eurasian Economic Union.

## Data Availability

The original contributions presented in the study are included in the article/Supplementary Materials, further inquiries can be directed to the corresponding author.

## Supplementary Materials

The following supporting information can be downloaded at: https://www.mdpi.com/article/10.3390/nu15173757/s1, Temporal trend of CVD, IHD, and IS burden based on gender and age groups attributable to modifiable risk factors across BRICS-Plus countries from 1990 to 2019 (Figures S1–S18 and Tables S1–S15), Age-period-cohort effect on CVD mortality and its association with SDI across BRICS-Plus countries from 1990 to 2019 (Figures S19–S21).

## Abbreviations

AAPC	Average annual percentage change
ASMR	Age-standardized mortality rate
BMI	Body mass-index
BRICS-Plus	Brazil, Russia, India, China, South Africa, and 30 other countries
China-ASEAN FTA	China-ASEAN Free Trade Area
CVD	Cardiovascular disease
EEU	Eurasian Economic Union
GBD	Global Burden of Disease
IHD	Ischemic heart diseases
IS	Ischemic stroke
ML	Maximum Likelihood
RR	Rate ratio
SAARC	South Asian Association for Regional Cooperation
SACU	South African Customs Union
SDI	Sociodemographic index
