# Supplementary material for: Epidemiological Trends in Cardiovascular Disease Mortality Attributable to Modifiable Risk Factors and Its Association with Sociodemographic Transitions across BRICS-Plus Countries
Source: Nutrients. 2023 Aug 28;15(17):3757. doi: 10.3390/nu15173757 (PMC10489729; doi:10.3390/nu15173757)
Supplement: Supplementary file 1 [file nutrients-15-03757-s001.zip › nutrients-2547518-supplementary.pdf]

Figure S1: The temporal trend of IHD_ASMR (age-standardized mortality rate) for both sexes attributable to dietary risks (A), high BMI (B) and smoking (C) across BRICS-Plus from 1990 to 2019.

A (dietary risks) | B (High BMI) | C (Smoking)

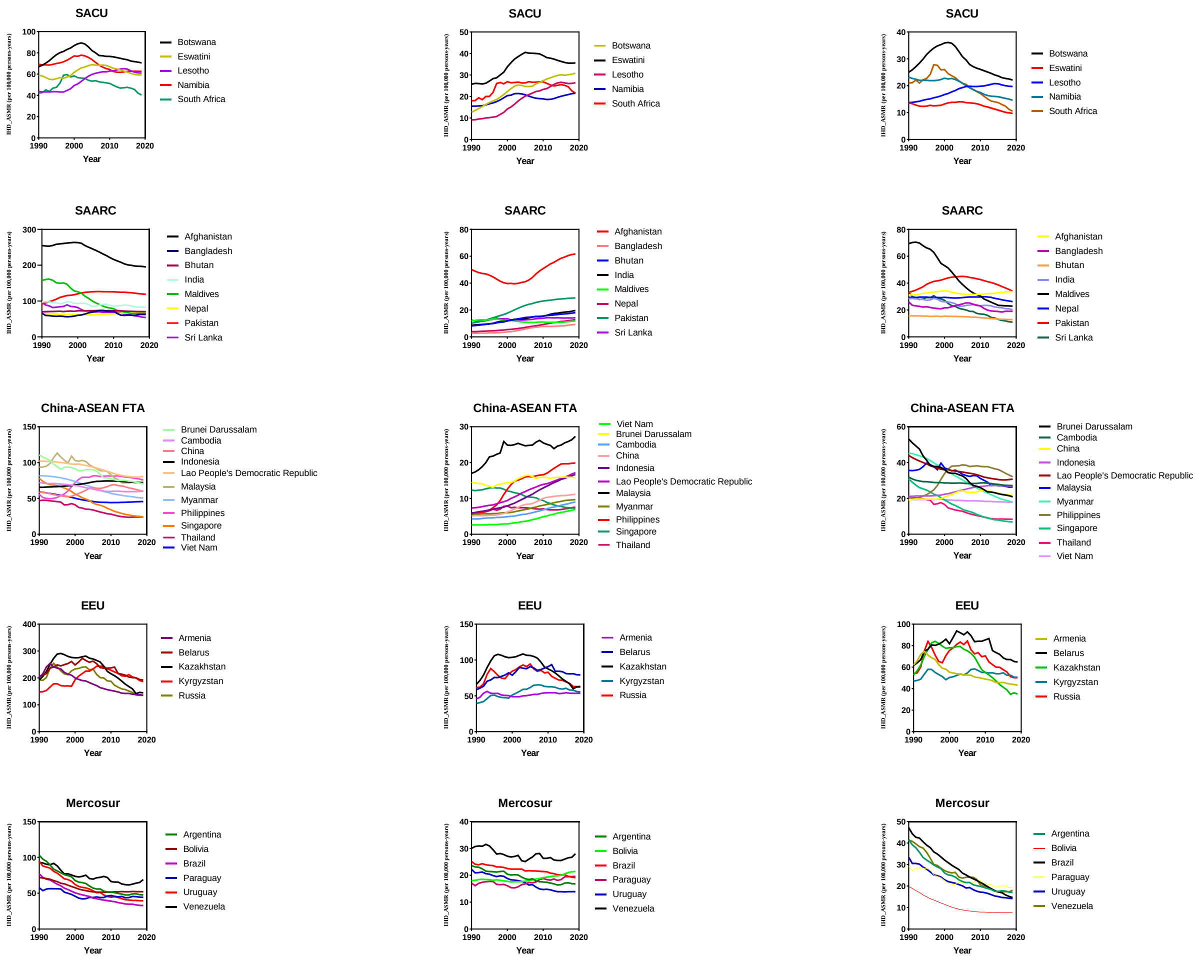

Figure S2: The temporal trend of IS_ASMR (age-standardized mortality rate) for both sexes attributable to dietary risks (A), high BMI (B) and smoking (C) across BRICS-Plus from 1990 to 2019.

A (dietary risks) **B (High BMI)** **C (Smoking)**

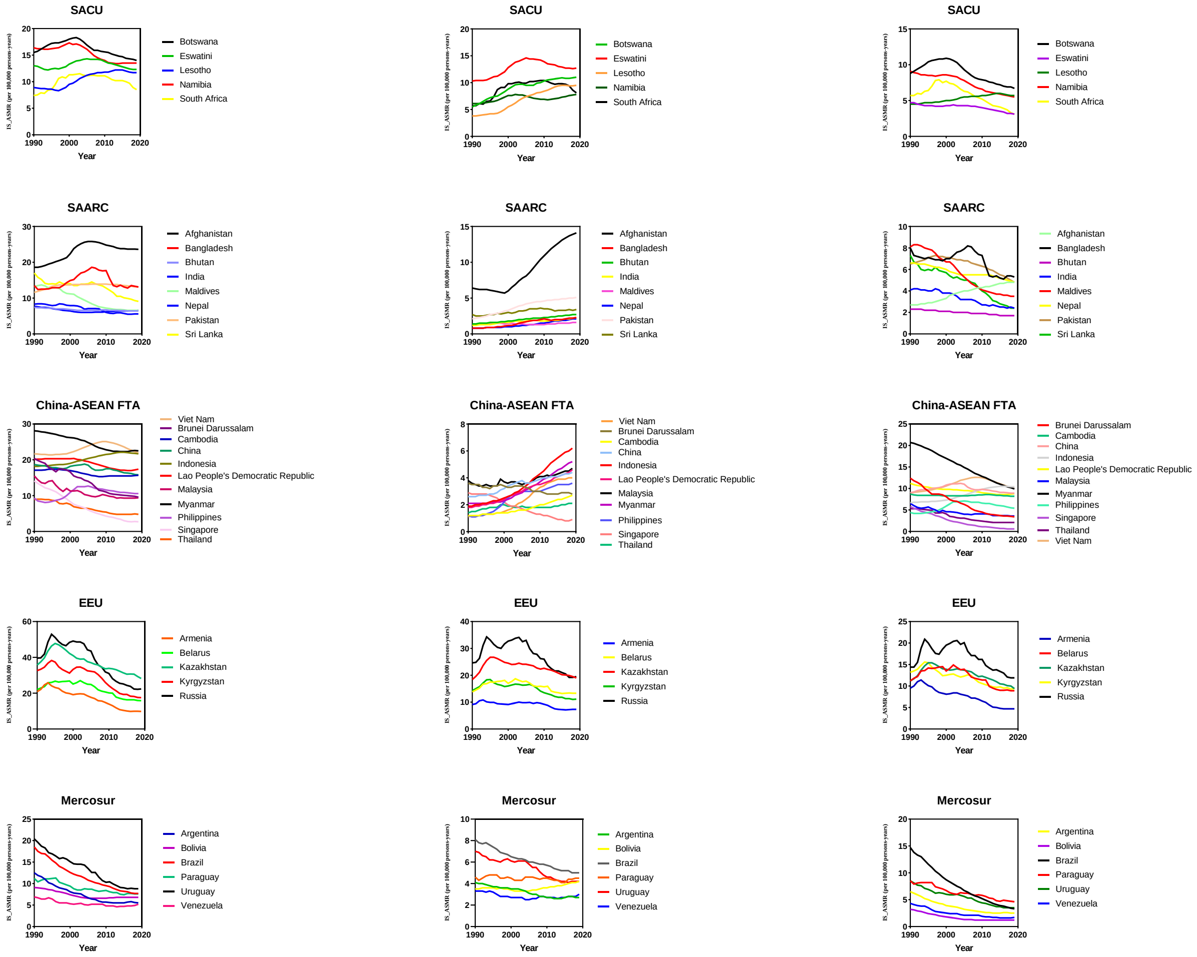

Figure S3: The temporal trend of CVD_ASMR (age-standardized mortality rate) for males attributable to dietary risks (A), high BMI (B) and smoking (C) across BRICS-Plus from 1990 to 2019.

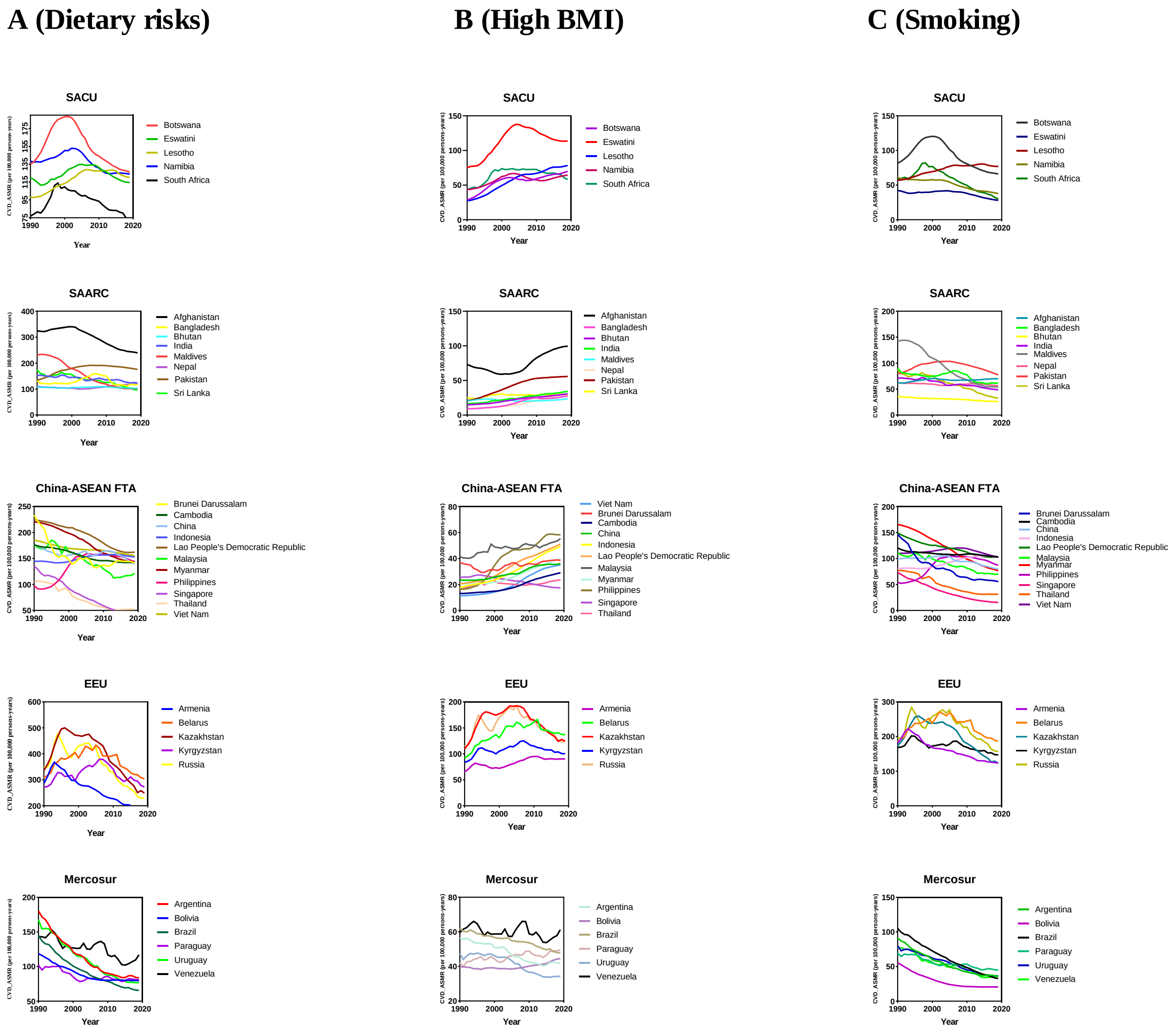

Figure S4: The temporal trend of CVD_ASMR (age-standardized mortality rate) for females attributable to dietary risks (A), high BMI (B) and smoking (C) across BRICS-Plus from 1990 to 2019.

A (dietary risks) B (high BMI) C (Smoking)

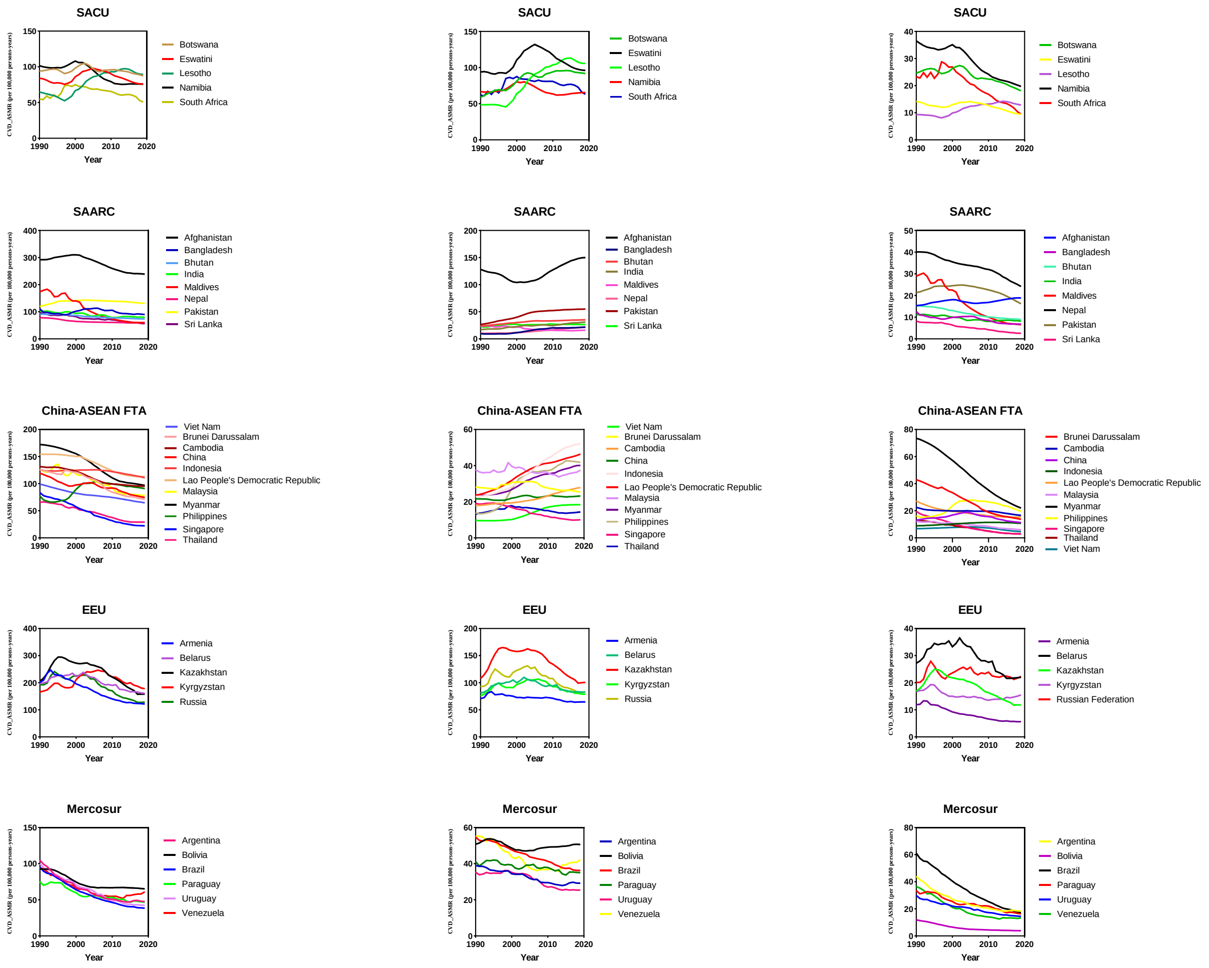

Figure S5: The temporal trend of IHD_ASMR (age-standardized mortality rate) for males attributable to dietary risks (A), high BMI (B) and smoking (C) across BRICS-Plus from 1990 to 2019.

A (dietary risks) **B (High BMI)** **C (Smoking)**

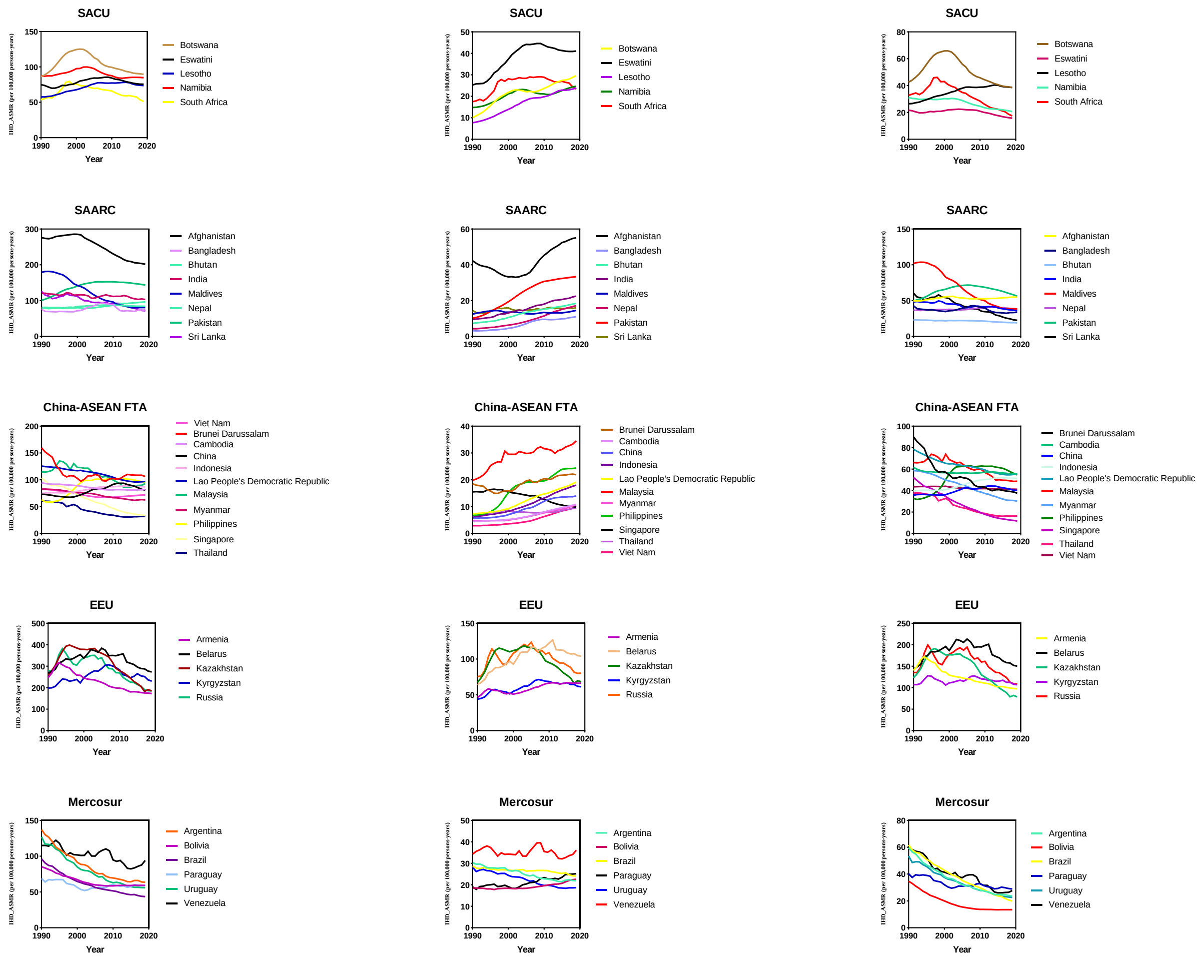

Figure S6: The temporal trend of IHD_ASMR (age-standardized mortality rate) for females attributable to dietary risks (A), high BMI (B) and smoking (C) across BRICS-Plus from 1990 to 2019.

A (dietary risks) **B (High BMI)** **C (Smoking)**

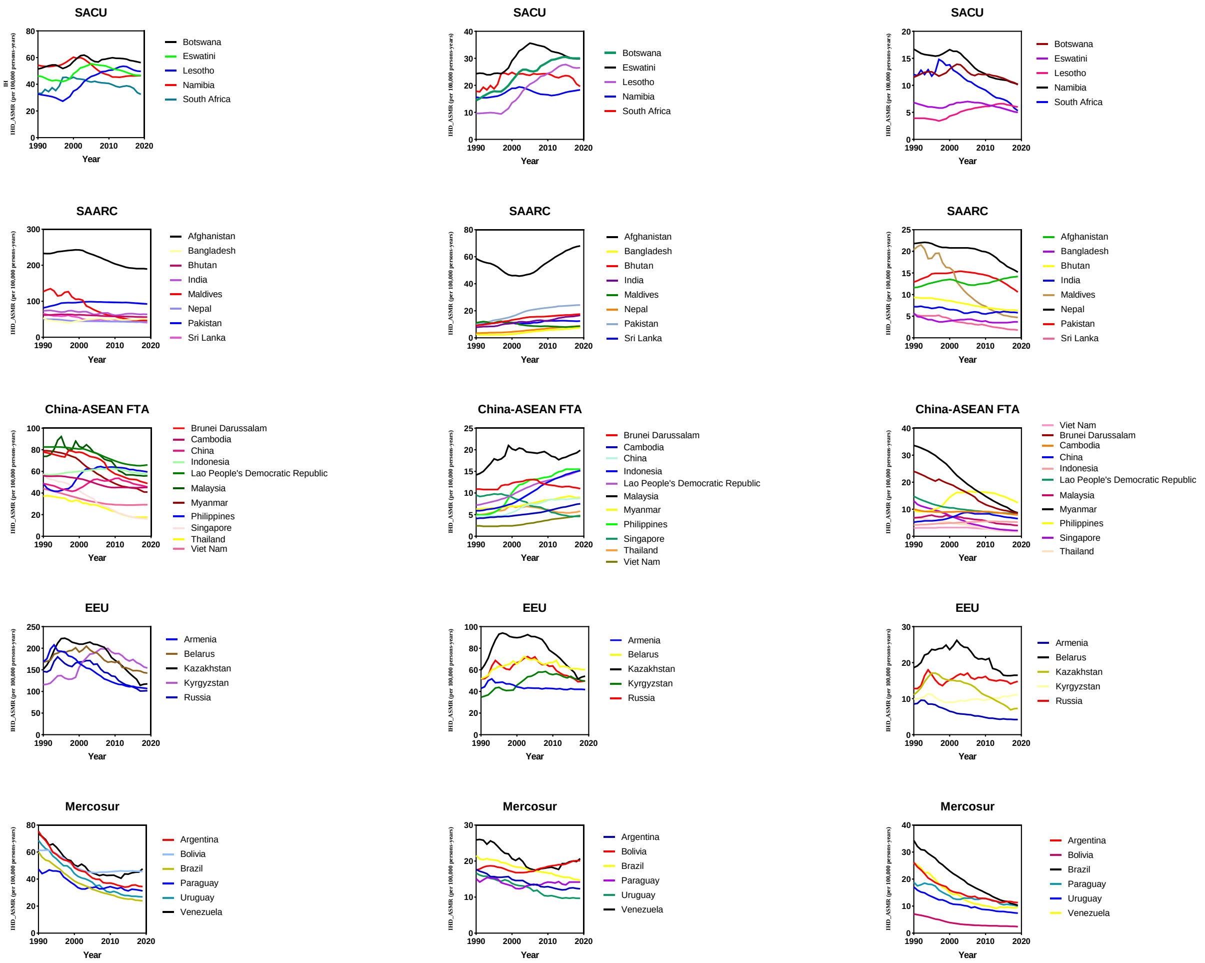

Figure S7: The temporal trend of IS_ASMR (age-standardized mortality rate) for males attributable to dietary risks (A), high BMI (B) and smoking (C) across BRICS-Plus from 1990 to 2019.

A (dietary risks)   B (High BMI)   C (Smoking)

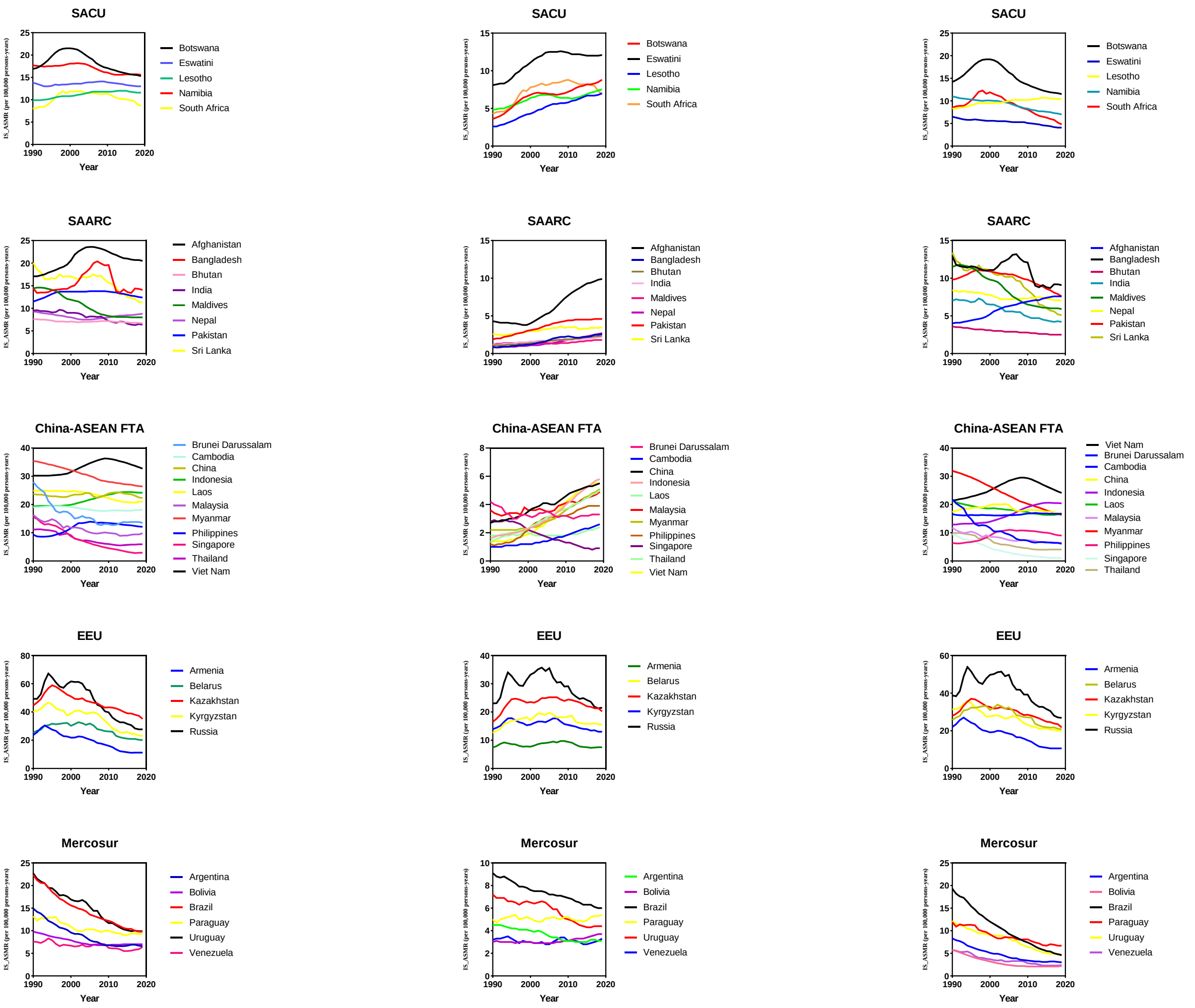

Figure S8: The temporal trend of IS_ASMR (age-standardized mortality rate) for females attributable to dietary risks (A), high BMI (B) and smoking (C) across BRICS-Plus from 1990 to 2019.

A (dietary risks) **B (High BMI)** **C (Smoking)**

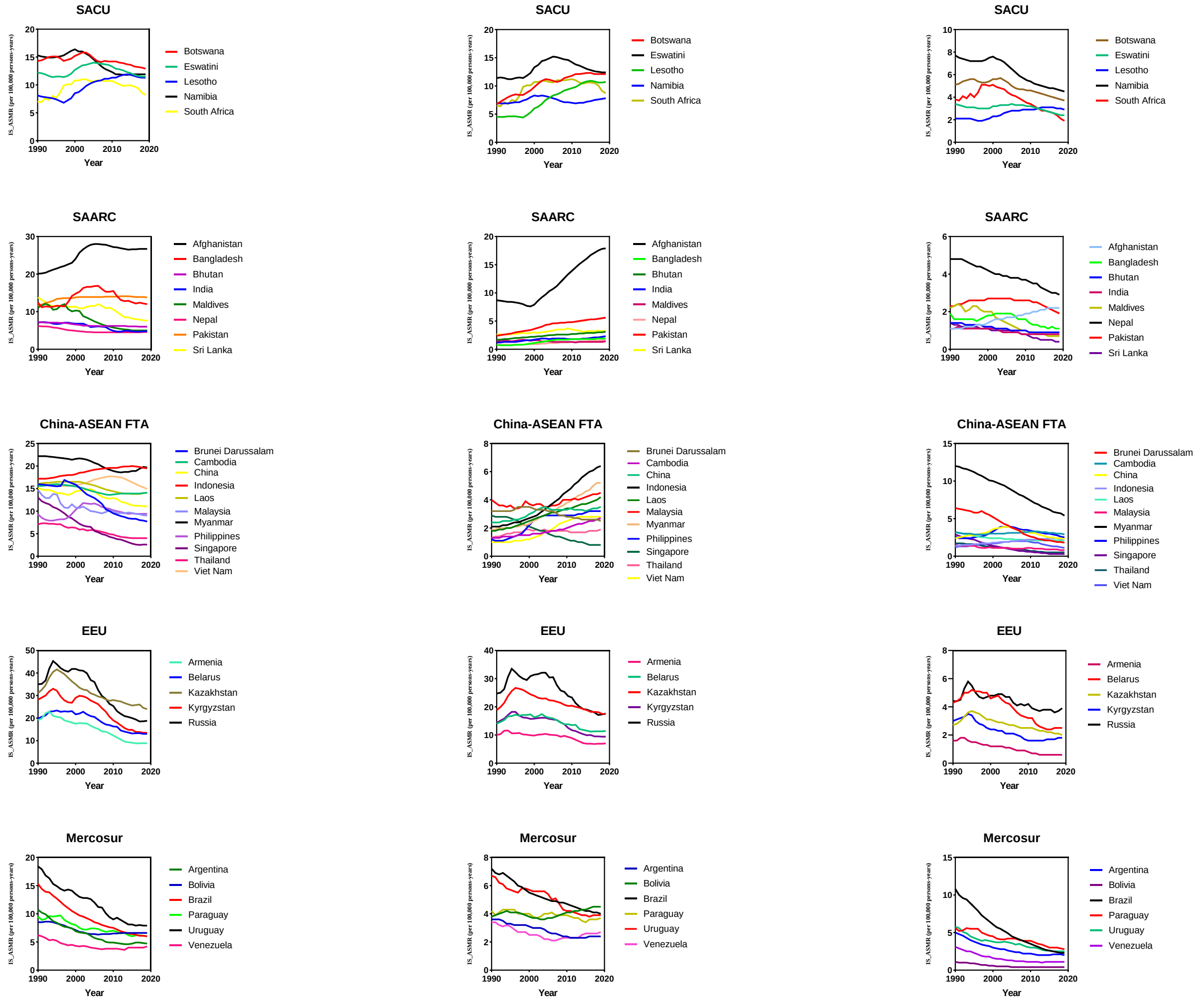

Figure S9: The temporal trend of CVD, IHD, and IS burden for different age groups attributable to dietary risks, high BMI, and smoking across BRICS-Plus from 1990 to 2019.

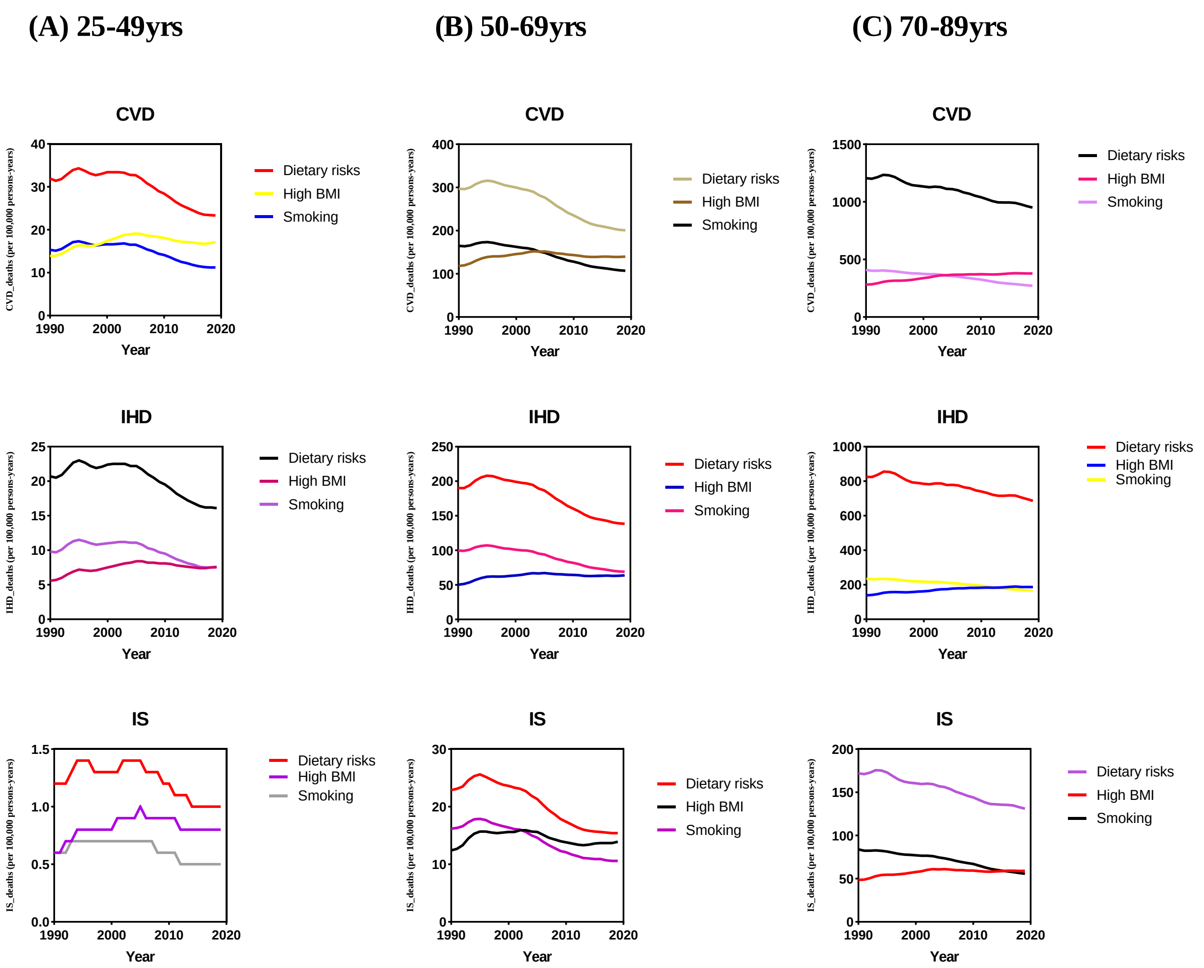

Figure S10: The temporal trend of age-specific (25-49 yrs) CVD mortality (per 100,000) attributable to dietary risks (A), high BMI (B) and smoking (C) across BRICS-Plus from 1990 to 2019.

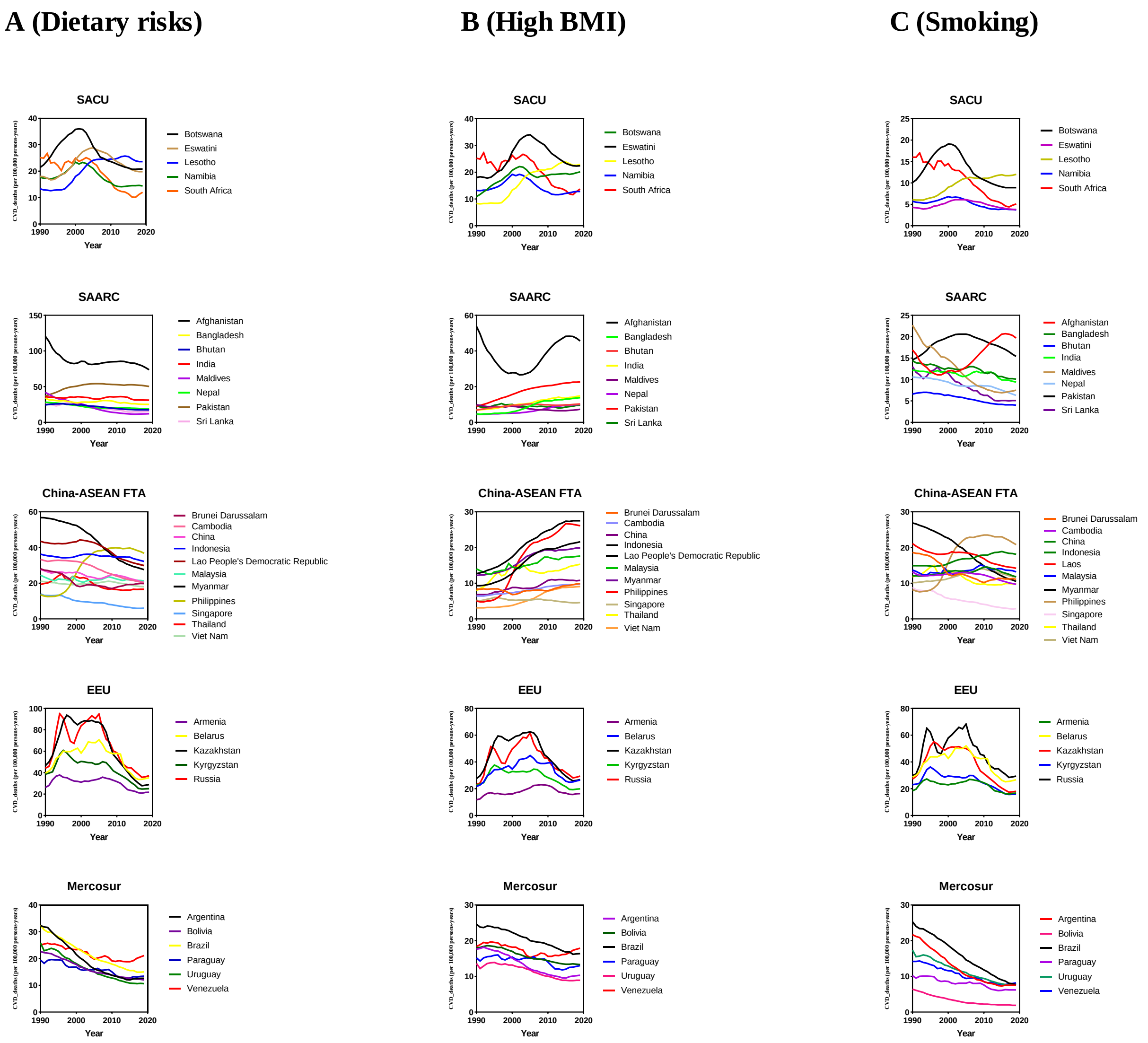

Figure S11: The temporal trend of age-specific (25-49 yrs) IHD mortality (per 100,000) attributable to dietary risks (A), high BMI (B) and smoking (C) across BRICS-Plus from 1990 to 2019.

A (Dietary risks) B (High BMI) C (Smoking)

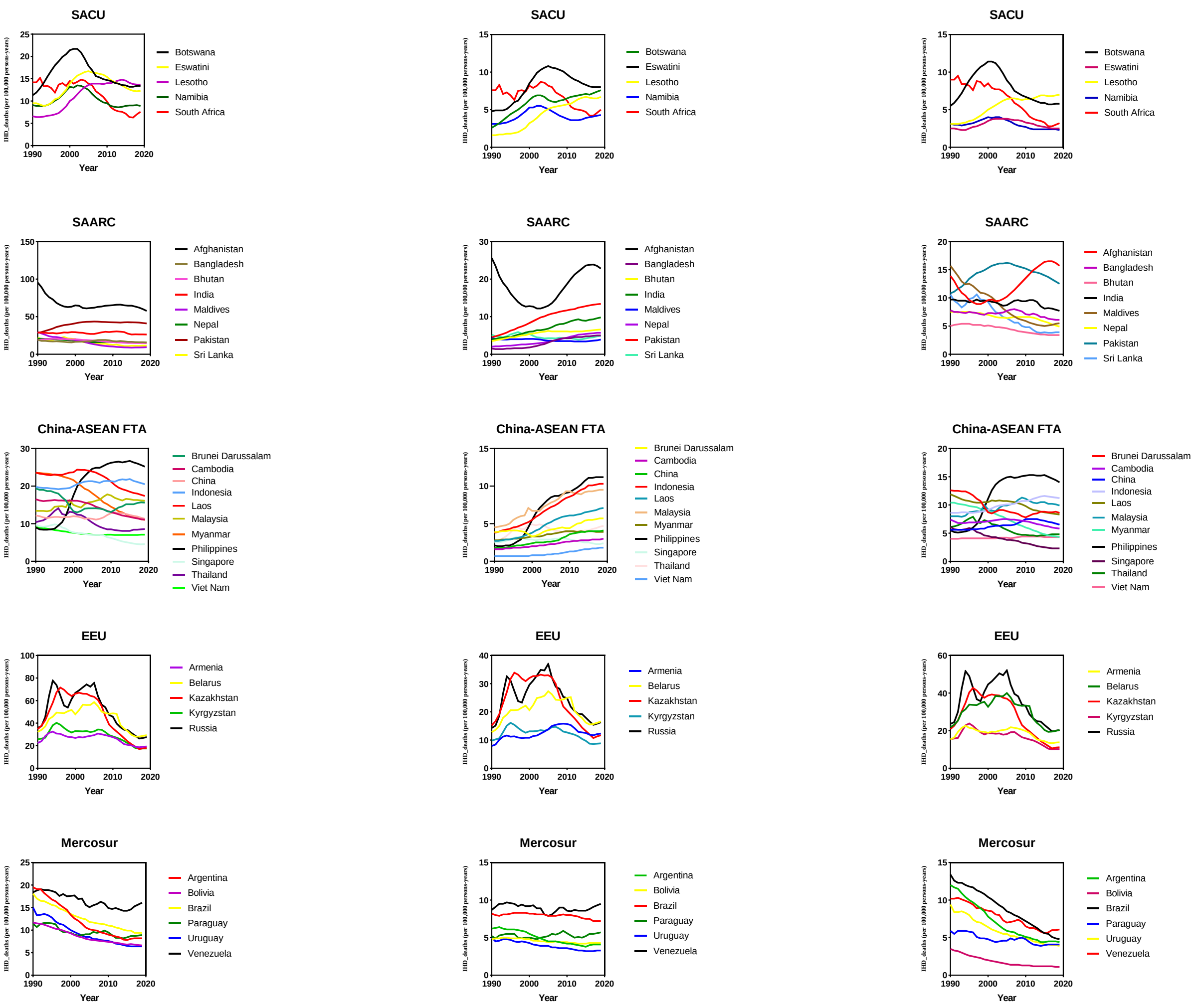

Figure S12: The temporal trend of age-specific (25-49 yrs) IS mortality (per 100,000) attributable to dietary risks (A), high BMI (B) and smoking (C) across BRICS-Plus from 1990 to 2019.

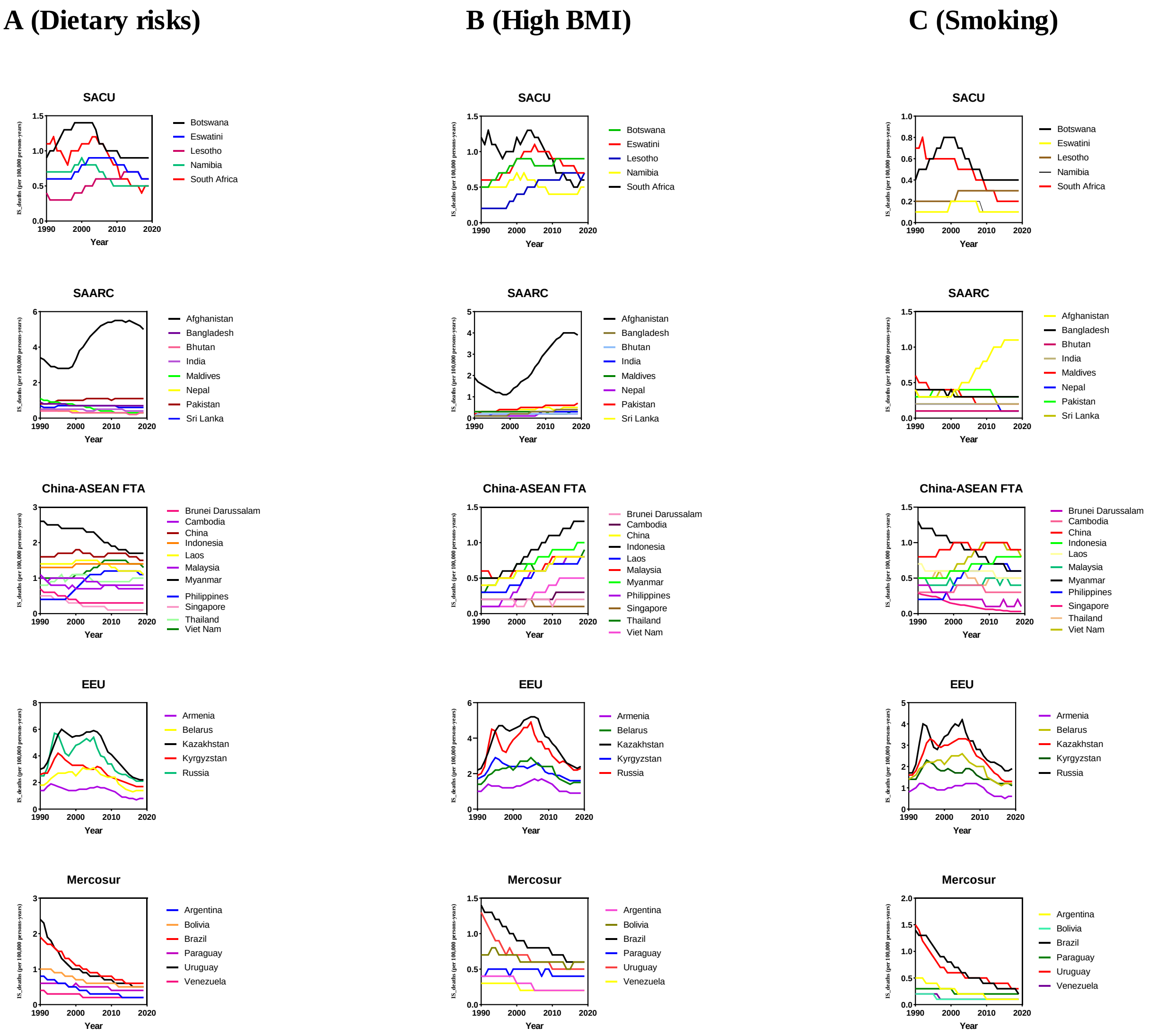

Figure S13: The temporal trend of age-specific (50-69 yrs) CVD mortality (per 100,000) attributable to dietary risks (A), high BMI (B) and smoking (C) across BRICS-Plus from 1990 to 2019.

A (Dietary risks) B (High BMI) C (Smoking)

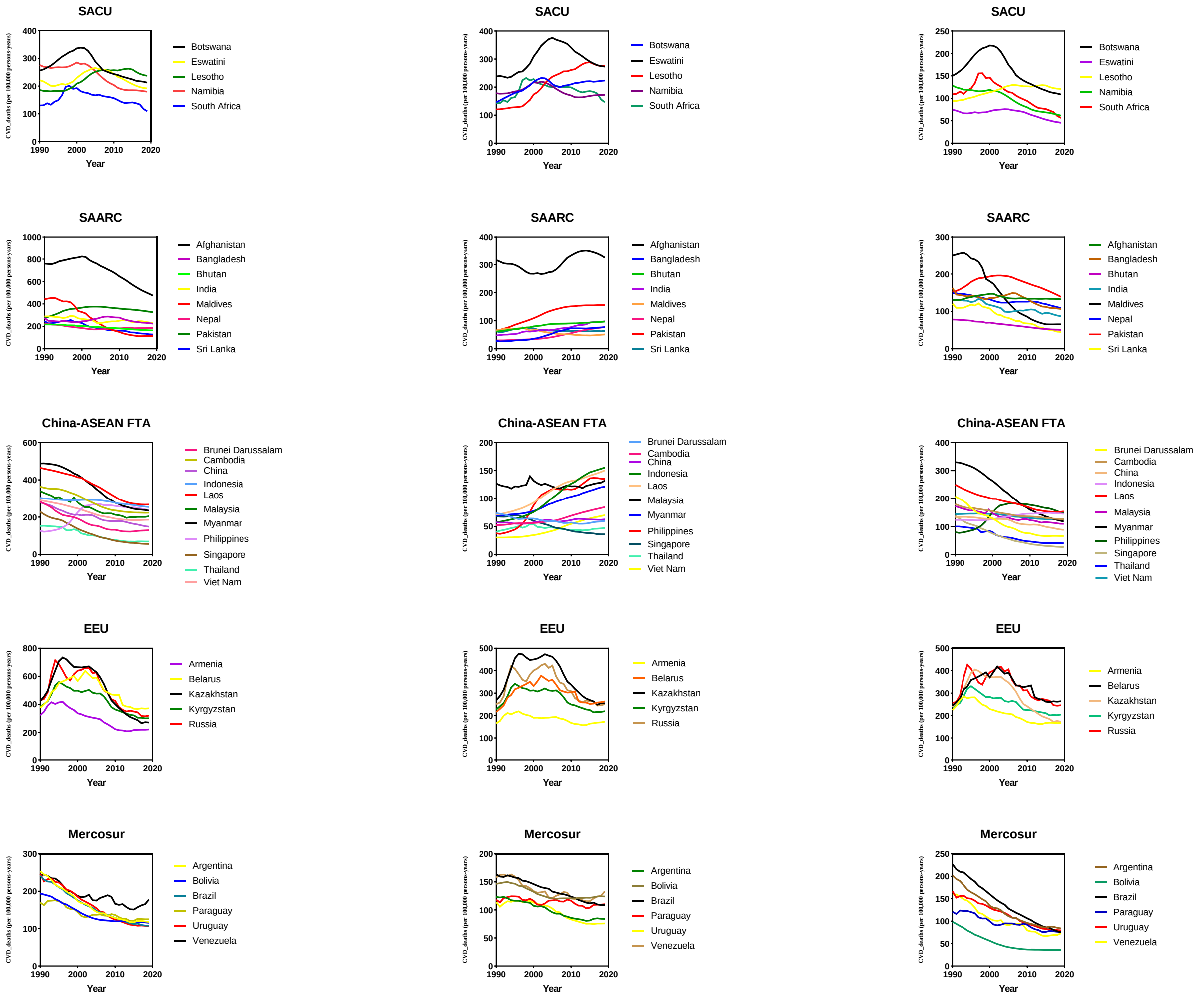

Figure S14: The temporal trend of age-specific (50-69 yrs) IHD mortality (per 100,000) attributable to dietary risks (A), high BMI (B) and smoking (C) across BRICS-Plus from 1990 to 2019.

A (Dietary risks)   B (High BMI)   C (Smoking)

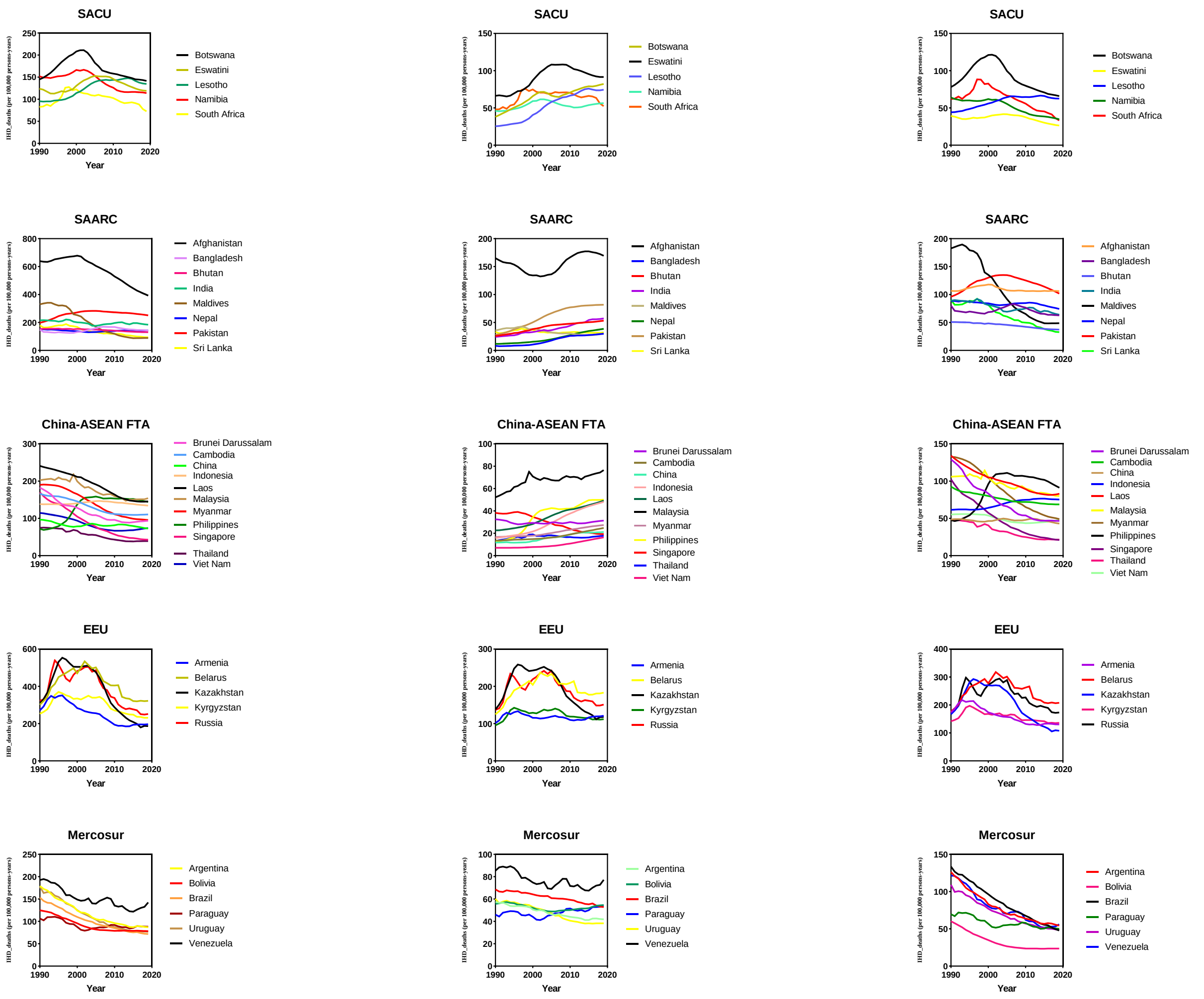

Figure S15: The temporal trend of age-specific (50-69 yrs) IS mortality (per 100,000) attributable to dietary risks (A), high BMI (B) and smoking (C) across BRICS-Plus from 1990 to 2019.

A (Dietary risks) B (High BMI) C (Smoking)

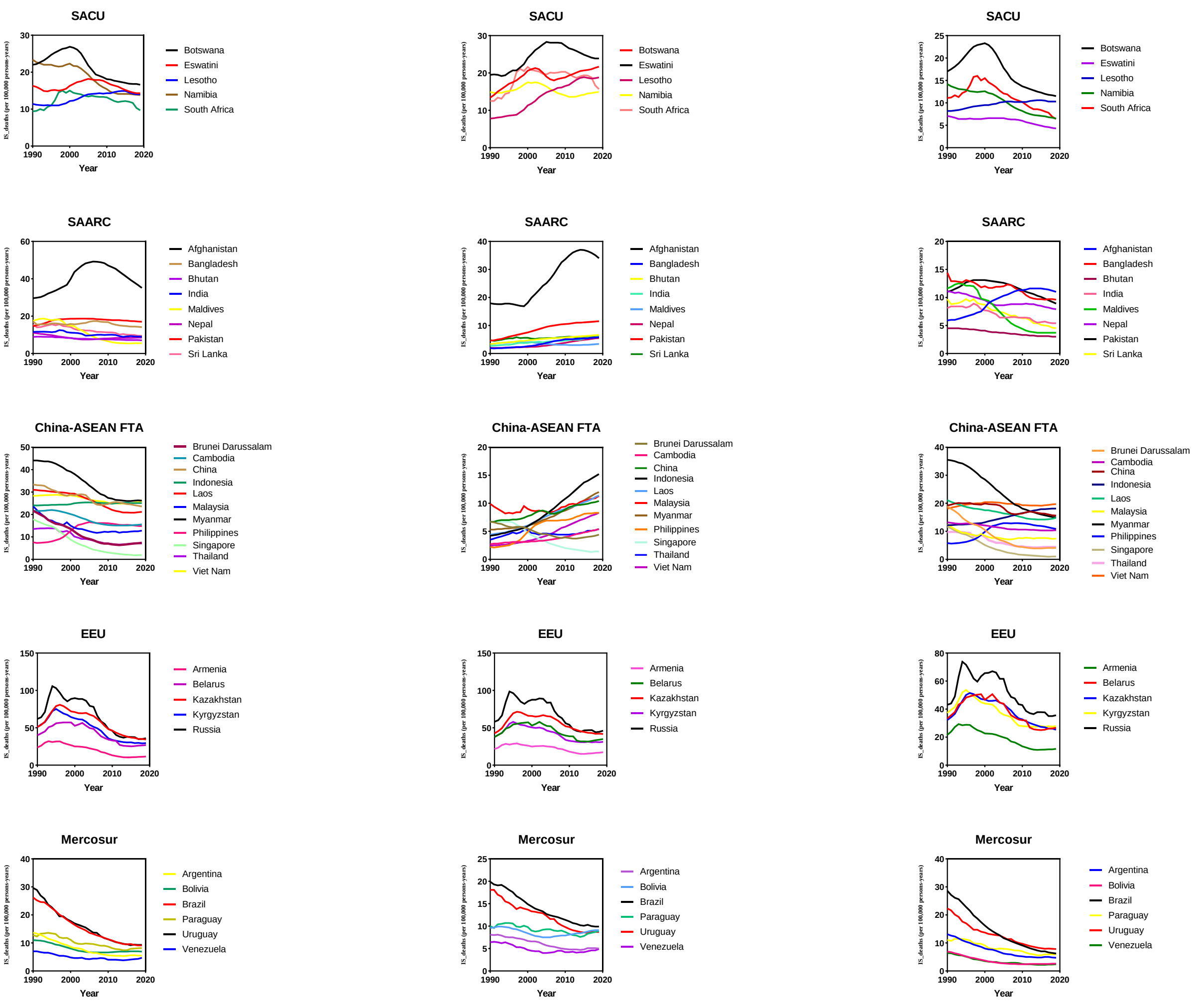

Figure S16: The temporal trend of age-specific (70-89 yrs) CVD mortality (per 100,000) attributable to dietary risks (A), high BMI (B) and smoking (C) across BRICS-Plus from 1990 to 2019.

A (Dietary risks)　　B (High BMI)　　C (Smoking)

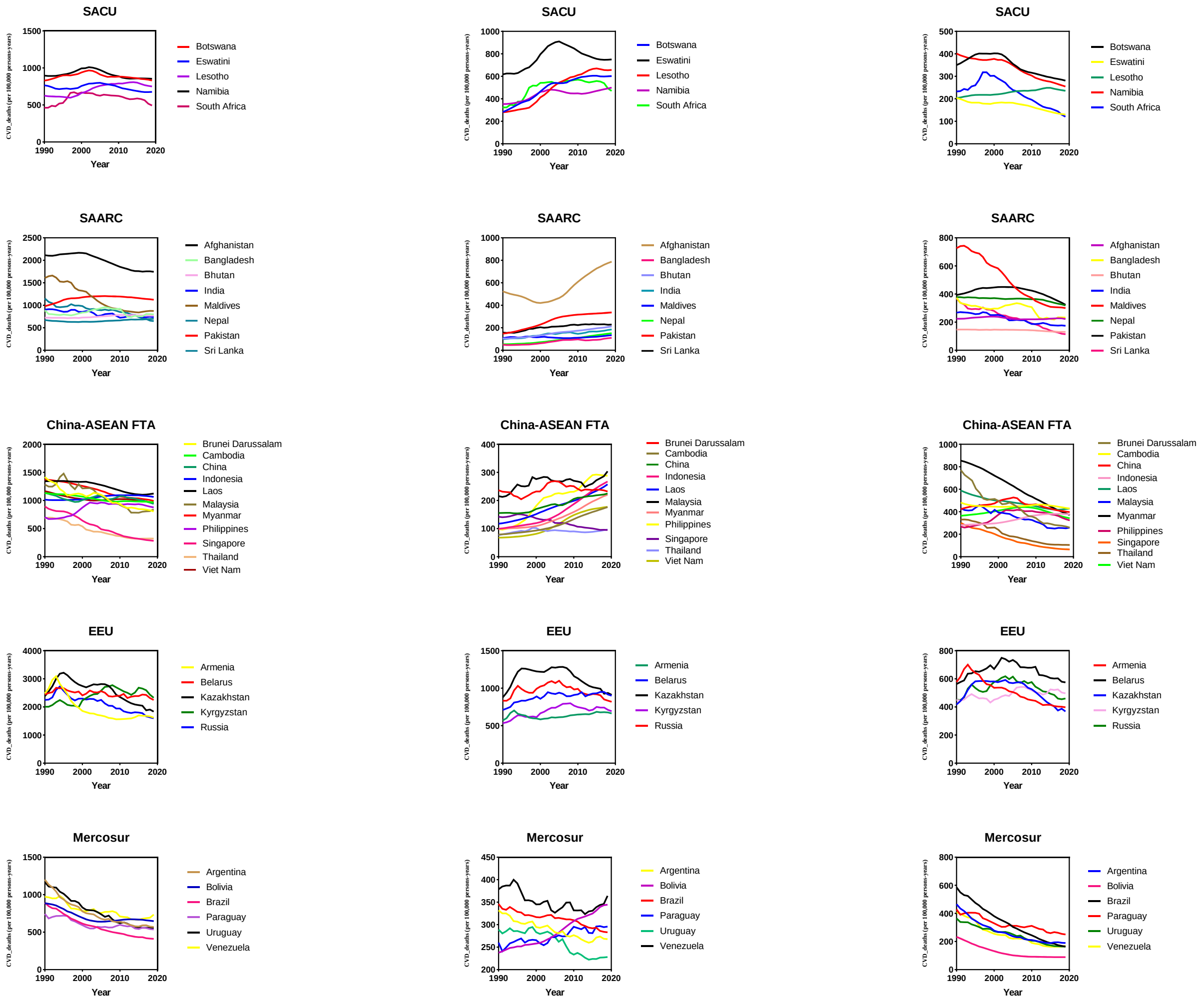

Figure S17: The temporal trend of age-specific (70-89 yrs) IHD mortality (per 100,000) attributable to dietary risks (A), high BMI (B) and smoking (C) across BRICS-Plus from 1990 to 2019.

A (Dietary risks) **B (High BMI)** **C (Smoking)**

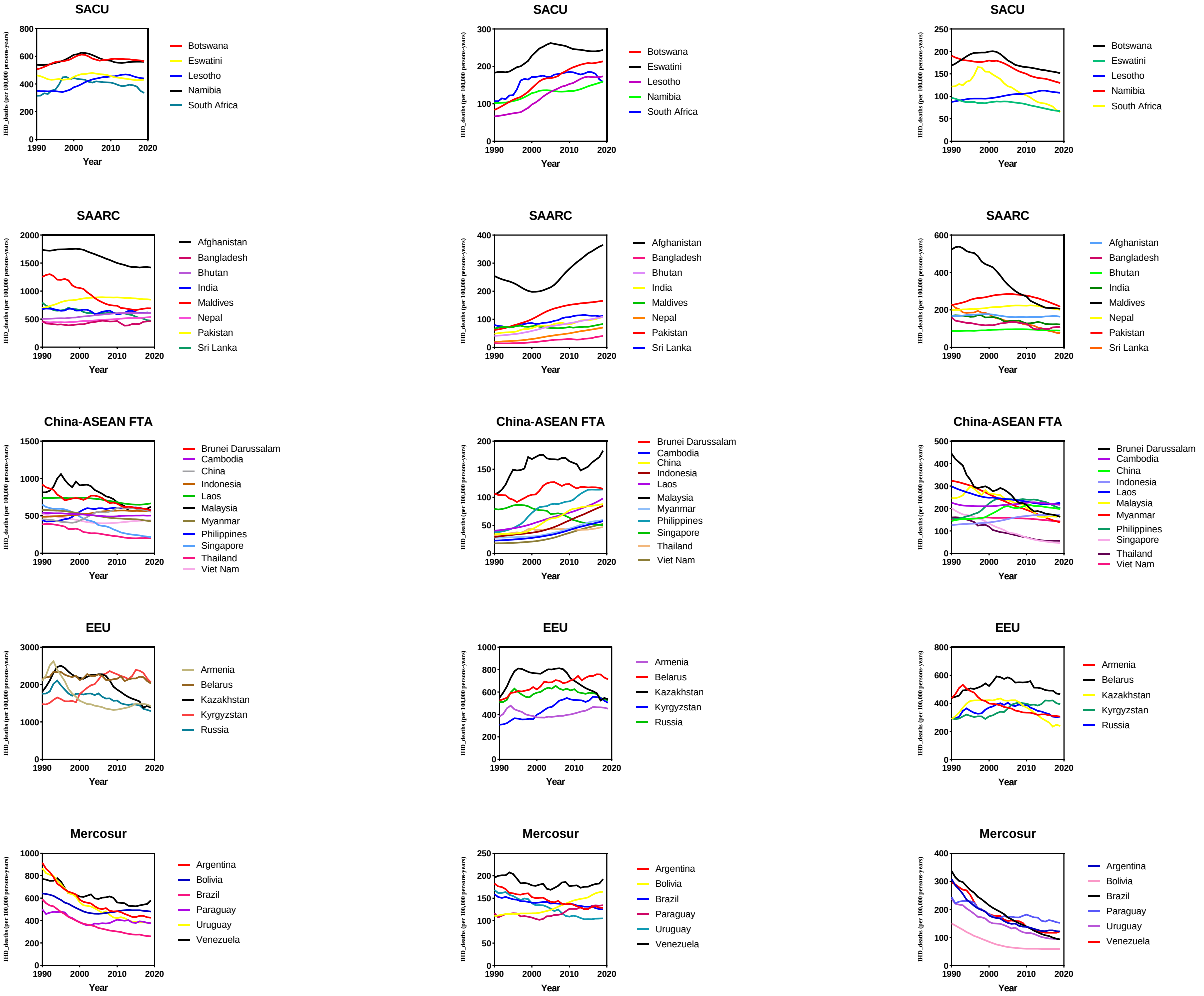

Figure S18: The temporal trend of age-specific (70-89 yrs) IS mortality (per 100,000) attributable to dietary risks (A), high BMI (B) and smoking (C) across BRICS-Plus from 1990 to 2019.

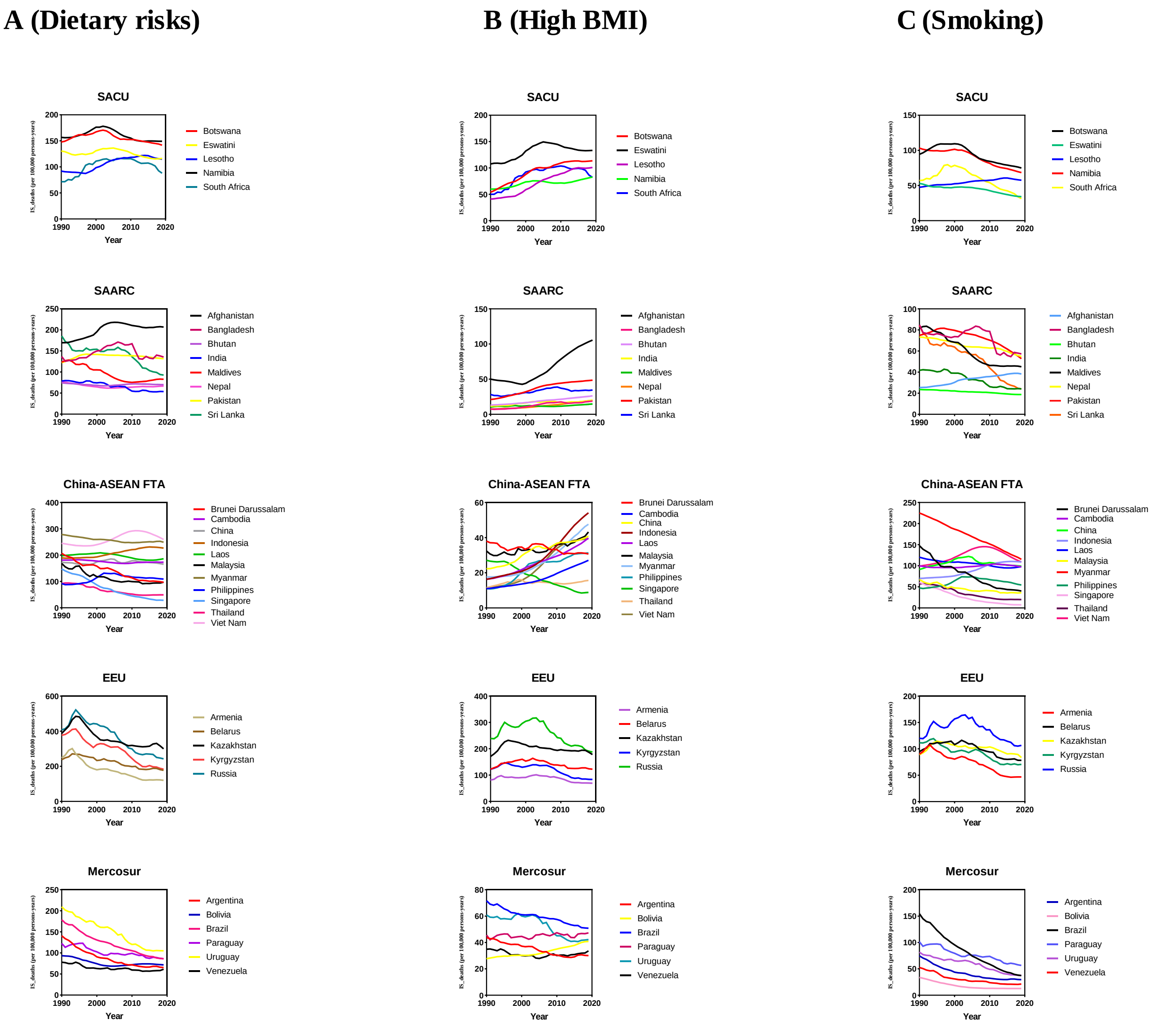

Figure S19: APC Analysis on CVD Mortality rate by BRICS-Plus countries.

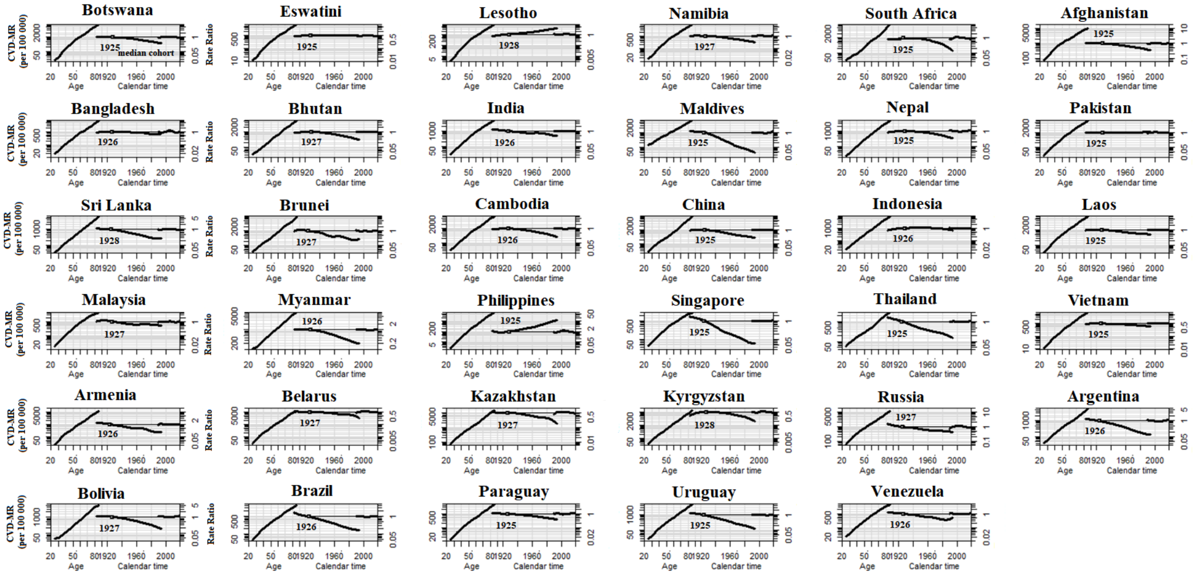

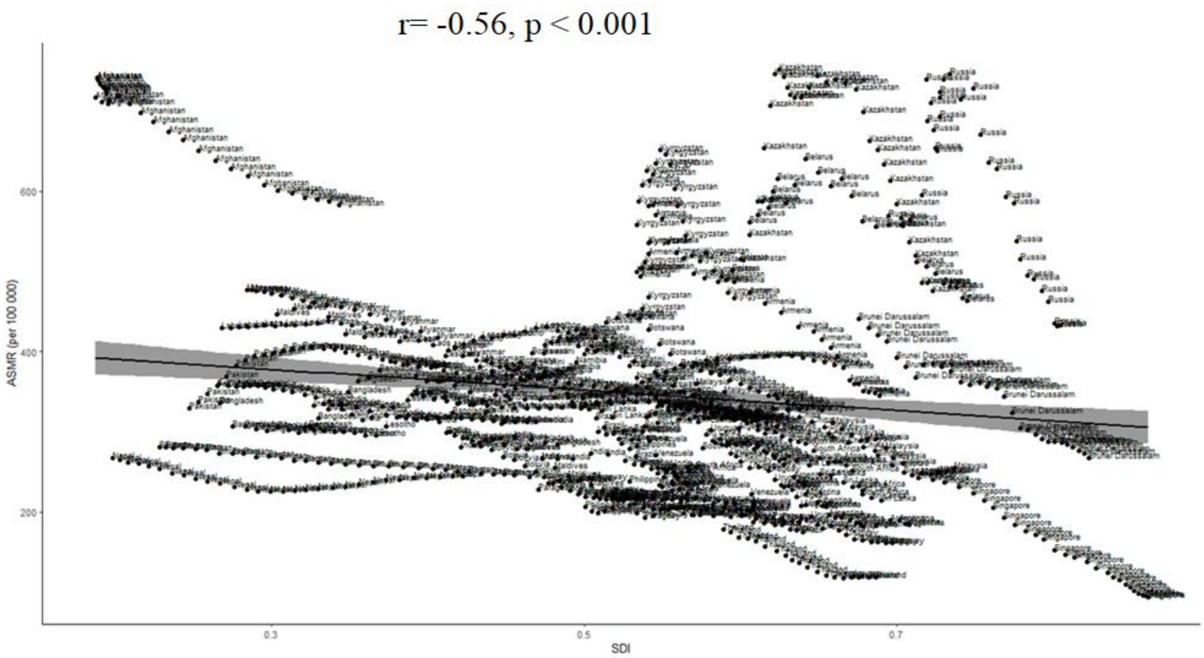

Figure S20: Correlation between countries' SDI and CVD age-standardized mortality rate across BRICS-Plus countries from 1990 to 2019.

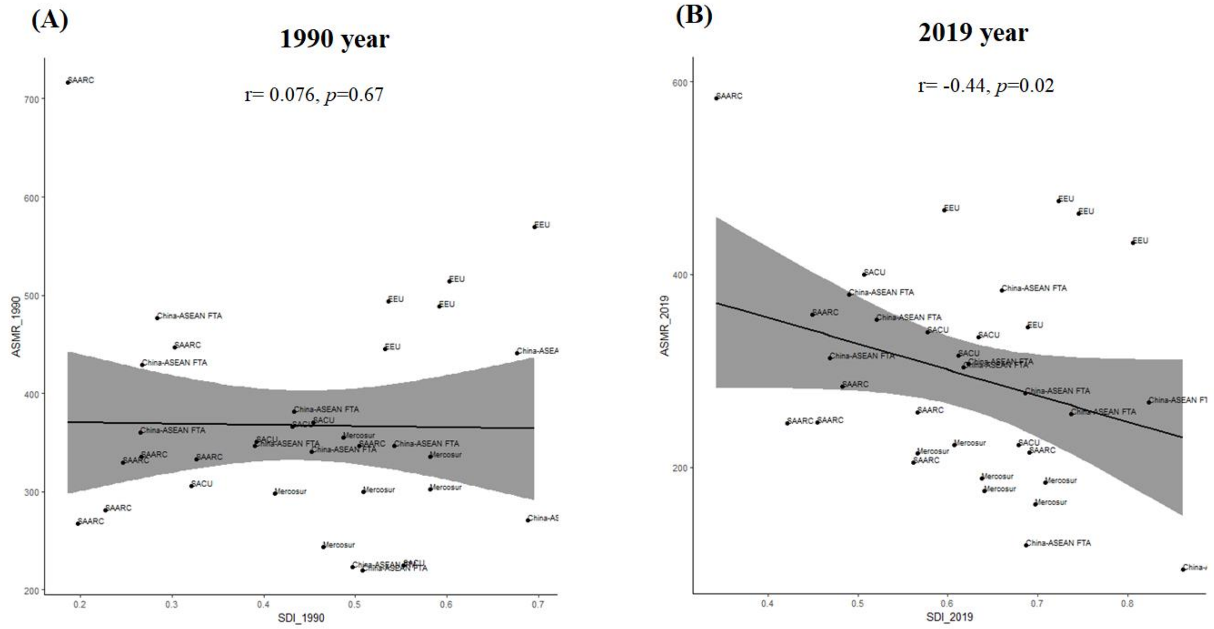

Figure S21. Correlation between countries' SDI and CVD age-standardized mortality rate across BRICS-Plus countries in year 1990 and 2019 separately.

Table S1: The average annual percent change in the burden of cause-specific IHD mortality for both sexes across BRICS-Plus from 1990 to 2019.

IHD	ASMR/100,000			Deaths, n×1000		
	1990 (95%UI)	2019 (95%UI)	AAPC (95%CI)	1990 (95%UI)	2019 (95%UI)	AAPC (95%CI)
**BRICS-Plus**	169 (190, 149)	143 (169, 119)	-0.6 (-0.7,-0.4)	2426 (2656, 2184)	5331 (6114, 4561)	2.8 (2.5,3.1)
**SACU**						
Botswana	122 (153, 98)	130 (170, 98)	0.3 (0.2, 0.4)	0.5 (0.7, 0.4)	1.4 (1.8, 1.1)	3.3 (3.2, 3.4)
Eswatini	113 (132, 93)	121 (158, 91)	0.2 (-0.1, 0.5)	0.3 (0.3, 0.2)	0.5 (0.7, 0.4)	2.5 (2.2, 2.8)
Lesotho	84 (103, 65)	123 (157, 91)	1.3 (1.2, 1.4)	0.6 (0.8, 0.5)	1.2 (1.6, 0.9)	2.1 (1.9, 2.2)
Namibia	122 (142, 103)	115 (138, 93)	-0.2 (-0.3, -0.1)	0.7 (0.8, 0.6)	1.3 (1.6, 1.1)	2.3 (2.1, 2.4)
South Africa	80 (89, 70)	81 (87, 73)	0.1 (-0.5, 0.6)	15 (16, 13)	30 (32, 27)	2.4 (1.9, 3.0)
**SAARC**						
Afghanistan	410 (493, 337)	320 (385, 253)	-0.8 (-0.9, -0.8)	26 (31, 21)	34 (42, 26)	1.0 (0.9, 1.1)
Bangladesh	114 (129, 97)	111 (135, 86)	0.1 (-0.4, 0.6)	47 (54, 40)	131 (160, 102)	3.8 (3.3, 4.3)
Bhutan	121 (156, 90)	134 (162, 105)	0.3 (0.3, 0.4)	0.2 (0.3, 0.1)	0.6 (0.8, 0.5)	3.4 (3.3, 3.5)
India	170 (190, 150)	150 (172, 129)	-0.4 (-1.3, 0.4)	621 (691, 550)	1519 (1745, 1311)	3.1 (2.5, 3.7)
Maldives	265 (300, 238)	120 (141, 101)	-2.8 (-3.1, -2.4)	0.2 (0.2, 0.1)	0.3 (0.3, 0.2)	1.7 (1.4, 2.0)
Nepal	109 (138, 88)	123 (148, 98)	0.4 (0.3, 0.6)	8.9 (11.3, 7.1)	23 (28, 18)	3.4 (3.3, 3.5)

Pakistan	146 (169, 121)	189 (225, 158)	0.9 (0.9, 1.0)	75 (87, 63)	183 (220, 152)	3.1 (3.0, 3.2)
Sri Lanka	174 (189, 159)	109 (138, 81)	-1.4 (-2.1, -0.6)	15 (16, 14	23 (30, 17)	1.6 (1.0, 2.3)
**China-ASEAN FTA**						
Brunei Darussalam	204 (224, 185)	132 (147, 117)	-1.5 (-1.8, -1.1)	0.2 (0.2, 0.1)	0.3 (0.3, 0.2)	1.9 (1.7, 2.2)
Cambodia	122 (151, 102)	118 (139, 96)	0.1 (-0.2, 0.1)	4.6 (5.7, 3.8)	11 (13, 9)	3.2 (3.1, 3.3)
China	98 (110, 88)	116 (131, 100)	0.5 (0.3, 0.7)	607 (678, 536)	1874 (2131, 1612)	3.9 (3.7, 4.2)
Indonesia	118 (132, 103)	140 (154, 119)	0.6 (0.6, 0.7)	97 (109, 86)	245 (275, 207)	3.2 (3.2, 3.3)
Laos	171 (217, 137)	162 (189, 134)	-0.2 (-0.3, -0.1)	2.9 (3.7, 2.3)	5.6 (6.8, 4.6)	2.3 (2.2, 2.4)
Malaysia	165 (176, 152)	145 (175, 117)	-0.4 (-0.9, 0.2)	13 (14, 12)	34 (41, 27)	3.4 (2.8, 4.0)
Myanmar	145 (178, 117)	104 (119, 91)	-1.2 (-1.2, -1.1)	28 (36, 22)	40 (46, 35)	1.1 (1.1, 1.2)
Philippines	103 (115, 91)	148 (171, 123)	1.3 (1.0, 1.6)	20 (24, 17)	97 (114, 79)	5.7 (5.4, 6.0)
Singapore	150 (156, 141)	52 (56, 45)	-3.6 (-3.9, -3.3)	2.8 (2.9, 2.7)	3.8 (4.1, 3.3)	1.1 (0.8, 1.3)
Thailand	92 (103, 81)	52 (66, 39)	-1.9 (-2.4, -1.4)	25 (28, 22)	51 (65, 39)	2.4 (2.0, 2.8)
Viet Nam	103 (122, 85)	95 (112, 79)	-0.3 (-0.3, -0.2)	36 (43, 30)	74 (88, 61)	2.5 (2.4, 2.5)
**EEU**						
Armenia	328 (344, 307)	237 (272, 201)	-1.1 (-1.4, -0.9)	7.1 (7.3, 6.6)	9.2 (10, 7.8)	0.9 (0.7, 1.2)
Belarus	332 (346, 309)	334 (408, 274)	0.1 (-0.4, 0.4)	40 (41, 37)	54 (66, 44)	1.1 (0.7, 1.5)

Kazakhstan	303 (316, 284)	251 (281, 219)	-0.6 (-1.0, -0.3)	32 (34, 30)	33 (37, 28)	0.1 (-0.4, 0.5)
Kyrgyzstan	235 (249, 218)	320 (354, 283)	1.2 (0.5, 2.0)	6.6 (7.1, 6.2)	11 (12, 10)	2.0 (1.3, 2.8)
Russia	315 (324, 298)	240 (270, 208)	-0.9 (-1.8, -0.1)	491 (503, 471)	562 (632, 488)	0.5 (-0.4, 1.4)
**Mercosur**						
Argentina	170 (177, 158)	82 (87, 74)	-2.5 (-2.8, -2.1)	49 (51, 46)	45 (48, 41)	-0.3 (-0.8, 0.3)
Bolivia	140 (182, 104)	105 (136, 76)	-0.9 (-1.1, -0.8)	3.6 (4.8, 2.6)	7.7 (10, 5.4)	2.6 (2.4, 2.7)
Brazil	157 (163, 146)	74 (79, 67)	-2.5 (-2.7, -2.3)	117 (121, 111)	171 (180, 156)	1.3 (1.0, 1.7)
Paraguay	109 (121, 94)	89 (112, 70)	-0.6 (-1.0, -0.2)	2.2 (2.4, 1.9)	4.7 (5.9, 3.7)	2.8 (2.4, 3.2)
Uruguay	155 (161, 144)	67 (71, 60)	-2.9 (-3.5, -2.3)	5.9 (6.1, 5.5)	4 (4.3, 3.6)	-1.2 (-1.9, -0.5)
Venezuela	174 (182, 161)	130 (164, 102)	-1.2 (-1.7, -0.6)	15 (15, 14)	36 (46, 28)	2.9 (2.3, 3.5)

Note: AAPC, average annual percent change; ASMR, age-standardized mortality rate; IHD, ischemic heart diseases; BRICS, Brazil, Russia, India, China, and South Africa; SACU, South African Customs Union; SAARC, South Asian Association for Regional Cooperation; China-ASEAN FTA, China-ASEAN Free Trade Area; and EEU, Eurasian Economic Union.

Table S2: The average annual percent change in the burden of cause-specific IS mortality for both sexes across BRICS-Plus from 1990 to 2019.

IS	ASMR/100,000			Deaths, n×1000		
	1990 (95%UI)	2019 (95%UI)	AAPC (95%CI)	1990 (95%UI)	2019 (95%UI)	AAPC (95%CI)
**BRICS-Plus**	66 (78, 55)	55 (67, 44)	-0.6 (-0.7,-0.5)	1021 (1186, 8933)	2120 (2458, 1784)	2.5 (2.1, 3.0)
**SACU**						
Botswana	78 (96, 62)	74 (97, 55)	-0.2 (-0.3, 0.1)	0.3 (0.3, 0.2)	0.6 (0.8, 0.4)	2.6 (2.6, 2.7)
Eswatini	69 (82, 55)	70 (90, 53)	0.1 (-0.1, 0.3)	0.2 (0.2, 0.1)	0.3 (0.3, 0.2)	2.2 (2.0, 2.3)
Lesotho	53 (67, 42)	74 (95, 56)	1.1 (1.0, 1.2)	0.3 (0.4, 0.3)	0.6 (0.7, 0.4)	1.5 (1.3, 1.6)
Namibia	74 (90, 58)	69 (83, 57)	-0.3 (-0.4, -0.1)	0.4 (0.4, 0.3)	0.7 (0.8, 0.6)	2.2 (2.1, 2.3)
South Africa	41 (47, 35)	49 (53, 44)	0.8 (-0.1, 1.6)	6.9 (7.8, 5.8)	16 (17, 15)	3.1 (2.3, 3.9)
**SAARC**						
Afghanistan	79 (105, 55)	98 (129, 69)	0.7 (0.5, 1.0)	4.1 (5.7, 2.9)	8.6 (11, 6.2)	2.6 (2.5, 2.7)
Bangladesh	60 (78, 44)	60 (76, 45)	0.1 (-0.6, 0.8)	20 (27, 15)	61 (79, 45)	4.0 (3.2, 4.8)
Bhutan	36 (54, 26)	33 (42, 26)	-0.3 (-0.4, -0.2)	0.05 (0.08, 0.03)	0.2 (0.2, 0.1)	3.5 (3.5, 3.6)
India	41 (52, 33)	30 (35, 25)	-1.1 (-1.9, -0.2)	109 (140, 85)	271 (320, 227)	3.1 (2.3, 3.9)
Maldives	50 (65, 40)	29 (35, 24)	-1.9 (-2.3, -1.5)	0.02 (0.03, 0.02)	0.06 (0.08, 0.05)	3.5 (3.1, 3.8)
Nepal	38 (61, 24)	35 (48, 26)	-0.2 (-0.4, -0.1)	2.3 (3.9, 1.4)	5.9 (8.2, 4.2)	3.2 (3.2, 3.3)
Pakistan	48 (65, 33)	52 (68, 43)	0.2 (0.2, 0.3)	22 (30, 15)	39 (51, 32)	1.9 (1.9, 2.0)

Sri Lanka	72 (82, 63)	45 (58, 34)	-1.6 (-2.0, -1.1)	5.2 (5.9, 4.5)	9.1 (11, 6.7)	1.9 (1.5, 2.3)
**China-ASEAN FTA**						
Brunei Darussalam	81 (91, 71)	41 (47, 36)	-2.3 (-2.9, -1.7)	0.04 (0.04, 0.03)	0.05 (0.06, 0.05)	1.0 (0.4, 1.5)
Cambodia	59 (75, 47)	62 (74, 50)	0.2 (0.1, 0.2)	1.7 (2.2, 1.4)	5.1 (6.1, 4.1)	3.8 (3.7, 3.8)
China	64 (76, 56)	62 (70, 53)	-0.1 (-0.5, 0.4)	379 (456, 331)	1029 (1176, 881)	3.5 (3.2, 3.8)
Indonesia	67 (80, 56)	96 (111, 76)	1.3 (1.2, 1.3)	43 (51, 35)	136 (160, 106)	4.1 (4.0, 4.1)
Laos	69 (88, 52)	71 (88, 58)	0.1 (0.1, 0.2)	0.9 (1.2, 0.7)	2.1 (2.6, 1.7)	2.8 (2.7, 2.8)
Malaysia	53 (59, 47)	40 (51, 31)	-1.1 (-2.4, 0.3)	3.8 (4.2, 3.4)	8.2 (10, 6.4)	2.7 (1.7, 3.6)
Myanmar	95 (122, 74)	93 (107, 78)	-0.1 (-0.2, 0.1)	15 (19, 11)	32 (37, 26)	2.6 (2.5, 2.6)
Philippines	37 (42, 33)	42 (49, 36)	0.4 (0.1, 0.8)	6.3 (7.6, 5.6)	24 (28, 20)	4.7 (4.4, 5.0)
Singapore	57 (60, 52)	12 (14, 10)	-5.0 (-5.5, -4.4)	0.9 (0.9, 0.8)	0.8 (1.0, 0.7)	-0.1 (-0.6, 0.4)
Thailand	32 (38, 26)	20 (27, 15)	-1.5 (-2.0, -1.0)	8.4 (10, 6.9)	20 (26, 15)	3.0 (2.6, 3.5)
Viet Nam	77 (97, 61)	85 (100, 68)	0.4 (0.3, 0.4)	25 (32, 20)	62 (73, 50)	3.2 (3.1, 3.3)
**EEU**						
Armenia	81 (89, 72)	45 (52, 37)	-2.0 (-2.9, -1.1)	1.6 (1.8, 1.4)	1.7 (2.1, 1.4)	-0.1 (-1.1, 0.9)
Belarus	91 (97, 84)	75 (94, 58)	-0.6 (-1.1, -0.2)	11 (12, 10)	12 (15, 9.7)	0.4 (-0.1, 0.8)
Kazakhstan	111 (124, 100)	105 (119, 91)	-0.2 (-0.5, 0.1)	11 (13, 10)	13 (15, 11)	0.5 (0.1, 1.0)
Kyrgyzstan	108 (116, 99)	69 (77, 60)	-1.7 (-2.8, -0.6)	3.1 (3.2, 2.7)	2.5 (2.8, 2.2)	-0.7 (-1.4, 0.1)

Russia	172 (177, 162)	107 (120, 92)	-1.6 (-2.1, -1.1)	262 (269, 251)	254 (285, 220)	-0.1 (-0.6, 0.4)
**Mercosur**						
Argentina	45 (50, 39)	22 (25, 19)	-2.4 (-2.7, -2.0)	12 (14, 11)	12 (14, 11)	-0.1 (-0.4, 0.4)
Bolivia	41 (53, 31)	32 (42, 23)	-0.8 (-1.0, -0.6)	0.9 (1.2, 0.7)	2.1 (2.9, 1.5)	2.8 (2.7, 2.9)
Brazil	80 (84, 72)	33 (36, 29)	-2.9 (-3.1, -2.7)	52 (55, 48)	74 (80, 65)	1.2 (1.0, 1.4)
Paraguay	49 (56, 42)	35 (46, 27)	-1.1 (-1.7, -0.4)	0.9 (1.0, 0.8)	1.7 (2.3, 1.3)	2.3 (1.5, 3.0)
Uruguay	70 (75, 64)	35 (39, 30)	-2.3 (-2.7, -1.9)	2.6 (2.8, 2.4)	2.3 (2.6, 2.1)	-0.3 (-0.8, 0.2)
Venezuela	33 (37, 28)	25 (32, 19)	-0.9 (-1.5, -0.2)	2.5 (2.8, 2.2)	6.6 (8.3, 5.1)	3.4 (2.7, 4.0)

Note: AAPC, average annual percent change; ASMR, age-standardized mortality rate; IS, ischemic stroke; BRICS, Brazil, Russia, India, China, and South Africa; SACU, South African Customs Union; SAARC, South Asian Association for Regional Cooperation; China-ASEAN FTA, China-ASEAN Free Trade Area; and EEU, Eurasian Economic Union.

Table S3: The average annual percent change in the burden of IHD mortality for both sexes attributed to modifiable risk factors across BRICS-Plus from 1990 to 2019.

**Population**	**IHD (ASMR/100,000)**		
	Dietary risks (AAPC (95%CI)	High BMI (AAPC (95%CI)	Smoking (AAPC (95%CI)
**BRICS-Plus**	-0.8 (-0.9, -0.7)	1.0 (0.8, 1.2)	-1.3 (-1.5,-1.1)
**SACU**			
Botswana	0.2 (0.1, 0.3)	3.0 (2.8, 3.3)	-0.4 (-0.6, -0.3)
Eswatini	-0.1 (-0.3, 0.3)	1.1 (0.8, 1.4)	-1.2 (-1.5, -1.0)
Lesotho	1.1 (1.0, 1.3)	3.7 (3.6, 3.9)	1.3 (1.1, 1.4)
Namibia	-0.3 (-0.5, -0.2)	1.2 (1.0, 1.4)	-1.5 (-1.7, -1.3)
South Africa	-0.1 (-0.8, 0.5)	0.6 (-0.1, 1.4)	-2.4 (-3.0, -1.8)
**SAARC**			
Afghanistan	-0.9 (-1.0, -0.8)	0.7 (0.5, 1.0)	0.3 (0.2, 0.4)
Bangladesh	-0.1 (-0.5, 0.5)	4.1 (3.6, 4.6)	-0.9 (-1.3, -0.5)
Bhutan	0.1 (-0.1, 0.1)	2.8 (2.6, 3.0)	-0.7 (-0.8, -0.5)
India	-0.5 (-1.3, 0.3)	2.8 (2.3, 3.4)	-1.1 (-1.4, -0.8)
Maldives	-3.1 (-3.4, -2.8)	-0.1 (-0.3, 0.3)	-3.8 (-4.1, -3.5)
Nepal	0.2 (0.1, 0.3)	4.2 (4.1, 4.4)	-0.4 (-0.5, -0.3)
Pakistan	0.9 (0.8, 1.0)	3.7 (3.6, 3.8)	0.1 (0.1, 0.3)
Sri Lanka	-1.7 (-2.4, -1.0)	0.6 (-0.1, 1.3)	-3.7 (-4.3, -3.2)

**China-ASEAN FTA**			
Brunei Darussalam	-1.6 (-1.9, -1.2)	0.3 (-0.1, 0.7)	-3.2 (-3.5, -2.9)
Cambodia	-0.6 (-0.7, -0.6)	2.6 (2.5, 2.8)	-0.6 (-0.6, -0.5)
China	0.1 (-0.2, 0.2)	2.7 (2.5, 2.9)	0.4 (0.1, 0.6)
Indonesia	0.3 (0.3, 0.4)	3.7 (3.6, 3.7)	0.8 (0.8, 0.9)
Laos	-0.8 (-0.9, -0.7)	3.0 (2.9, 3.1)	-1.2 (-1.3, -1.2)
Malaysia	-0.7 (-1.2, -0.2)	1.7 (1.1, 2.2)	-1.0 (-1.8, -0.2)
Myanmar	-1.7 (-1.7, -1.6)	1.9 (1.8, 2.0)	-3.2 (-3.2, -3.1)
Philippines	1.2 (0.7, 1.7)	4.3 (3.7, 5.0)	1.7 (1.4, 2.0)
Singapore	-3.9 (-4.2, -3.6)	-1.8 (-2.0, -1.6)	-5.1 (-5.4, -4.8)
Thailand	-2.3 (-2.7, -1.8)	1.2 (0.8, 1.7)	-3.1 (-3.6, -2.6)
Viet Nam	-0.9 (-0.9, -0.8)	3.4 (3.2, 3.7)	-0.3 (-0.4, -0.2)
**EEU**			
Armenia	-1.4 (-1.6, -1.1)	0.5 (0.3, 0.8)	-1.3 (-1.5, -1.0)
Belarus	-0.2 (-0.7, 0.2)	1.0 (0.6, 1.5)	0.1 (-0.6, 0.8)
Kazakhstan	-1.0 (-1.3, -0.6)	-0.2 (-0.6, 0.2)	-1.4 (-1.8, -0.9)
Kyrgyzstan	0.9 (0.2, 1.7)	1.3 (0.7, 1.9)	0.3 (-0.1, 0.8)
Russia	-1.2 (-2.1, -0.2)	0.2 (-0.8, 1.2)	-0.1 (-1.2, 1.1)
**Mercosur**			

Argentina	-2.6 (-2.9, -2.3)	-1.1 (-1.6, -0.6)	-3.0 (-3.3, -2.8)
Bolivia	-1.1 (-1.2, -0.9)	0.6 (0.5, 0.8)	-3.2 (-3.3, -3.1)
Brazil	-2.9 (-3.1, -2.7)	-0.9 (-1.1, -0.7)	-3.9 (-4.2, -3.7)
Paraguay	-0.8 (-1.2, -0.4)	0.6 (-0.1, 1.2)	-1.4 (-1.8, -0.9)
Uruguay	-2.9 (-3.3, -2.6)	-1.5 (-1.9, -1.2)	-2.8 (-3.1, -2.5)
Venezuela	-1.2 (-1.7, -0.6)	-0.5 (-1.4, 0.4)	-3.0 (-3.6, -2.4)

Note: AAPC, average annual percent change; ASMR, age-standardized mortality rate; IHD, ischemic heart diseases; BRICS, Brazil, Russia, India, China, and South Africa; SACU, South African Customs Union; SAARC, South Asian Association for Regional Cooperation; China-ASEAN FTA, China-ASEAN Free Trade Area; and EEU, Eurasian Economic Union.

Table S4: The average annual percent change in the burden of IS mortality for both sexes attributed to modifiable risk factors across BRICS-Plus from 1990 to 2019.

**Population**	**IS (ASMR/100,000)**		
	Dietary risks (AAPC (95%CI)	High BMI (AAPC (95%CI)	Smoking (AAPC (95%CI)
**BRICS-Plus**	-1.0 (-1.2, -0.9)	0.6 (0.3, 0.9)	-1.4 (-1.6, -1.3)
**SACU**			
Botswana	-0.3 (-0.4, -0.2)	2.4 (2.0, 2.7)	-0.9 (-1.0, -0.8)
Eswatini	-0.2 (-0.5, -0.1)	0.7 (0.3, 1.1)	-1.4 (-1.7, -1.2)
Lesotho	0.9 (0.8, 1.1)	3.2 (3.0, 3.4)	0.8 (0.6, 1.0)
Namibia	-0.7 (-0.8, -0.5)	0.9 (0.7, 1.1)	-1.7 (-1.8, -1.6)
South Africa	0.7 (-0.2, 1.5)	1.5 (0.5, 2.5)	-1.9 (-2.7, -1.1)
**SAARC**			
Afghanistan	0.8 (0.5, 1.2)	2.8 (2.6, 3.0)	2.0 (1.8, 2.3)
Bangladesh	0.1 (-0.6, 0.8)	3.9 (3.1, 4.6)	-1.2 (-1.9, -0.5)
Bhutan	-0.5 (-0.7, -0.3)	2.4 (1.9, 2.8)	-1.1 (-1.6, -0.6)
India	-1.4 (-2.2, -0.6)	2.3 (1.5, 3.1)	-1.9 (-2.7, -1.0)
Maldives	-2.4 (-2.8, -2.0)	0.6 (-0.1, 1.3)	-2.9 (-3.2, -2.7)
Nepal	-0.5 (-0.7, -0.3)	3.4 (2.7, 4.1)	-1.1 (-1.2, -1.0)
Pakistan	0.4 (0.4, 0.5)	3.1 (2.8, 3.4)	-1.0 (-1.1, -0.9)
Sri Lanka	-2.1 (-2.6, -1.7)	0.8 (0.2, 1.4)	-3.8 (-4.5, -3.1)

**China-ASEAN FTA**			
Brunei Darussalam	-2.6 (-3.1, -2.0)	-0.8 (-1.2, -0.3)	-4.4 (-5.1, -3.7)
Cambodia	-0.3 (-0.4, -0.2)	2.8 (2.1, 3.6)	-0.2 (-0.3, -0.1)
China	-0.5 (-0.9, -0.2)	1.9 (1.5, 2.2)	-0.1 (-0.5, 0.3)
Indonesia	0.6 (0.6, 0.7)	4.2 (3.9, 4.5)	1.5 (1.4, 1.6)
Laos	-0.5 (-0.6, -0.4)	3.3 (3.0, 3.6)	-0.8 (-0.9, -0.7)
Malaysia	-1.9 (-3.4, -0.3)	0.8 (0.1, 1.6)	-2.0 (-3.2, -0.9)
Myanmar	-0.8 (-0.9, -0.7)	3.2 (2.9, 3.5)	-2.5 (-2.6, -2.4)
Philippines	0.5 (0.1, 0.8)	3.9 (3.2, 4.6)	0.9 (0.5, 1.3)
Singapore	-5.5 (-6.0, -4.9)	-4.0 (-4.7, -3.3)	-7.5 (-8.5, -6.5)
Thailand	-2.1 (-2.8, -1.4)	1.4 (0.6, 2.1)	-3.3 (-4.1, -2.4)
Viet Nam	0.1 (-0.1, 0.1)	4.5 (3.9, 5.0)	0.4 (0.3, 0.5)
**EEU**			
Armenia	-2.5 (-3.4, -1.7)	-0.7 (-1.8, 0.3)	-2.7 (-3.8, -1.5)
Belarus	-1.1 (-1.6, -0.7)	-0.1 (-0.7, 0.5)	-0.8 (-1.4, -0.2)
Kazakhstan	-0.8 (-1.1, -0.5)	0.2 (-0.1, 0.5)	-0.5 (-0.8, -0.1)
Kyrgyzstan	-2.1 (-2.9, -1.3)	-0.9 (-1.5, -0.3)	-1.3 (-2.0, -0.5)
Russia	-1.9 (-2.5, -1.3)	-0.9 (-2.0, 0.2)	-0.6 (-1.6, 0.4)
**Mercosur**			

Argentina	-2.8 (-3.1, -2.4)	-1.4 (-1.8, -1.0)	-3.2 (-3.7, -2.8)
Bolivia	-1.0 (-1.2, -0.8)	0.6 (0.3, 0.9)	-3.3 (-3.6, -3.1)
Brazil	-3.0 (-3.2, -2.7)	-1.6 (-2.0, -1.3)	-5.0 (-5.3, -4.7)
Paraguay	-1.3 (-2.1, -0.5)	0.1 (-1.0, 1.2)	-2.1 (-2.9, -1.2)
Uruguay	-2.9 (-3.2, -2.5)	-1.8 (-2.1, -1.5)	-3.0 (-3.3, -2.7)
Venezuela	-1.0 (-1.8, -0.1)	-0.5 (-1.4, 0.4)	-3.2 (-4.1, -2.2)

Note: AAPC, average annual percent change; ASMR, age-standardized mortality rate; IS, ischemic stroke; BRICS, Brazil, Russia, India, China, and South Africa; SACU, South African Customs Union; SAARC, South Asian Association for Regional Cooperation; China-ASEAN FTA, China-ASEAN Free Trade Area; and EEU, Eurasian Economic Union.

Table S5: The average annual percent change in the burden of IHD ASMR attributed to modifiable risk factors in males and females across BRICS-Plus from 1990 to 2019.

**Population**	**Male (AAPC (95%CI)**			**Female (AAPC (95%CI)**		
	Dietary risks	High BMI	Smoking	Dietary risks	High BMI	Smoking
**BRICS-Plus**	-0.7 (-0.9, -0.6)	1.3 (1.0, 1.5)	-1.1 (-1.3, -0.9)	-0.9 (-1.0, -0.8)	0.7 (0.5, 0.8)	-1.8 (-1.9, -1.7)
**SACU**						
Botswana	0.2 (-0.1, 0.4)	3.9 (3.5, 4.2)	-0.3 (-0.6, -0.1)	0.4 (-0.3, 1.1)	2.4 (1.8, 3.1)	-0.3 (-1.3, 0.6)
Eswatini	-0.1 (-0.2, 0.1)	1.6 (1.3, 1.9)	-1.2 (-1.3, -1.0)	0.1 (-0.2, 0.3)	0.6 (0.3, 1.0)	-1.0 (-1.2, -0.8)
Lesotho	0.8 (0.7, 1.0)	4.0 (3.7, 4.4)	1.4 (1.1, 1.6)	1.4 (1.1, 1.6)	3.5 (3.2, 3.8)	1.4 (1.1, 1.7)
Namibia	-0.1 (-0.2, 0.1)	1.8 (1.7, 2.0)	-1.4 (-1.5, -1.2)	-0.5 (-0.7, -0.4)	0.6 (0.4, 0.8)	-1.7 (-1.8, -1.5)
South Africa	-0.2 (-0.8, 0.4)	1.1 (0.6, 1.7)	-2.1 (-2.7, -1.5)	-0.1 (-0.8, 0.6)	0.3 (-0.4, 1.1)	-2.8 (-3.6, -2.1)
**SAARC**						
Afghanistan	-1.0 (-1.1, -0.9)	1.0 (0.7, 1.2)	0.3 (0.2, 0.5)	-0.7 (-0.8, -0.6)	0.5 (0.2, 0.8)	0.7 (0.6, 0.9)
Bangladesh	0.1 (-0.4, 0.6)	4.3 (3.8, 4.8)	-0.6 (-1.0, -0.2)	0.1 (-0.4, 0.5)	3.8 (3.3, 4.2)	-1.3 (-1.7, -0.9)
Bhutan	0.1 (0.1, 0.2)	3.2 (3.1, 3.4)	-0.7 (-0.8, -0.6)	-0.3 (-0.4, -0.2)	2.3 (2.1, 2.5)	-1.3 (-1.5, -1.2)
India	-0.5 (-1.3, 0.3)	3.0 (2.6, 3.5)	-1.0 (-1.4, -0.7)	-0.5 (-1.4, 0.4)	2.7 (2.2, 3.1)	-0.6 (-1.1, -0.2)
Maldives	-2.7 (-3.0, -2.5)	0.5 (0.2, 0.8)	-3.4 (-3.7, -3.1)	-3.5 (-4.2, -2.8)	-1.1 (-1.8, -0.3)	-5.1 (-6.0, -4.2)
Nepal	0.7 (0.5, 0.8)	4.9 (4.7, 5.1)	0.2 (0.1, 0.3)	-0.5 (-0.6, -0.4)	3.3 (3.1, 3.5)	-1.3 (-1.5, -1.0)
Pakistan	1.3 (1.2, 1.4)	4.2 (4.0, 4.4)	0.5 (0.4, 0.6)	0.4 (0.4, 0.5)	3.0 (2.8, 3.2)	-0.6 (-0.8, -0.5)
Sri Lanka	-1.9 (-2.7, -1.1)	0.4 (-0.2, 1.1)	-3.4 (-3.9, -2.8)	-1.5 (-2.2, -0.8)	0.7 (-0.1, 1.4)	-3.7 (-4.2, -3.2)

**China-ASEAN FTA**						
Brunei Darussalam	-1.4 (-2.3, -0.4)	0.6 (-0.1, 1.3)	-3.0 (-3.8, -2.3)	-1.5 (-2.1, -0.9)	0.1 (-0.3, 0.4)	-3.5 (-3.8, -3.2)
Cambodia	-0.5 (-0.6, -0.4)	3.1 (3.0, 3.3)	-0.3 (-0.4, -0.3)	-0.7 (-0.8, -0.7)	2.1 (2.0, 2.2)	-0.8 (-0.9, -0.7)
China	0.4 (0.1, 0.6)	3.1 (2.8, 3.5)	0.3 (0.1, 0.6)	-0.3 (-0.5, -0.1)	2.2 (2.0, 2.5)	0.7 (0.5, 0.9)
Indonesia	0.5 (0.5, 0.6)	3.9 (3.8, 4.0)	0.9 (0.8, 0.9)	0.1 (-0.1, 0.1)	3.4 (3.2, 3.5)	0.8 (0.6, 0.9)
Laos	-0.9 (-1.0, -0.8)	3.4 (3.2, 3.5)	-1.2 (-1.3, -1.1)	-0.8 (-0.9, -0.7)	2.6 (2.5, 2.7)	-1.8 (-1.9, -1.7)
Malaysia	-0.7 (-1.4, 0.1)	2.0 (1.3, 2.6)	-1.0 (-1.8, -0.2)	-0.9 (-1.5, -0.2)	1.2 (0.6, 1.8)	-1.9 (-2.5, -1.2)
Myanmar	-1.0 (-1.2, -0.7)	2.8 (2.6, 3.0)	-2.3 (-2.4, -2.1)	-2.3 (-2.5, -2.2)	1.2 (1.1, 1.4)	-4.6 (-4.7, -4.5)
Philippines	1.6 (1.3, 1.8)	4.5 (3.7, 5.4)	1.9 (1.6, 2.2)	0.8 (0.3, 1.3)	4.1 (3.5, 4.7)	1.0 (0.7, 1.4)
Singapore	-3.8 (-4.3, -3.3)	-1.5 (-1.7, -1.3)	-5.0 (-5.2, -4.8)	-4.2 (-4.8, -3.6)	-2.4 (-2.8, -1.9)	-6.1 (-6.6, -5.7)
Thailand	-2.2 (-3.0, -1.5)	1.5 (1.0, 2.1)	-3.0 (-3.8, -2.2)	-2.5 (-2.9, -2.1)	0.7 (0.3, 1.1)	-4.8 (-5.3, -4.4)
Viet Nam	-0.5 (-0.5, -0.4)	4.3 (4.1, 4.6)	-0.2 (-0.3, -0.1)	-1.4 (-1.5, -1.4)	2.4 (2.2, 2.6)	-1.5 (-1.7, -1.2)
**EEU**						
Armenia	-1.2 (-1.5, -0.9)	1.2 (1.0, 1.5)	-1.3 (-1.5, -1.0)	-1.6 (-1.8, -1.3)	-0.1 (-0.5, 0.4)	-2.3 (-2.8, -1.9)
Belarus	0.1 (-0.4, 0.5)	1.7 (1.1, 2.2)	0.1 (-0.5, 0.7)	-0.5 (-1.0, -0.1)	0.5 (0.2, 0.9)	-0.4 (-1.0, 0.3)
Kazakhstan	-1.1 (-1.5, -0.7)	0.1 (-0.4, 0.5)	-1.5 (-1.9, -1.1)	-0.9 (-1.2, -0.6)	-0.4 (-0.7, -0.1)	-1.4 (-1.8, -1.0)
Kyrgyzstan	0.6 (-0.1, 1.4)	1.3 (0.8, 1.7)	0.1 (-0.4, 0.6)	0.9 (-0.1, 2.0)	1.3 (0.6, 2.0)	0.5 (-0.1, 1.0)
Russia	-1.3 (-2.3, -0.4)	0.4 (-0.7, 1.5)	-0.5 (-1.7, 0.6)	-1.3 (-2.2, -0.4)	-0.1 (-1.0, 0.8)	0.6 (-0.3, 1.5)

**Mercosur**						
Argentina	-2.6 (-2.9, -2.3)	-1.1 (-1.5, -0.6)	-3.1 (-3.4, -2.9)	-2.6 (-3.0, -2.3)	-1.2 (-1.7, -0.7)	-2.8 (-3.1, 2.5)
Bolivia	-1.3 (-1.4, -1.1)	0.7 (0.6, 0.9)	-3.2 (-3.3, -3.1)	-1.0 (-1.2, -0.8)	0.5 (0.4, 0.6)	-3.6 (-3.9, -3.4)
Brazil	-2.7 (-2.8, -2.5)	-0.6 (-0.8, -0.4)	-3.8 (-4.1, -3.5)	-3.1 (-3.3, -2.9)	-1.3 (-1.6, -1.0)	-4.0 (-4.3, -3.8)
Paraguay	-0.5 (-0.9, -0.1)	1.1 (0.6, 1.6)	-1.1 (-1.5, -0.6)	-1.3 (-1.8, -0.9)	-0.1 (-0.6, 0.3)	-2.1 (-2.6, -1.6)
Uruguay	-2.8 (-3.2, -2.4)	-1.4 (-1.8, -1.0)	-2.8 (-3.4, -2.3)	-3.2 (-3.7, -2.7)	-1.8 (-2.2, -1.4)	-2.8 (-3.1, -2.5)
Venezuela	-0.9 (-1.7, -0.1)	-0.1 (-1.3, 1.1)	-2.8 (-3.5, -2.0)	-1.6 (-2.0, -1.2)	-0.8 (-1.2, -0.4)	-3.4 (-3.8, -3.0)

Note: AAPC, average annual percent change; ASMR, age-standardized mortality rate; IHD, ischemic heart diseases; BRICS, Brazil, Russia, India, China, and South Africa; SACU, South African Customs Union; SAARC, South Asian Association for Regional Cooperation; China-ASEAN FTA, China-ASEAN Free Trade Area; and EEU, Eurasian Economic Union.

Table S6: The average annual percent change in the burden of IS ASMR attributed to modifiable risk factors in males and females across BRICS-Plus from 1990 to 2019.

**Population**	**Male (AAPC (95%CI)**			**Female (AAPC (95%CI)**		
	Dietary risks	High BMI	Smoking	Dietary risks	High BMI	Smoking
**BRICS-Plus**	-1.0 (-1.1, -0.8)	1.0 (0.6, 1.4)	-1.3 (-1.4, -1.1)	-1.1 (-1.2, -0.9)	0.4 (0.2, 0.6)	-2.0 (-2.4, -1.5)
**SACU**						
Botswana	-0.3 (-0.5, -0.1)	3.2 (2.8, 3.6)	-0.6 (-1.2, -0.1)	-0.4 (-1.2, 0.5)	2.0 (1.0, 3.0)	-1.1 (-1.8, -0.4)
Eswatini	-0.2 (-0.3, -0.1)	1.3 (1.1, 1.5)	-1.6 (-1.9, -1.3)	-0.2 (-0.5, -0.1)	0.2 (-0.2, 0.6)	-1.2 (-1.5, -0.9)
Lesotho	0.5 (0.3, 0.7)	3.5 (3.2, 3.9)	0.8 (0.6, 1.1)	1.1 (0.9, 1.3)	3.0 (2.7, 3.2)	1.1 (0.6, 1.5)
Namibia	-0.4 (-0.5, -0.3)	1.6 (1.4, 1.7)	-1.5 (-1.6, -1.4)	-0.8 (-1.0, -0.7)	0.5 (0.3, 0.7)	-1.8 (-1.9, -1.7)
South Africa	0.4 (-0.1, 0.8)	1.8 (1.1, 2.5)	-1.8 (-2.6, -0.9)	0.6 (-0.1, 1.3)	1.0 (-0.1, 2.0)	-2.3 (-3.0, -1.6)
**SAARC**						
Afghanistan	0.6 (0.3, 0.9)	2.9 (2.7, 3.2)	2.3 (2.1, 2.5)	1.0 (0.6, 1.3)	2.5 (2.2, 2.8)	2.5 (1.8, 3.3)
Bangladesh	0.2 (-0.6, 0.9)	4.1 (3.2, 4.9)	-1.0 (-1.6, -0.4)	-0.1 (-1.3, 1.2)	3.1 (2.5, 3.7)	-1.6 (-2.4, -0.8)
Bhutan	-0.5 (-0.6, -0.3)	2.7 (2.1, 3.3)	-1.2 (-1.5, -1.0)	-0.6 (-0.8, -0.5)	2.1 (1.7, 2.6)	-1.5 (-2.1, -1.0)
India	-1.4 (-2.2, -0.6)	2.2 (1.4, 3.0)	-1.8 (-2.4, -1.2)	-1.3 (-2.2, -0.5)	2.2 (1.8, 2.7)	-1.1 (-1.9, -0.3)
Maldives	-2.1 (-2.3, -1.8)	1.5 (0.9, 2.1)	-2.3 (-2.5, -2.1)	-3.0 (-3.9, -2.0)	-0.4 (-1.6, 0.9)	-4.0 (-5.1, -2.9)
Nepal	-0.2 (-0.3, -0.1)	4.3 (3.8, 4.9)	-0.6 (-0.7, -0.5)	-1.1 (-1.2, -0.9)	2.5 (1.6, 3.4)	-1.7 (-2.0, -1.5)
Pakistan	0.3 (02, 0.3)	3.1 (2.8, 3.5)	-0.8 (-0.9, -0.7)	0.6 (0.5, 0.7)	3.0 (2.7, 3.3)	-0.6 (-0.8, -0.4)
Sri Lanka	-2.0 (-2.4, -1.5)	1.0 (0.5, 1.5)	-3.4 (-4.1, -2.7)	-2.0 (-2.6, -1.5)	0.8 (0.3, 1.3)	-4.2 (-5.5, -2.9)

**China-ASEAN FTA**						
Brunei Darussalam	-2.4 (-3.4, -1.5)	-0.9 (-1.4, -0.3)	-4.4 (-5.3, -3.4)	-2.4 (-2.9, -1.9)	-0.8 (-1.2, -0.4)	-4.3 (-4.6, -3.9)
Cambodia	-0.2 (-0.3, -0.1)	3.3 (2.7, 3.9)	0.1 (-0.1, 0.1)	-0.3 (-0.4, -0.3)	2.6 (1.9, 3.2)	-0.3 (-0.5, -0.1)
China	-0.1 (-0.7, 0.5)	2.5 (1.7, 3.2)	-0.1 (-0.4, 0.2)	-1.1 (-1.6, -0.7)	1.3 (1.0, 1.6)	-0.2 (-0.6, 0.2)
Indonesia	0.8 (0.7, 0.8)	4.5 (4.2, 4.8)	1.6 (1.5, 1.7)	0.4 (0.4, 0.5)	3.9 (3.7, 4.2)	1.7 (1.4, 2.1)
Laos	-0.6 (-0.7, -0.5)	3.6 (3.3, 3.9)	-0.8 (-0.9, -0.7)	-0.5 (-0.6, -0.4)	3.0 (2.7, 3.2)	-1.5 (-1.7, -1.3)
Malaysia	-1.7 (-3.0, -0.3)	1.1 (0.3, 1.8)	-1.9 (-3.0, -0.8)	-1.6 (-3.0, -0.3)	0.5 (-0.3, 1.3)	-2.0 (-3.4, -0.6)
Myanmar	-1.0 (-1.1, -1.0)	2.9 (2.7, 3.2)	-2.3 (-2.3, -2.2)	-0.4 (-0.5, -0.2)	3.6 (3.2, 4.0)	-2.7 (-2.8, -2.6)
Philippines	1.1 (0.6, 1.5)	4.4 (3.6, 5.2)	1.3 (1.0, 1.7)	-0.1 (-0.8, 0.6)	3.5 (2.9, 4.1)	0.1 (-0.5, 0.6)
Singapore	-5.5 (-6.1, -5.0)	-3.8 (-4.5, -3.2)	-7.4 (-8.2, -6.6)	-5.4 (-6.1, -4.7)	-4.3 (-4.9, -3.7)	-7.5 (-8.4, -6.6)
Thailand	-2.2 (-3.0, -1.4)	1.7 (0.7, 2.6)	-3.1 (-3.9, -2.3)	-2.0 (-2.5, -1.5)	1.3 (0.7, 1.8)	-4.2 (-4.9, -3.5)
Viet Nam	0.3 (0.2, 0.4)	5.1 (4.7, 5.6)	0.4 (0.4, 0.5)	-0.2 (-0.3, -0.1)	3.7 (3.2, 4.1)	-0.3 (-1.0, 0.4)
**EEU**						
Armenia	-2.6 (-3.5, -1.7)	-0.2 (-1.1, 0.7)	-2.5 (-3.6, -1.4)	-2.6 (-3.3, -1.9)	-1.2 (-1.9, -0.5)	-3.3 (-4.4, -2.2)
Belarus	-0.8 (-1.3, -0.3)	0.8 (0.1, 0.5)	-0.7 (-1.3, -0.2)	-1.4 (-1.8, -1.0)	-0.7 (-1.2, -0.2)	-1.8 (-2.4, -1.3)
Kazakhstan	-0.8 (-1.1, -0.4)	0.8 (0.5, 1.2)	-0.7 (-1.0, -0.3)	-0.8 (-1.1, -0.5)	-0.3 (-0.6, 0.1)	-0.9 (-1.6, -0.3)
Kyrgyzstan	-1.9 (-2.7, -1.0)	-0.3 (-0.7, 0.2)	-1.6 (-2.3, -0.9)	-2.5 (-3.4, -1.6)	-1.5 (-2.0, -1.0)	-1.7 (-2.7, -0.7)
Russia	-1.9 (-2.6, -1.2)	-0.1 (-1.3, 1.0)	-1.1 (-2.2, -0.1)	-2.1 (-2.5, -1.6)	-1.2 (-1.8, -0.6)	-0.6 (-2.2, 1.1)

**Mercosur**						
Argentina	-2.7 (-3.1, -2.4)	-1.2 (-1.7, -0.8)	-3.5 (-4.0, -3.0)	-2.7 (-3.5, -2.0)	-1.4 (-1.9, -0.8)	-3.1 (-3.6, -2.6)
Bolivia	-1.1 (-1.3, -0.9)	0.7 (0.3, 1.1)	-3.3 (-3.6, -3.1)	-0.9 (-1.0, -0.7)	0.6 (0.4, 0.8)	-3.4 (-4.4, -2.4)
Brazil	-2.8 (-3.2, -2.5)	-1.4 (-1.8, -1.1)	-4.8 (-5.1, -4.5)	-3.1 (-3.4, -2.9)	-2.0 (-2.2, -1.8)	-5.2 (-5.5, -4.9)
Paraguay	-1.2 (-2.0, -0.4)	0.4 (-0.2, 1.1)	-1.9 (-2.8, -1.0)	-1.5 (-2.3, -0.7)	-0.5 (-1.5, 0.6)	-2.3 (-2.9, -1.6)
Uruguay	-2.8 (-3.4, -2.2)	-1.6 (-2.0, -1.3)	-3.2 (-3.4, -2.9)	-3.0 (-3.4, -2.5)	-2.0 (-2.4, -1.5)	-2.9 (-3.3, -2.5)
Venezuela	-0.8 (-1.8, 0.2)	-0.1 (-1.3, 1.1)	-3.2 (-4.0, -2.3)	-1.4 (-1.7, -1.0)	-0.9 (-1.8, -0.1)	-3.5 (-4.0, -2.9)

Note: AAPC, average annual percent change; ASMR, age-standardized mortality rate; IS, ischemic stroke; BRICS, Brazil, Russia, India, China, and South Africa; SACU, South African Customs Union; SAARC, South Asian Association for Regional Cooperation; China-ASEAN FTA, China-ASEAN Free Trade Area; and EEU, Eurasian Economic Union.

Table S7: The average annual percent change in the age-specific (25-49 years) CVD mortality for both sexes attributed to modifiable risk factors across BRICS-Plus from 1990 to 2019.

**Population**	**CVD (25-49 years)/100,000**		
	Dietary risks (AAPC (95%CI)	High BMI (AAPC (95%CI)	Smoking (AAPC (95%CI)
**BRICS-Plus**	-1.0 (-1.2, -0.8)	0.8 (0.5, 1.1)	-1.0 (-1.3, -0.7)
**SACU**			
Botswana	-0.1 (-0.5, 0.4)	2.0 (1.4, 2.6)	-0.5 (-1.2, 0.1)
Eswatini	0.2 (-0.5, 0.9)	0.7 (0.3, 1.1)	-0.6 (-1.2, 0.1)
Lesotho	2.0 (1.7, 2.3)	3.6 (3.2, 4.0)	2.4 (1.8, 2.9)
Namibia	-0.7 (-1.0, -0.3)	-0.1 (-0.4, 0.3)	-1.4 (-1.8, -1.0)
South Africa	-2.7 (-3.5, -1.9)	-2.2 (-3.2, -1.2)	-3.9 (-5.0, -2.8)
**SAARC**			
Afghanistan	-1.7 (-2.1, -1.2)	-0.4 (-0.7, -0.1)	0.6 (0.3, 0.9)
Bangladesh	-1.0 (-1.5, -0.4)	3.8 (3.4, 4.1)	-1.2 (-1.8, -0.7)
Bhutan	-1.2 (-1.3, -1.0)	1.4 (1.2, 1.6)	-1.7 (-1.9, -1.5)
India	-0.5 (-1.3, 0.3)	2.6 (2.0, 3.3)	-0.9 (-1.6, -0.1)
Maldives	-4.1 (-4.5, -3.7)	-1.1 (-1.5, -0.7)	-3.6 (-4.0, -3.2)
Nepal	-1.4 (-1.5, -1.3)	2.9 (2.7, 3.1)	-1.8 (-1.9, -1.6)
Pakistan	1.1 (0.9, 1.2)	3.2 (3.1, 3.3)	0.2 (0.1, 0.3)
Sri Lanka	-2.0 (-3.1, -0.9)	0.2 (-0.8, 1.2)	-3.1 (-3.9, -2.4)

**China-ASEAN FTA**			
Brunei Darussalam	-1.1 (-1.6, -0.6)	0.6 (-0.4, 1.5)	-1.7 (-2.3, -1.1)
Cambodia	-1.7 (-1.9, -1.4)	1.5 (1.3, 1.7)	-1.1 (-1.2, -0.9)
China	-0.9 (-1.3, -0.6)	1.7 (1.3, 2.2)	0.1 (-0.4, 0.4)
Indonesia	-0.4 (-0.5, -0.3)	2.7 (2.6, 2.9)	0.7 (0.5, 0.8)
Laos	-1.3 (-1.5, -1.1)	3.0 (2.8, 3.2)	-1.3 (-1.5, -1.2)
Malaysia	-0.5 (-1.1, 0.2)	0.8 (-0.2, 1.8)	-0.1 (-1.1, 0.9)
Myanmar	-2.5 (-2.6, -2.4)	1.7 (1.5, 2.0)	-3.2 (-3.4, -3.1)
Philippines	3.8 (3.4, 4.3)	6.2 (5.2, 7.1)	3.5 (3.0, 3.9)
Singapore	-3.0 (-3.6, -2.4)	-0.5 (-1.0, 0.1)	-3.9 (-4.8, -3.0)
Thailand	-0.5 (-1.1, 0.1)	2.4 (1.7, 3.0)	-0.6 (-1.2, 0.1)
Viet Nam	-0.6 (-0.7, -0.4)	3.9 (3.5, 4.2)	0.5 (0.4, 0.6)
**EEU**			
Armenia	-0.9 (-2.0, 0.1)	1.0 (-0.1, 2.0)	-0.6 (-1.7, 0.5)
Belarus	-0.3 (-1.6, 0.9)	0.8 (-0.5, 2.0)	-0.2 (-1.4, 1.0)
Kazakhstan	-1.5 (-2.2, -0.7)	0.1 (-0.6, 0.6)	-1.2 (-2.0, -0.4)
Kyrgyzstan	-1.6 (-2.6, -0.5)	-0.6 (-1.6, 0.5)	-1.3 (-2.4, -0.2)
Russia	-0.7 (-3.1, 1.7)	0.8 (-0.9, 2.4)	-0.1 (-1.9, 1.6)
**Mercosur**			

Argentina	-3.2 (-3.4, -3.0)	-1.9 (-2.1, -1.7)	-3.6 (-3.9, -3.4)
Bolivia	-2.2 (-2.4, -2.0)	-1.0 (-1.2, -0.9)	-4.1 (-4.4, -3.8)
Brazil	-2.6 (-2.8, -2.4)	-1.3 (-1.6, -1.1)	-4.0 (-4.2, -3.7)
Paraguay	-1.4 (-2.6, -0.2)	-0.4 (-1.1, 0.2)	-1.8 (-2.6, -0.9)
Uruguay	-2.9 (-3.2, -2.5)	-1.3 (-1.7, -0.8)	-2.7 (-3.0, -2.5)
Venezuela	-0.7 (-1.2, -0.1)	-0.1 (-0.6, 0.3)	-1.9 (-2.5, -1.2)

Note: AAPC, average annual percent change; CVD, cardiovascular diseases; BRICS, Brazil, Russia, India, China, and South Africa; SACU, South African Customs Union; SAARC, South Asian Association for Regional Cooperation; China-ASEAN FTA, China-ASEAN Free Trade Area; and EEU, Eurasian Economic Union.

Table S8: The average annual percent change in the age-specific (25-49 years) IHD mortality for both sexes attributed to modifiable risk factors across BRICS-Plus from 1990 to 2019.

**Population**	**IHD (25-49 years)/100,000**		
	Dietary risks (AAPC (95%CI)	High BMI (AAPC (95%CI)	Smoking (AAPC (95%CI)
**BRICS-Plus**	-0.8 (-1.0, -0.5)	1.0 (0.5, 1.5)	-0.8 (-1.2, -0.5)
**SACU**			
Botswana	0.5 (0.1, 0.9)	3.7 (3.4, 4.1)	-0.1 (-1.1, 1.0)
Eswatini	0.8 (0.2, 1.2)	1.6 (0.9, 2.3)	-0.2 (-0.6, 0.3)
Lesotho	2.6 (2.3, 3.0)	5.0 (4.6, 5.5)	2.9 (2.5, 3.3)
Namibia	0.1 (-0.3, 0.4)	1.2 (0.9, 1.6)	-0.9 (-1.3, -0.5)
South Africa	-2.2 (-3.1, -1.3)	-1.5 (-2.5, -0.5)	-3.5 (-4.7, -2.3)
**SAARC**			
Afghanistan	-1.6 (-1.9, -1.3)	-0.3 (-0.6, 0.1)	0.5 (0.2, 0.8)
Bangladesh	-0.7 (-1.1, -0.3)	4.3 (3.9, 4.8)	-0.9 (-1.4, -0.4)
Bhutan	-0.9 (-1.0, -0.7)	2.2 (2.0, 2.4)	-1.4 (-1.6, -1.1)
India	-0.3 (-1.0, 0.4)	3.2 (2.6, 3.8)	-0.8 (-1.5, -0.1)
Maldives	-3.8 (-4.1, -3.4)	-0.2 (-0.9, 0.5)	-3.4 (-3.8, -3.0)
Nepal	-1.1 (-1.2, -0.9)	3.7 (3.4, 4.0)	-1.5 (-1.6, -1.3)
Pakistan	1.3 (1.2, 1.4)	3.9 (3.7, 4.1)	0.5 (0.4, 0.7)
Sri Lanka	-2.2 (-3.6, -0.7)	0.2 (-1.4, 1.7)	-3.3 (-4.1, -2.5)

**China-ASEAN FTA**			
Brunei Darussalam	-0.6 (-1.2, -0.1)	1.4 (0.5, 2.3)	-1.2 (-1.9, -0.6)
Cambodia	-1.4 (-1.5, -1.3)	2.2 (1.8, 2.6)	-0.8 (-1.0, -0.7)
China	-0.1 (-0.4, 0.2)	3.1 (2.6, 3.6)	0.6 (0.3, 0.9)
Indonesia	0.1 (-0.1, 0.3)	3.5 (3.3, 3.7)	0.9 (0.7, 1.1)
Laos	-1.0 (-1.2, -0.9)	3.4 (3.1, 3.7)	-1.2 (-1.4, -1.1)
Malaysia	0.7 (0.1, 1.3)	2.7 (2.1, 3.4)	0.9 (0.2, 1.5)
Myanmar	-2.6 (-2.8, -2.5)	1.2 (0.9, 1.4)	-3.0 (-3.1, -2.9)
Philippines	3.8 (3.4, 4.2)	6.2 (5.3, 7.2)	3.4 (3.0, 3.8)
Singapore	-2.7 (-3.3, -2.0)	-0.3 (-0.8, 0.3)	-3.4 (-4.3, -2.5)
Thailand	-0.7 (-1.2, -0.2)	2.0 (1.5, 2.6)	-0.8 (-1.4, -0.2)
Viet Nam	-0.8 (-1.0, -0.7)	3.4 (2.8, 3.9)	0.3 (0.1, 0.4)
**EEU**			
Armenia	-0.8 (-1.7, 0.2)	1.3 (0.5, 2.2)	-0.7 (-1.7, 0.3)
Belarus	-0.3 (-1.5, 1.0)	0.9 (-0.4, 2.2)	-0.2 (-1.5, 1.1)
Kazakhstan	-2.0 (-2.8, -1.2)	-0.8 (-1.5, 0.1)	-2.0 (-2.8, -1.1)
Kyrgyzstan	-1.4 (-2.4, -0.3)	-0.4 (-1.5, 0.6)	-1.5 (-2.6, -0.4)
Russia	-1.1 (-3.0, 0.8)	0.3 (-1.4, 2.1)	-0.6 (-2.4, 1.2)
**Mercosur**			

Argentina	-3.0 (-3.2, -2.7)	-1.4 (-1.7, -1.2)	-3.4 (-3.6, -3.2)
Bolivia	-2.0 (-2.1, -1.8)	-0.4 (-0.6, -0.1)	-4.0 (-4.4, -3.5)
Brazil	-2.2 (-2.6, -1.8)	-0.4 (-0.7, -0.1)	-3.5 (-3.8, -3.3)
Paraguay	-1.0 (-2.4, 0.4)	0.2 (-1.0, 1.4)	-1.4 (-2.3, -0.4)
Uruguay	-2.7 (-3.2, -2.3)	-1.3 (-2.1, -0.50	-2.8 (-3.3, -2.4)
Venezuela	-0.6 (-1.4, 0.3)	0.2 (-0.8, 1.2)	-1.7 (-2.5, -1.0)

Note: AAPC, average annual percent change; IHD, ischemic heart diseases; BRICS, Brazil, Russia, India, China, and South Africa; SACU, South African Customs Union; SAARC, South Asian Association for Regional Cooperation; China-ASEAN FTA, China-ASEAN Free Trade Area; and EEU, Eurasian Economic Union.

Table S9: The average annual percent change in the age-specific (25-49 years) IS mortality for both sexes attributed to modifiable risk factors across BRICS-Plus from 1990 to 2019.

**Population**	**IS (25-49 years)/100,000**		
	Dietary risks (AAPC (95%CI)	High BMI (AAPC (95%CI)	Smoking (AAPC (95%CI)
**BRICS-Plus**	-0.5 (-1.2, 0.2)	1.0 (0.1, 1.8)	-0.5 (-1.3, 0.2)
**SACU**			
Botswana	-0.1 (-0.9, 0.8)	2.2 (1.2, 3.2)	-0.2 (-1.7, 1.4)
Eswatini	-0.1 (-0.8, 0.7)	0.5 (-0.7, 1.8)	0.1 (-2.9, 3.1)
Lesotho	1.7 (0.2, 3.1)	4.5 (2.5, 6.4)	1.4 (1.4, 1.5)
Namibia	-1.1 (-2.4, 0.2)	-0.1 (-2.1, 2.0)	-2.3 (-2.3, -2.2)
South Africa	-2.8 (-4.2, -1.3)	-2.4 (-3.8, -0.9)	-4.3 (-6.6, -1.9)
**SAARC**			
Afghanistan	1.6 (1.0, 2.2)	2.6 (2.0, 3.2)	3.8 (2.4, 5.2)
Bangladesh	-0.8 (-0.8, -0.7)	4.9 (2.0, 7.8)	-1.0 (-2.7, 0.7)
Bhutan	-1.1 (-3.6, 1.5)	2.0 (2.0, 2.1)	-0.1 (-0.1, -0.1)
India	-0.9 (-2.7, 1.0)	4.5 (0.6, 8.5)	-0.1 (-0.1, -0.1)
Maldives	-4.3 (-5.4, -3.1)	0.3 (-1.9, 2.5)	-3.7 (-5.0, -2.3)
Nepal	-1.9 (-3.4, -0.5)	2.4 (2.4, 2.5)	-2.3 (-2.3, -2.2)
Pakistan	1.2 (0.2, 2.2)	4.3 (2.5, 6.2)	0.3 (-1.3, 1.8)
Sri Lanka	-0.6 (-1.6, 0.4)	1.9 (-1.5, 5.4)	-2.4 (-7.0, 2.5)

**China-ASEAN FTA**			
Brunei Darussalam	-2.7 (-4.1, -1.3)	-0.1 (-6.2, 6.5)	-4.1 (-10.5, 2.8)
Cambodia	-1.2 (-1.9, -0.5)	1.4 (1.4, 1.5)	-0.1 (-1.2, 1.2)
China	-0.2 (-0.6, 0.3)	2.6 (1.2, 4.2)	0.5 (-0.6, 1.7)
Indonesia	0.3 (0.2, 0.3)	3.4 (2.5, 4.3)	1.6 (0.5, 2.8)
Laos	-0.7 (-1.3, -0.1)	3.2 (1.6, 4.9)	-1.2 (-1.2, -1.1)
Malaysia	-1.1 (-1.9, -0.3)	0.9 (-0.6, 2.4)	-1.0 (-3.5, 1.6)
Myanmar	-1.4 (-1.9, -0.9)	2.4 (1.6, 3.2)	-2.6 (-3.5, -1.6)
Philippines	3.7 (3.1, 4.3)	7.5 (5.3, 9.8)	3.8 (2.6, 5.1)
Singapore	-5.8 (-9.1, -2.4)	-2.3 (-2.3, -2.2)	-7.5 (-9.0, -6.0)
Thailand	0.8 (-0.7, 2.5)	3.9 (2.2, 5.6)	0.1 (-2.0, 2.1)
Viet Nam	1.0 (0.5, 1.5)	5.7 (3.4, 8.0)	1.9 (0.9, 2.8)
**EEU**			
Armenia	-2.4 (-4.2, -0.5)	-0.6 (-2.1, 0.9)	-1.6 (-3.8, 0.6)
Belarus	-0.7 (-2.0, 0.7)	0.5 (-0.9, 1.8)	-0.4 (-1.6, 0.8)
Kazakhstan	-0.9 (-1.6, -0.2)	0.5 (-0.5, 1.4)	-0.8 (-2.3, 0.8)
Kyrgyzstan	-1.5 (-2.5, -0.4)	-0.3 (-1.2, 0.7)	-0.6 (-2.4, 1.3)
Russia	-0.5 (-2.5, 1.5)	0.7 (-1.0, 2.4)	0.4 (-1.5, 2.4)
**Mercosur**			

Argentina	-4.9 (-6.9, -2.8)	-2.3 (-3.5, -1.2)	-5.6 (-8.7, -2.3)
Bolivia	-2.6 (-3.8, -1.3)	-0.3 (-1.4, 0.7)	-2.3 (-2.3, -2.2)
Brazil	-4.0 (-4.7, -3.2)	-2.9 (-3.8, -2.0)	-6.2 (-8.3, -4.1)
Paraguay	-1.5 (-3.2, 0.2)	0.1 (-2.2, 2.5)	-1.4 (-1.4, -1.3)
Uruguay	-5.3 (-6.6, -4.0)	-3.2 (-4.2, -2.2)	-5.6 (-7.3, -3.9)
Venezuela	-2.5 (-2.5, -2.4)	-1.4 (-1.4, -1.3)	-2.3 (-2.3, -2.2)

Note: AAPC, average annual percent change; IS, ischemic stroke; BRICS, Brazil, Russia, India, China, and South Africa; SACU, South African Customs Union; SAARC, South Asian Association for Regional Cooperation; China-ASEAN FTA, China-ASEAN Free Trade Area; and EEU, Eurasian Economic Union.

Table S10: The average annual percent change in the age-specific (50-69 years) CVD mortality for both sexes attributed to modifiable risk factors across BRICS-Plus from 1990 to 2019.

Population	CVD (50-69 years)/100,000		
	Dietary risks (AAPC (95%CI)	High BMI (AAPC (95%CI)	Smoking (AAPC (95%CI)
**BRICS-Plus**	-1.3 (-1.4, -1.2)	0.6 (0.5, 0.7)	-1.5 (-1.6, -1.3)
**SACU**			
Botswana	-0.6 (-0.9, -0.4)	1.5 (1.2, 1.7)	-1.1 (-1.4, -0.9)
Eswatini	-0.6 (-0.9, -0.2)	0.5 (-0.1, 1.0)	-1.7 (-2.0, -1.5)
Lesotho	0.9 (0.7, 1.0)	2.9 (2.7, 3.1)	0.9 (0.7, 1.0)
Namibia	-1.4 (-1.6, -1.3)	-0.1 (-0.3, 0.1)	-2.5 (-2.6, -2.3)
South Africa	-0.6 (-1.4, 0.2)	0.1 (-0.6, 0.8)	-2.3 (-2.9, -1.7)
**SAARC**			
Afghanistan	-1.6 (-1.7, -1.5)	0.1 (-0.2, 0.4)	0.1 (-0.1, 0.2)
Bangladesh	-0.7 (-1.1, -0.4)	3.5 (3.2, 3.9)	-1.2 (-1.6, -0.8)
Bhutan	-1.0 (-1.1, -0.9)	1.6 (1.5, 1.7)	-1.5 (-1.6, -1.4)
India	-0.7 (-1.1, -0.3)	2.6 (2.0, 3.2)	-1.4 (-1.8, -0.9)
Maldives	-4.6 (-5.2, -4.1)	-0.9 (-1.3, -0.6)	-4.6 (-5.0, -4.1)
Nepal	-0.7 (-0.7, -0.6)	3.4 (3.3, 3.5)	-1.1 (-1.2, -1.0)
Pakistan	0.6 (0.5, 0.7)	3.1 (3.0, 3.2)	-0.2 (-0.3, -0.1)
Sri Lanka	-2.2 (-2.7, -1.7)	0.1 (-0.3, 0.5)	-3.3 (-4.0, -2.6)

**China-ASEAN FTA**			
Brunei Darussalam	-2.7 (-3.0, -2.3)	-0.8 (-1.1, -0.4)	-3.9 (-4.2, -3.5)
Cambodia	-1.7 (-1.8, -1.6)	1.7 (1.5, 1.8)	-1.2 (-1.3, -1.1)
China	-2.1 (-2.7, -1.5)	0.3 (-0.2, 0.9)	-1.5 (-1.7, -1.2)
Indonesia	-0.6 (-0.6, -0.5)	3.5 (3.4, 3.6)	0.6 (0.5, 0.6)
Laos	-1.9 (-2.0, -1.8)	2.6 (2.5, 2.8)	-1.7 (-1.8, -1.6)
Malaysia	-1.7 (-1.9, -1.5)	0.1 (-0.5, 0.7)	-1.6 (-2.2, -0.9)
Myanmar	-2.5 (-2.6, -2.4)	2.0 (1.8, 2.1)	-3.5 (-3.6, -3.4)
Philippines	2.3 (1.8, 2.8)	4.8 (4.2, 5.3)	2.3 (1.9, 2.6)
Singapore	-4.8 (-5.1, -4.5)	-2.2 (-2.3, -2.0)	-5.5 (-5.7, -5.3)
Thailand	-2.8 (-3.6, -2.0)	0.5 (-0.2, 1.2)	-3.1 (-4.0, -2.3)
Viet Nam	-1.5 (-1.6, -1.5)	3.0 (2.9, 3.2)	-0.5 (-0.5, -0.4)
**EEU**			
Armenia	-1.3 (-1.8, -0.7)	0.2 (-0.5, 0.8)	-1.0 (-1.7, -0.3)
Belarus	0.1 (-0.7, 0.7)	0.7 (0.1, 1.4)	0.3 (-0.4, 1.0)
Kazakhstan	-1.6 (-2.1, -1.1)	-0.2 (-0.6, 0.3)	-1.1 (-1.6, -0.7)
Kyrgyzstan	-0.9 (-1.5, -0.2)	-0.2 (-0.9, 0.5)	-0.4 (-1.2, 0.3)
Russia	-0.9 (-1.9, 0.1)	0.1 (-0.8, 1.1)	-0.1 (-1.6, 1.4)
**Mercosur**			

Argentina	-2.6 (-3.1, -2.2)	-1.3 (-1.7, -0.9)	-3.0 (-3.4, -2.6)
Bolivia	-1.7 (-1.9, -1.6)	-0.6 (-0.7, -0.5)	-3.4 (-3.6, -3.3)
Brazil	-2.7 (-2.9, -2.6)	-1.4 (-1.6, -1.1)	-3.7 (-4.0, -3.5)
Paraguay	-1.1 (-2.0, -0.2)	-0.3 (-1.3, 0.7)	-1.7 (-2.5, -0.9)
Uruguay	-2.8 (-3.2, -2.4)	-1.3 (-1.7, -1.0)	-2.4 (-2.7, -2.1)
Venezuela	-1.3 (-1.8, -0.7)	-0.7 (-1.3, -0.1)	-2.9 (-3.5, -2.2)

Note: AAPC, average annual percent change; CVD, cardiovascular diseases; BRICS, Brazil, Russia, India, China, and South Africa; SACU, South African Customs Union; SAARC, South Asian Association for Regional Cooperation; China-ASEAN FTA, China-ASEAN Free Trade Area; and EEU, Eurasian Economic Union.

Table S11: The average annual percent change in the age-specific (50-69 years) IHD mortality for both sexes attributed to modifiable risk factors across BRICS-Plus from 1990 to 2019.

**Population**	**IHD (50-69 years)/100,000**		
	Dietary risks (AAPC (95%CI)	High BMI (AAPC (95%CI)	Smoking (AAPC (95%CI)
**BRICS-Plus**	-1.1 (-1.2, -1.0)	0.9 (0.7, 1.0)	-1.2 (-1.4, -1.1)
**SACU**			
Botswana	-0.1 (-0.2, 0.1)	2.6 (2.5, 2.8)	-0.6 (-0.8, -0.4)
Eswatini	-0.2 (-0.5, 0.2)	1.1 (0.7, 1.4)	-1.4 (-1.7, -1.2)
Lesotho	1.2 (1.1, 1.4)	3.8 (3.6, 4.0)	1.2 (1.0, 1.4)
Namibia	-0.9 (-1.1, -0.8)	0.8 (0.6, 1.0)	-2.0 (-2.2, -1.8)
South Africa	-0.5 (-1.3, 0.3)	0.3 (-0.5, 1.1)	-2.1 (-2.8, -1.5)
**SAARC**			
Afghanistan	-1.7 (-1.8, -1.6)	0.1 (-0.2, 0.4)	-0.1 (-0.1, 0.1)
Bangladesh	-0.1 (-0.4, 0.2)	4.6 (4.3, 4.9)	-0.9 (-1.4, -0.4)
Bhutan	-0.6 (-0.7, -0.5)	2.5 (2.4, 2.6)	-1.1 (-1.2, -0.9)
India	-0.5 (-0.9, -0.1)	3.0 (2.3, 3.7)	-1.1 (-1.5, -0.7)
Maldives	-4.5 (-4.9, -4.1)	-0.7 (-1.1, -0.3)	-4.6 (-5.0, -4.1)
Nepal	-0.2 (-0.3, -0.1)	4.2 (4.1, 4.3)	-0.6 (-0.7, -0.5)
Pakistan	0.9 (0.8, 0.9)	3.8 (3.7, 4.0)	0.2 (0.1, 0.4)
Sri Lanka	-2.1 (-2.7, -1.5)	-0.1 (-0.6, 0.5)	-3.4 (-4.1, -2.7)

**China-ASEAN FTA**			
Brunei Darussalam	-2.3 (-2.6, -2.0)	-0.2 (-0.6, 0.3)	-3.5 (-4.1, -3.0)
Cambodia	-1.4 (-1.5, -1.3)	2.1 (2.0, 2.3)	-1.0 (-1.1, -0.9)
China	-1.0 (-1.5, -0.6)	2.0 (1.7, 2.3)	-0.5 (-0.9, -0.1)
Indonesia	0.1 (-0.1, -0.1)	3.9 (3.9, 4.0)	0.7 (0.6, 0.7)
Laos	-1.8 (-1.9, -1.7)	2.7 (2.6, 2.8)	-1.7 (-1.8, -1.6)
Malaysia	-1.0 (-1.5, -0.5)	1.2 (0.7, 1.8)	-1.0 (-1.5, -0.5)
Myanmar	-2.4 (-2.5, -2.2)	1.7 (1.7, 1.8)	-3.4 (-3.6, -3.3)
Philippines	2.6 (2.1, 3.2)	4.9 (4.4, 5.5)	2.4 (2.1, 2.7)
Singapore	-4.7 (-4.9, -4.4)	-2.3 (-2.6, -2.1)	-5.3 (-5.6, -5.0)
Thailand	-2.2 (-2.8, -1.6)	1.0 (0.5, 1.5)	-2.7 (-3.4, -2.0)
Viet Nam	-1.5 (-1.6, -1.4)	3.0 (2.9, 3.1)	-0.5 (-0.6, -0.4)
**EEU**			
Armenia	-1.1 (-1.6, -0.5)	0.7 (0.1, 1.3)	-0.9 (-1.4, -0.4)
Belarus	0.3 (-0.4, 0.9)	1.3 (0.6, 2.1)	0.6 (-0.2, 1.4)
Kazakhstan	-1.8 (-2.3, -1.2)	-0.6 (-1.2, -0.1)	-1.5 (-2.0, -1.0)
Kyrgyzstan	-0.3 (-1.1, 0.5)	0.5 (-0.3, 1.4)	-0.1 (-0.9, 0.6)
Russia	-0.8 (-1.8, 0.3)	0.3 (-1.1, 1.8)	-0.1 (-1.3, 1.3)
**Mercosur**			

Argentina	-2.5 (-2.9, -2.0)	-1.0 (-1.3, -0.8)	-2.8 (-3.1, -2.6)
Bolivia	-1.6 (-1.8, -1.4)	-0.1 (-0.2, 0.1)	-3.2 (-3.3, -3.1)
Brazil	-2.5 (-2.7, -2.4)	-0.9 (-1.2, -0.6)	-3.5 (-3.7, -3.2)
Paraguay	-0.6 (-1.1, -0.1)	0.5 (0.1, 1.0)	-1.1 (-1.7, -0.6)
Uruguay	-2.8 (-3.1, -2.5)	-1.5 (-1.8, -1.1)	-2.7 (-2.9, -2.4)
Venezuela	-1.2 (-1.9, -0.4)	-0.6 (-1.6, 0.4)	-2.8 (-3.5, -2.2)

Note: AAPC, average annual percent change; IHD, ischemic heart diseases; BRICS, Brazil, Russia, India, China, and South Africa; SACU, South African Customs Union; SAARC, South Asian Association for Regional Cooperation; China-ASEAN FTA, China-ASEAN Free Trade Area; and EEU, Eurasian Economic Union.

Table S12: The average annual percent change in the age-specific (50-69 years) IS mortality for both sexes attributed to modifiable risk factors across BRICS-Plus from 1990 to 2019.

**Population**	**IS (50-69 years)/100,000**		
	Dietary risks (AAPC (95%CI)	High BMI (AAPC (95%CI)	Smoking (AAPC (95%CI)
**BRICS-Plus**	-1.4 (-1.5, -1.2)	0.5 (0.2, 0.7)	-1.4 (-1.6, -1.3)
**SACU**			
Botswana	-1.0 (-1.2, -0.7)	1.7 (1.5, 1.9)	-1.4 (-1.6, -1.1)
Eswatini	-0.5 (-0.8, -0.2)	0.6 (0.3, 1.0)	-1.8 (-2.0, -1.6)
Lesotho	0.7 (0.5, 0.9)	3.1 (2.8, 3.3)	0.8 (0.5, 1.1)
Namibia	-1.8 (-2.0, -1.6)	0.1 (-0.2, 0.3)	-2.6 (-2.8, -2.5)
South Africa	0.1 (-0.5, 0.7)	0.8 (-0.1, 1.7)	-1.9 (-2.6, -1.3)
**SAARC**			
Afghanistan	0.7 (0.4, 0.9)	2.3 (2.0, 2.5)	2.3 (1.9, 2.7)
Bangladesh	-0.7 (-1.2, -0.1)	4.0 (3.6, 4.4)	-1.2 (-1.7, -0.7)
Bhutan	-0.8 (-1.0, -0.6)	2.3 (2.1, 2.5)	-1.4 (-1.6, -1.2)
India	-0.9 (-1.8, 0.1)	2.8 (2.2, 3.4)	-1.5 (-2.4, -0.6)
Maldives	-4.0 (-4.5, -3.5)	-0.1 (-0.6, 0.4)	-4.0 (-4.5, -3.5)
Nepal	-0.9 (-1.0, -0.8)	3.6 (3.4, 3.8)	-1.2 (-1.3, -1.0)
Pakistan	0.5 (0.5, 0.6)	3.3 (3.0, 3.5)	-0.7 (-0.8, -0.6)
Sri Lanka	-1.6 (-2.1, -1.1)	1.0 (0.5, 1.6)	-2.6 (-3.4, -1.8)

**China-ASEAN FTA**			
Brunei Darussalam	-3.6 (-4.0, -3.2)	-1.4 (-1.9, -1.0)	-5.1 (-5.5, -4.7)
Cambodia	-1.1 (-1.3, -1.0)	2.4 (2.2, 2.7)	-0.9 (-1.0, -0.7)
China	-1.1 (-1.8, -0.5)	1.6 (1.1, 2.1)	-0.8 (-1.2, -0.4)
Indonesia	0.2 (0.1, 0.2)	4.6 (4.5, 4.7)	1.4 (1.3, 1.4)
Laos	-1.3 (-1.4, -1.3)	3.3 (3.1, 3.5)	-1.2 (-1.3, -1.1)
Malaysia	-2.2 (-2.5, -1.9)	0.5 (0.1, 0.9)	-1.8 (-2.3, -1.2)
Myanmar	-1.8 (-1.9, -1.7)	2.9 (2.7, 3.1)	-3.0 (-3.1, -2.9)
Philippines	2.5 (2.0, 3.0)	4.8 (4.1, 5.5)	2.4 (2.0, 2.7)
Singapore	-7.4 (-7.8, -7.0)	-5.2 (-5.5, -4.9)	-8.1 (-8.4, -7.7)
Thailand	-2.3 (-3.1, -1.4)	1.4 (0.7, 2.2)	-2.8 (-3.7, -1.8)
Viet Nam	-0.4 (-0.5, -0.2)	4.4 (4.2, 4.7)	0.3 (0.2, 0.4)
**EEU**			
Armenia	-2.5 (-3.0, -2.0)	-0.8 (-1.3, -0.3)	-2.2 (-2.6, -1.7)
Belarus	-1.3 (-1.9, -0.7)	-0.2 (-0.8, 0.4)	-0.7 (-1.4, -0.1)
Kazakhstan	-1.3 (-1.7, -0.9)	0.1 (-0.4, 0.4)	-0.8 (-1.1, -0.4)
Kyrgyzstan	-1.9 (-2.4, -1.4)	-0.7 (-1.1, -0.3)	-1.0 (-1.7, -0.3)
Russia	-1.7 (-2.5, -0.9)	-0.6 (-1.5, 0.2)	-0.5 (-1.5, 0.6)
**Mercosur**			

Argentina	-3.1 (-3.6, -2.6)	-1.6 (-2.2, -1.1)	-3.5 (-4.1, -3.0)
Bolivia	-1.5 (-1.9, -1.2)	-0.2 (-0.4, 0.1)	-3.3 (-3.6, -3.1)
Brazil	-3.6 (-3.7, -3.4)	-2.4 (-2.5, -2.2)	-5.1 (-5.3, -4.9)
Paraguay	-1.4 (-2.1, -0.8)	-0.3 (-1.2, 0.6)	-2.1 (-2.9, -1.3)
Uruguay	-4.0 (-4.3, -3.8)	-2.6 (-2.8, -2.3)	-3.6 (-3.9, -3.4)
Venezuela	-1.5 (-2.5, -0.5)	-1.0 (-1.6, -0.3)	-3.5 (-4.3, -2.7)

Note: AAPC, average annual percent change; IS, ischemic stroke; BRICS, Brazil, Russia, India, China, and South Africa; SACU, South African Customs Union; SAARC, South Asian Association for Regional Cooperation; China-ASEAN FTA, China-ASEAN Free Trade Area; and EEU, Eurasian Economic Union.

Table S13: The average annual percent change in the age-specific (70-89 years) CVD mortality for both sexes attributed to modifiable risk factors across BRICS-Plus from 1990 to 2019.

**Population**	**CVD (70-89 years)/100,000**		
	Dietary risks (AAPC (95%CI)	High BMI (AAPC (95%CI)	Smoking (AAPC (95%CI)
**BRICS-Plus**	-0.8 (-1.0, -0.6)	1.1 (0.9, 1.2)	-1.4 (-1.6, -1.2)
**SACU**			
Botswana	-0.1 (-0.2, 0.2)	2.6 (2.2, 3.1)	0.8 (-0.8, -0.7)
Eswatini	-0.4 (-0.6, -0.2)	0.7 (0.5, 0.8)	-1.6 (-1.8, -1.4)
Lesotho	0.6 (0.5, 0.7)	3.0 (2.8, 3.1)	0.5 (0.3, 0.7)
Namibia	-0.1 (-0.2, -0.1)	1.2 (1.1, 1.4)	-1.5 (-1.6, -1.4)
South Africa	0.2 (-0.4, 0.9)	1.3 (0.6, 2.1)	-2.2 (-2.8, -1.7)
**SAARC**			
Afghanistan	-0.6 (-0.7, -0.5)	1.4 (1.2, 1.7)	0.1 (-0.1, 0.1)
Bangladesh	-0.2 (-1.1, 0.6)	3.0 (2.5, 3.5)	-1.4 (-2.0, -0.8)
Bhutan	0.2 (0.1, 0.3)	2.5 (2.4, 2.6)	-0.4 (-0.5, -0.3)
India	-0.7 (-1.7, 0.3)	2.1 (1.6, 2.7)	-1.5 (-2.4, -0.7)
Maldives	-2.2 (-2.6, -1.8)	0.7 (0.3, 1.1)	-3.1 (-3.4, -2.7)
Nepal	0.2 (0.1, 0.2)	3.7 (3.6, 3.8)	-0.6 (-0.7, -0.5)
Pakistan	0.5 (0.4, 0.5)	2.9 (2.8, 3.1)	-0.6 (-0.7, -0.5)
Sri Lanka	-2.0 (-2.5, -1.4)	1.3 (1.0, 1.6)	-4.0 (-4.6, -3.4)

**China-ASEAN FTA**			
Brunei Darussalam	-1.9 (-2.2, -1.6)	-0.1 (-0.4, 0.2)	-3.7 (-4.1, -3.3)
Cambodia	-0.5 (-0.6, -0.5)	2.9 (2.8, 2.9)	-0.4 (-0.4, -0.3)
China	-0.6 (-1.0, -0.3)	1.3 (0.9, 1.7)	-0.3 (-0.6, 0.1)
Indonesia	0.2 (0.1, 0.2)	3.5 (3.4, 3.6)	1.0 (1.0, 1.1)
Laos	-0.6 (-0.7, -0.6)	2.7 (2.7, 2.8)	-1.1 (-1.1, -1.0)
Malaysia	-1.3 (-2.0, -0.7)	1.2 (0.4, 2.1)	-1.6 (-2.2, -1.0)
Myanmar	-1.1 (-1.1, -1.0)	2.8 (2.7, 2.9)	-2.9 (-2.9, -2.8)
Philippines	0.9 (0.5, 1.2)	4.1 (3.5, 4.8)	0.8 (0.5, 1.0)
Singapore	-3.9 (-4.3, -3.5)	-1.4 (-1.8, -0.9)	-5.1 (-5.4, -4.9)
Thailand	-2.6 (-3.0, -2.2)	0.8 (0.3, 1.2)	-4.0 (-4.8, -3.3)
Viet Nam	-0.7 (-0.7, -0.6)	3.5 (3.3, 3.6)	-0.2 (-0.3, -0.2)
**EEU**			
Armenia	-1.3 (-1.7, -1.0)	0.5 (0.3, 0.8)	-1.3 (-1.6, -1.0)
Belarus	-0.3 (-1.0, 0.4)	1.0 (0.3, 1.7)	0.1 (-0.5, 0.7)
Kazakhstan	-0.9 (-1.5, -0.4)	0.2 (-0.3, 0.6)	-0.4 (-0.6, -0.1)
Kyrgyzstan	0.7 (-0.2, 1.7)	1.0 (0.3, 1.7)	0.4 (-0.2, 1.0)
Russia	-1.1 (-1.9, -0.3)	0.1 (-0.6, 0.9)	0.2 (-0.7, 1.1)
**Mercosur**			

Argentina	-2.5 (-2.8, -2.2)	-0.7 (-1.1, -0.3)	-3.0 (-3.3, -2.7)
Bolivia	-1.1 (-1.2, -1.0)	1.3 (1.2, 1.4)	-3.3 (-3.5, -3.2)
Brazil	-2.6 (-2.8, -2.4)	-0.7 (-0.9, -0.4)	-4.3 (-4.5, -4.0)
Paraguay	-1.0 (-1.4, -0.6)	0.6 (0.1, 1.0)	-1.7 (-2.2, -1.3)
Uruguay	-2.5 (-2.9, -2.1)	-0.8 (-1.2, -0.4)	-2.7 (-3.2, -2.1)
Venezuela	-1.0 (-1.9, -0.1)	-0.2 (-0.9, 0.5)	-3.0 (-3.9, -2.0)

Note: AAPC, average annual percent change; CVD, cardiovascular diseases; BRICS, Brazil, Russia, India, China, and South Africa; SACU, South African Customs Union; SAARC, South Asian Association for Regional Cooperation; China-ASEAN FTA, China-ASEAN Free Trade Area; and EEU, Eurasian Economic Union.

Table S14: The average annual percent change in the age-specific (70-89 years) IHD mortality for both sexes attributed to modifiable risk factors across BRICS-Plus from 1990 to 2019.

**Population**	**IHD (70-89 years)/100,000**		
	Dietary risks (AAPC (95%CI)	High BMI (AAPC (95%CI)	Smoking (AAPC (95%CI)
**BRICS-Plus**	-0.6 (-0.8, -0.4)	1.1 (0.9, 1.3)	-1.2 (-1.4, -1.0)
**SACU**			
Botswana	0.4 (0.2, 0.5)	3.3 (2.8, 3.7)	-0.3 (-0.4, -0.3)
Eswatini	-0.3 (-0.6, -0.1)	1.0 (0.8, 1.2)	-1.3 (-1.5, -1.1)
Lesotho	0.8 (0.7, 0.9)	3.4 (3.2, 3.5)	0.7 (0.5, 0.9)
Namibia	0.2 (0.1, 0.3)	1.6 (1.4, 1.7)	-1.3 (-1.4, -1.2)
South Africa	0.2 (-0.4, 0.8)	1.4 (0.7, 2.1)	-2.2 (-2.7, -1.6)
**SAARC**			
Afghanistan	-0.7 (-0.7, -0.6)	1.3 (1.0, 1.5)	-0.1 (-0.2, 0.1)
Bangladesh	0.2 (-0.6, 1.0)	3.6 (3.1, 4.2)	-1.1 (-1.7, -0.5)
Bhutan	0.6 (0.5, 0.7)	3.4 (3.3, 3.6)	0.1 (-0.1, 0.2)
India	-0.4 (-1.6, 0.9)	2.7 (2.2, 3.2)	-1.2 (-2.3, -0.1)
Maldives	-2.1 (-2.5, -1.7)	0.7 (0.3, 1.1)	-3.2 (-3.6, -2.7)
Nepal	0.6 (0.5, 0.8)	4.5 (4.4, 4.6)	0.1 (-0.1, 0.1)
Pakistan	0.7 (0.6, 0.8)	3.5 (3.4, 3.7)	-0.1 (-0.2, 0.1)
Sri Lanka	-1.8 (-2.5, -1.1)	1.2 (0.7, 1.7)	-3.8 (-4.4, -3.3)

**China-ASEAN FTA**			
Brunei Darussalam	-1.7 (-2.2, -1.1)	0.2 (-0.3, 0.7)	-3.4 (-3.9, -3.0)
Cambodia	-0.2 (-0.3, -0.2)	3.3 (3.2, 3.3)	-0.1 (-0.2, -0.1)
China	0.7 (0.4, 1.0)	3.4 (3.1, 3.6)	1.0 (0.7, 1.4)
Indonesia	0.5 (0.5, 0.6)	3.7 (3.6, 3.9)	1.1 (1.0, 1.1)
Laos	-0.4 (-0.5, -0.3)	3.1 (3.0, 3.2)	-1.0 (-1.0, -0.9)
Malaysia	-0.9 (-1.4, -0.3)	2.0 (1.4, 2.6)	-1.2 (-1.8, -0.6)
Myanmar	-1.0 (-1.1, -1.0)	2.7 (2.6, 2.8)	-2.9 (-3.0, -2.8)
Philippines	1.0 (0.8, 1.3)	3.9 (3.3, 4.5)	1.0 (0.7, 1.3)
Singapore	-3.6 (-4.0, -3.3)	-1.5 (-1.8, -1.2)	-5.0 (-5.3, -4.6)
Thailand	-2.2 (-2.6, -1.7)	1.3 (0.8, 1.8)	-3.6 (-4.6, -2.6)
Viet Nam	-0.6 (-0.7, -0.4)	3.8 (3.7, 3.9)	-0.2 (-0.2, -0.1)
**EEU**			
Armenia	-1.2 (-1.6, -0.7)	0.6 (0.1, 1.0)	-1.2 (-1.5, -1.0)
Belarus	-0.2 (-1.1, 0.7)	1.1 (0.5, 1.7)	0.3 (-0.3, 0.9)
Kazakhstan	-1.0 (-1.6, -0.4)	-0.1 (-0.6, 0.3)	-0.6 (-0.9, -0.3)
Kyrgyzstan	1.1 (0.3, 1.9)	2.0 (1.2, 2.7)	1.2 (0.6, 1.9)
Russia	-0.9 (-1.5, -0.3)	0.3 (-0.5, 1.1)	0.2 (-0.9, 1.3)
**Mercosur**			

Argentina	-2.6 (-2.9, -2.3)	-1.2 (-1.7, -0.7)	-3.1 (-3.4, -2.8)
Bolivia	-1.0 (-1.1, -0.9)	1.4 (1.3, 1.5)	-3.2 (-3.3, -3.1)
Brazil	-2.8 (-3.0, -2.6)	-0.8 (-1.1, -0.5)	-4.3 (-4.5, -4.1)
Paraguay	-0.8 (-1.3, -0.4)	0.7 (0.2, 1.1)	-1.5 (-1.9, -1.0)
Uruguay	-2.8 (-3.2, -2.4)	-1.6 (-2.1, -1.2)	-3.2 (-3.8, -2.5)
Venezuela	-1.0 (-1.9, -0.1)	-0.1 (-0.7, 0.5)	-3.0 (-4.0, -2.0)

Note: AAPC, average annual percent change; IHD, ischemic heart diseases; BRICS, Brazil, Russia, India, China, and South Africa; SACU, South African Customs Union; SAARC, South Asian Association for Regional Cooperation; China-ASEAN FTA, China-ASEAN Free Trade Area; and EEU, Eurasian Economic Union.

Table S15: The average annual percent change in the age-specific (70-89 years) IS mortality for both sexes attributed to modifiable risk factors across BRICS-Plus from 1990 to 2019.

**Population**	**IS (70-89 years)/100,000**		
	Dietary risks (AAPC (95%CI)	High BMI (AAPC (95%CI)	Smoking (AAPC (95%CI)
**BRICS-Plus**	-0.9 (-1.1, -0.7)	0.7 (0.6, 0.9)	-1.4 (-1.6, -1.2)
**SACU**			
Botswana	-0.1 (-0.3, 0.1)	2.5 (2.1, 3.0)	-0.8 (-0.8, -0.7)
Eswatini	-0.4 (-0.6, -0.2)	0.7 (0.5, 0.9)	-1.6 (-1.8, -1.3)
Lesotho	0.8 (0.7, 0.9)	3.1 (3.0, 3.3)	0.6 (0.5, 0.8)
Namibia	-0.1 (-0.2, -0.1)	1.1 (1.0, 1.3)	-1.4 (-1.5, -1.3)
South Africa	1.0 (0.1, 1.9)	2.0 (0.9, 3.1)	-1.9 (-2.7, -1.1)
**SAARC**			
Afghanistan	0.7 (0.6, 0.8)	2.6 (2.4, 2.8)	1.5 (1.3, 1.6)
Bangladesh	0.1 (-0.8, 1.0)	3.4 (2.8, 3.9)	-1.2 (-2.0, -0.4)
Bhutan	-0.2 (-0.3, -0.1)	2.4 (2.3, 2.5)	-0.8 (-1.0, -0.6)
India	-1.3 (-2.2, -0.5)	2.0 (1.2, 2.7)	-1.9 (-2.7, -1.1)
Maldives	-1.3 (-1.8, -0.9)	1.2 (0.7, 1.7)	-2.0 (-2.3, -1.7)
Nepal	-0.5 (-0.6, -0.4)	3.4 (3.2, 3.5)	-1.0 (-1.1, -0.9)
Pakistan	0.3 (0.2, 0.3)	3.0 (2.8, 3.1)	-1.2 (-1.2,-1.1)
Sri Lanka	-2.4 (-2.8, -2.0)	0.7 (0.3, 1.1)	-4.2 (-4.7, -3.7)

**China-ASEAN FTA**			
Brunei Darussalam	-2.6 (-2.9, -2.2)	-0.7 (-1.1, -0.4)	-4.4 (-4.8, -4.0)
Cambodia	-0.1 (-0.2, -0.1)	3.2 (3.1, 3.2)	-0.1 (-0.1, 0.1)
China	-0.1 (-0.5, 0.4)	2.1 (1.7, 2.5)	0.3 (-0.2, 0.7)
Indonesia	0.6 (0.6, 0.7)	4.2 (4.1, 4.4)	1.6 (1.5, 1.7)
Laos	-0.2 (-0.3, -0.2)	3.1 (3.1, 3.2)	-0.7 (-0.8, -0.6)
Malaysia	-1.8 (-2.8, -0.8)	0.9 (-0.2, 2.0)	-2.0 (-3.1, -1.0)
Myanmar	-0.4 (-0.4, -0.3)	3.5 (3.3, 3.7)	-2.3 (-2.3, -2.2)
Philippines	0.6 (0.2, 1.0)	3.9 (3.4, 4.3)	0.6 (0.3, 0.9)
Singapore	-5.4 (-5.9, -5.0)	-3.7 (-4.2, -3.3)	-6.9 (-7.3, -6.5)
Thailand	-2.2 (-2.6, -1.8)	1.0 (0.4, 1.6)	-3.7 (-4.6, -2.9)
Viet Nam	0.2 (0.1, 0.4)	4.4 (4.1, 4.6)	0.3 (0.3, 0.4)
**EEU**			
Armenia	-2.4 (-3.5, -1.4)	-0.5 (-1.3, 0.3)	-2.2 (-3.2, -1.2)
Belarus	-0.9 (-1.4, -0.5)	0.1 (-0.3, 0.4)	-0.6 (-1.1, -0.2)
Kazakhstan	-0.7 (-1.1, -0.3)	0.2 (-0.1, 0.5)	-0.1 (-0.4, 0.2)
Kyrgyzstan	-2.4 (-3.7, -1.1)	-1.3 (-1.9, -0.7)	-1.8 (-2.6, -1.0)
Russia	-1.6 (-2.2, -1.1)	-0.7 (-1.2, -0.2)	-0.4 (-1.3, 0.5)
**Mercosur**			

Argentina	-2.6 (-2.9, -2.3)	-1.3 (-1.6, -0.9)	-3.1 (-3.4, -2.8)
Bolivia	-0.9 (-1.0, -0.8)	1.4 (1.2, 1.5)	-3.3 (-3.5, -3.2)
Brazil	-2.5 (-2.8, -2.1)	-1.2 (-1.4, -1.0)	-4.8 (-5.0, -4.6)
Paraguay	-1.1 (-1.6, -0.7)	0.2 (-0.7, 1.1)	-1.9 (-2.5, -1.3)
Uruguay	-2.3 (-2.7, -2.0)	-1.3 (-1.7, -0.8)	-2.6 (-3.0, -2.3)
Venezuela	-0.8 (-1.6, 0.1)	-0.3 (-0.9, 0.4)	-3.1 (-3.9, -2.2)

Note: AAPC, average annual percent change; IS, ischemic stroke; BRICS, Brazil, Russia, India, China, and South Africa; SACU, South African Customs Union; SAARC, South Asian Association for Regional Cooperation; China-ASEAN FTA, China-ASEAN Free Trade Area; and EEU, Eurasian Economic Union.
